# Rif1 Regulates Initiation Timing of Late Replication Origins throughout the *S. cerevisiae* Genome

**DOI:** 10.1371/journal.pone.0098501

**Published:** 2014-05-30

**Authors:** Jared M. Peace, Anna Ter-Zakarian, Oscar M. Aparicio

**Affiliations:** 1 Molecular and Computational Biology Program, University of Southern California, Los Angeles, California, United States of America; 2 Program in Global Medicine, Keck School of Medicine, University of Southern California, Los Angeles, California, United States of America; Universita’ di Milano, Italy

## Abstract

Chromosomal DNA replication involves the coordinated activity of hundreds to thousands of replication origins. Individual replication origins are subject to epigenetic regulation of their activity during S-phase, resulting in differential efficiencies and timings of replication initiation during S-phase. This regulation is thought to involve chromatin structure and organization into timing domains with differential ability to recruit limiting replication factors. Rif1 has recently been identified as a genome-wide regulator of replication timing in fission yeast and in mammalian cells. However, previous studies in budding yeast have suggested that Rif1’s role in controlling replication timing may be limited to subtelomeric domains and derives from its established role in telomere length regulation. We have analyzed replication timing by analyzing BrdU incorporation genome-wide, and report that Rif1 regulates the timing of late/dormant replication origins throughout the *S. cerevisiae* genome. Analysis of *pfa4Δ* cells, which are defective in palmitoylation and membrane association of Rif1, suggests that replication timing regulation by Rif1 is independent of its role in localizing telomeres to the nuclear periphery. Intra-S checkpoint signaling is intact in *rif1Δ* cells, and checkpoint-defective *mec1Δ* cells do not comparably deregulate replication timing, together indicating that Rif1 regulates replication timing through a mechanism independent of this checkpoint. Our results indicate that the Rif1 mechanism regulates origin timing irrespective of proximity to a chromosome end, and suggest instead that telomere sequences merely provide abundant binding sites for proteins that recruit Rif1. Still, the abundance of Rif1 binding in telomeric domains may facilitate Rif1-mediated repression of non-telomeric origins that are more distal from centromeres.

## Introduction

Eukaryotic chromosomal DNA replication involves the coordinated activity of hundreds to many thousands (depending on the organism) of DNA replication origins distributed along chromosomes that initiate the replication process locally (reviewed in [1]). In G1-phase, replication origins are “licensed” for replication by loading of replicative DNA helicases, in their inactive state, onto origin DNA (reviewed in [2]). Replication initiation involves helicase activation and recruitment of DNA Polymerases triggered by the sequential activities of Dbf4-dependent kinase (DDK) and Cyclin-dependent kinase, both of which are under cell cycle control, to regulate S-phase entry (reviewed in [3]). Despite these shared molecular requirements for initiation, individual origins exhibit reproducible differences in their initiation kinetics resulting in characteristic origin initiation times and/or efficiencies, where efficiency refers to the frequency of activation versus “passive” replication of the origin without initiation. Consequently, different chromosomal regions exhibit differential replication timing during S-phase (reviewed in [4]). In general, heterochromatic regions are late-replicating, suggesting a role for chromatin structure in regulating replication origin activity. Replication timing and transcriptional state may be mutually reinforcing, which has been proposed to provide a mechanism for epigenetic inheritance through cell cycles (reviewed in [5]). In addition, the typically early replication of expressed, euchromatic genes may allow for more rapid accumulation of transcripts as two copies of template become available earlier in the cell cycle. Furthermore, early replication is associated with lower mutation rates (reviewed in [6]). Hence, differential replication timing potentially contributes to cellular differentiation by maximizing expression and integrity of critical genes, while also contributing to proper development and maintenance of organismal homeostasis by contributing to epigenetic inheritance of cellular identity.

In the budding yeast *S. cerevisiae*, DNA sequences near the ends of chromosomes, including the telomeres themselves, the adjacent “subtelomeric” regions, and the subtelomere-proximal silent mating-type loci exhibit hallmarks of heterochromatin, including characteristic chromatin modifications, transcriptional silencing and late replication (reviewed in [7]). Replication origins in these regions either initiate replication late during S-phase, or fail to initiate before being replicated passively by replication forks from earlier origins, in which case they are termed “dormant” (reviewed in [4]). Disruption of telomeric heterochromatin in *sir3Δ* mutant cells results in earlier firing of late and/or dormant origins, indicating the involvement of chromatin structure in regulating origin activation [8]. Similarly, the histone deacetylase Rpd3 is responsible for delaying the initiation of many, if not most, internal, late-firing origins, again supporting the idea that chromatin structure regulates origin firing [9]–[11]. Nevertheless, the exact mechanism(s) delaying initiation remains vague. Additionally, late firing may reflect a lack of positive stimulation of origin firing in the context of competition for a limiting quantity of initiation factors. Indeed, recent studies have revealed diverse mechanisms to stimulate early origin firing by recruiting limiting initiation factors to specific origins [12], [13].

Rif1 (Rap1-interacting factor 1) has recently been implicated in the regulation of replication origin timing in yeast and mammalian genomes [14]–[17]. Rif1 binds telomeres and subtelomeres through direct interaction with the telomere sequence binding protein Rap1 in *S. cerevisiae* or Taz1 in *S. pombe* [18], [19]. In mammalian cells, however, Rif1 is recruited to telomeres and other chromosomal loci upon DNA damage, where it functions in signaling and repair of DNA damage (reviewed in [20]). In the fission yeast *S. pombe*, *rif1^+^* deletion advances the timing of many late/dormant origins in subtelomeric as well as internal chromosomal loci, while also delaying or repressing the activation of normally early origins, including pericentric origins [15]. The effects of Rif1 deletion in mouse cells or depletion in human cells appear to be similar to those in fission yeast, with advanced timing of late domains together with delayed replication of early domains resulting in an overall compression of the temporal replication program [14], [16]. Analysis of budding yeast *rif1Δ* cells showed advanced replication timing of subtelomeric regions, relative to an internal early and an internal late origin, suggesting that origin regulation by Rif1 in budding yeast might be limited to subtelomeric domains [17]. However, these data also appear to indicate that the internal early origin used as a standard (ARS1) was delayed, and happens to reside near a centromere, consistent with the possibility that early origin timing, particularly of pericentric regions, is regulated by Rif1 in budding yeast as in fission yeast. This might also explain how pericentric origins remain early-firing in the absence of transcription factors Fkh1 and Fkh2, which are required for early-firing of most non-pericentric early origins in budding yeast [12].

The mechanism of Rif1 function in origin regulation also requires further elucidation. Rif1 regulates telomere length, which has been implicated in the control of telomere replication timing in *S. cerevisiae* (reviewed in [21]). Thus, Rif1’s effect on subtelomeric replication timing has been attributed to its function in controlling telomere length. However, this mechanism does not easily account for effects of Rif1 at internal chromosomal loci as observed in *S. pombe* [15]. In both yeasts, Rif1 binds internal chromosomal loci independently of its telomere-binding partner Rap1 or Taz1, although the significance of this binding has not been carefully examined [15], [22]. Rif1 may perform a scaffolding function to organize or localize chromatin domains. In mammalian cells, Rif1 fractionates with the insoluble nuclear scaffold, and the structure of Rif1 (in yeast and mammals) contains motifs that mediated protein-protein interaction, consistent with a scaffolding function [14], [16], [23]. In *S. cerevisiae*, palmitoylation of Rif1 is required for localization of telomeres to the nuclear periphery, which is typically associated with late replication [24]. Thus, Rif1’s function in telomere localization may contribute to its role in origin timing, at least in yeast. Whether Rif1 plays a similar function to localize internal chromosomal loci to the nuclear periphery remains to be determined.

In this study we have addressed the role of Rif1 in regulation of replication origin timing in budding yeast by analyzing replication genome-wide in cells lacking Rif1 function. We have also addressed the importance of palmitoylation of Rif1 in replication timing control by analyzing replication in cells lacking Pfa4, which is required for Rif1 palmitoylation and its localization to the nuclear periphery [24]. Our findings demonstrate that Rif1 plays a global role in the regulation of late/dormant origins throughout the budding yeast genome, while palmitoylation is not required for this function of Rif1. Intra-S checkpoint signaling, which modulates replication initiation in response to replication stress, is intact in *rif1Δ* cells, suggesting that Rif1 regulates origin timing through a distinct mechanism. These findings indicate that Rif1’s function in origin timing regulation is mechanistically independent of telomere proximity.

## Results

### Rif1 Regulates Origin Firing Independently of Pfa4

**Figure 1:**
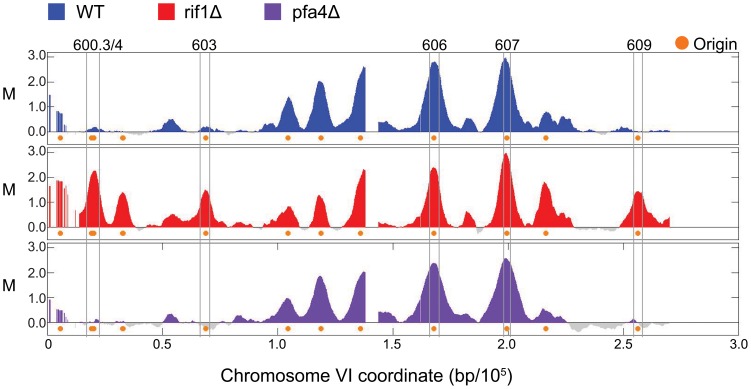
Analysis of early S-phase by BrdU-chip. Plots show BrdU incorporation in HU-arrested cells. Plot colors are keyed above. Boxed origins are labeled and discussed in the text.

To examine the role of Rif1 in controlling replication timing of the yeast genome, we began by analyzing origin firing in in the presence of hydroxyurea (HU), which arrests cells in early S-phase after early origin firing while inhibiting unfired (late or dormant) origins through intra-S checkpoint signaling [25]. Thus in *WT* cells, early origins fire efficiently in HU while later origins fire inefficiently, whereas conditions or mutations that alter replication timing or intra-S checkpoint signaling result in changes to the HU replication profile [9], [10], [25]. Our previous studies show that bromodeoxyuridine (BrdU) incorporation levels at origins in HU-arrested cells are inversely related to those origins’ replication timings (T_Rep_) in untreated cells [10], [12]. We released G1-synchronized *WT* and *rif1Δ* cells into S-phase in the presence of BrdU and HU, and detected BrdU incorporation into nascent DNA using BrdU-immunoprecipitation (IP) analyzed with tiling DNA microarrays (BrdU-IP-chip). Budding morphology and DNA content analyses show that *WT* and *rif1Δ* cells entered S-phase with indistinguishable kinetics and arrested DNA synthesis with indistinguishable DNA contents (data not shown). In *WT* cells, BrdU was robustly detected at early origins, while its incorporation at later-firing origins was substantially reduced, as expected (Fig. 1). For example, plots of data for chromosome VI show a strong BrdU signal at early origins (e.g.: *ARS606, ARS607*) in comparison to weak BrdU signal at later origins (e.g.: *ARS600.3/4*, *ARS603*, and *ARS609*). In *rif1Δ* cells, strong BrdU signals, comparable to those in *WT* cells, are observed at early and late/dormant origins, including subtelomeric and internal origins, on chromosome VI and throughout the genome (Fig. 1 and S1). Thus, Rif1 regulates the activation of late/dormant origins in response to HU throughout the budding yeast genome.

To ascertain whether the function of Rif1 in regulating origin firing in HU depends on the putative role of Rif1 in anchoring DNA sequences to the nuclear envelope, we analyzed origin firing in *pfa4Δ* cells that are defective in palmitoylation of Rif1, which is required for its localization, and that of telomeres, to the nuclear periphery, presumably through anchoring of palmitoylated Rif1 to the inner nuclear membrane [24]. G1-synchronized *pfa4Δ* cells were released synchronously into S-phase in the presence of HU and analyzed using BrdU-IP-chip. Budding morphology and DNA content analyses show that *pfa4Δ* cells entered S-phase with similar timing and arrested DNA synthesis with similar DNA content as *WT* cells (data not shown). In the BrdU-IP-chip analysis, cells lacking *PFA4* exhibited a replication profile indistinguishable from that of *WT* cells (Fig. 1 and S1). Thus, palmitoylation of Rif1 by Pfa4 is not required for Rif1’s function in regulating replication origin firing.

### Rif1 Regulates Replication Origin Timing

**Figure 2:**
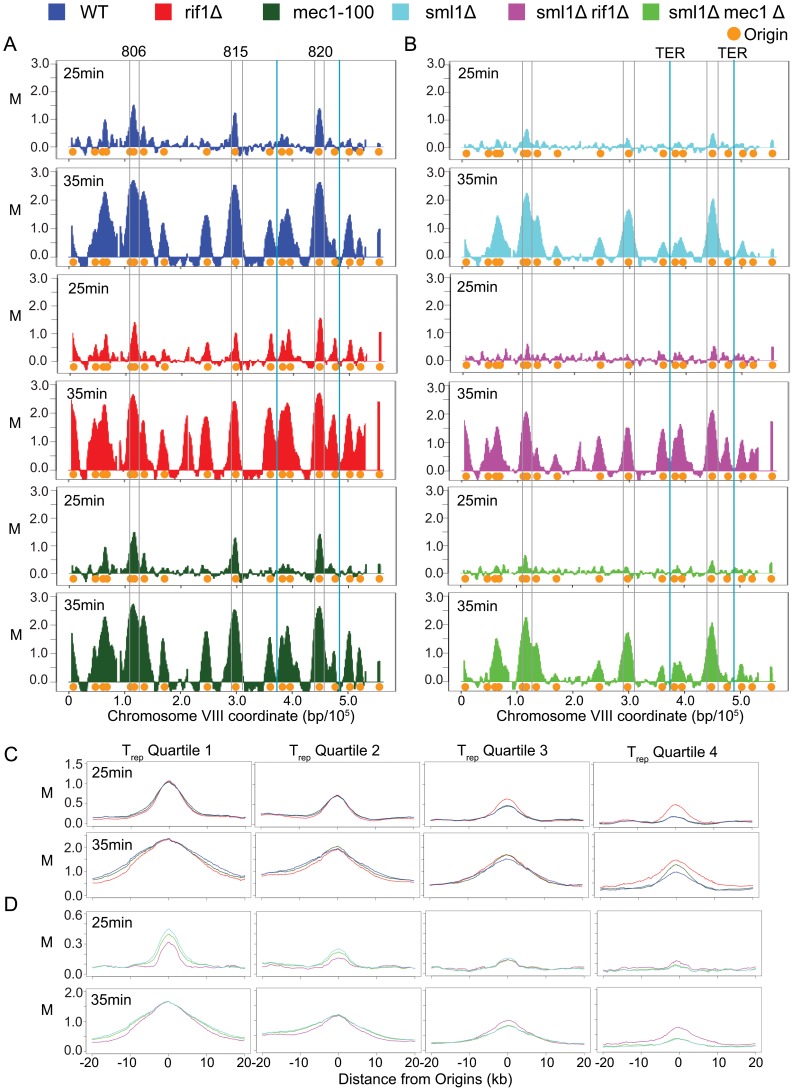
Temporal analysis of replication by BrdU-IP-chip. Plot colors are keyed above. (**A, B**) Plots show average BrdU incorporation from duplicate experiments. Boxed origins and termination sites (TER) are labeled and discussed in the text. (**C, D**) Plots show average BrdU incorporation signals centered on origins in each T_Rep_ quartile.

Previous studies have shown that earlier firing of late/dormant origins can enable their activation in HU in otherwise checkpoint-proficient cells [9]. However, the global firing in *rif1Δ* cells of late/dormant origins in HU is also consistent with deregulation of the intra-S checkpoint’s function in origin inhibition. To determine whether Rif1 regulates replication origin timing in the absence of checkpoint activation by HU, we analyzed replication timing in *WT* and *rif1Δ* cells progressing synchronously through S-phase using BrdU-IP-chip. G1-synchronized cells were released into S-phase in the presence of BrdU and cells were harvested at 25 min and 35 min after release to examine temporal replication profiles. Bulk DNA content analysis shows that *WT* and *rif1Δ* cells progressed through S-phase with indistinguishable kinetics (Fig. S2). At 25 min, both *WT* and *rif1Δ* cells showed low levels of BrdU incorporation at representative very early origins *ARS806, ARS815*, *ARS820* consistent with cells of both strains having entered S-phase simultaneously (Fig. 2A and S3). In *WT* cells, as expected at this early S-phase time point, BrdU incorporation signals were low or undetectable at most other origins on this chromosome, consistent with their later activation. However, *rif1Δ* cells showed substantial BrdU incorporation at many additional origin loci at 25 min, indicating that these loci initiate replication earlier, relative to the earliest origins, than in *WT* cells (Fig. 2A and S3). By 35 min, robust BrdU incorporation was detected at (and surrounding) early and late origins in both *WT* and *rif1Δ* cells, while the convergence (or greater convergence) at termination (TER) sites of some BrdU-labeled replicons from later origins reflects the earlier firing of these origins in *rif1Δ* cells (Fig. 2A).

We examined these data genome-wide by dividing origins into replication timing quartiles according to their published T_Rep_ and plotting the average BrdU-IP signal, centered on the ARSs, for both time points (Fig. 2C). This analysis shows that *RIF1* deletion affected the level of BrdU incorporation at origins at 25 min in the two later timing quartiles, while earlier origins were unaffected. By 35 min, incorporation at the later origins was observed in *WT* cells, but still trailed the levels in *rif1Δ* cells. Levels at the earlier origins showed no effect of *RIF1* deletion at 25 or 35 min. These finding demonstrate that Rif1 specifically regulates the activation timing of subtelomeric and internal late origins during normal S-phase progression.

### Rif1 and Mec1 Regulate Replication Timing through Distinct Pathways

Mutation of intra-S checkpoint regulator *RAD53* has been reported to advance activation timing of a late origin in untreated cells, raising the possibility that Rif1 regulates replication timing as a mediator of the intra-S checkpoint. To determine whether elimination of intra-S checkpoint signaling might explain the altered replication timing in *rif1Δ* cells, we examined replication timing in *mec1-100* mutant cells, which are defective in late origin regulation through the intra-S checkpoint [26]. This analysis was conducted identically to and in parallel with the *WT* and *rif1Δ* analyses presented in the previous section. Analysis of bulk DNA content shows that *mec1-100* cells progressed through an unperturbed S-phase with similar overall kinetics as *WT* and *rif1Δ* cells (Fig. S2). BrdU incorporation analysis of *mec1-100* cells shows replication timing profiles similar to *WT* cells, with only the earliest origins showing clear BrdU peaks at 25 min and relative BrdU peak sizes at 35 min reflecting normal timing differences as well (Fig. 2A, C and S3). Interestingly, the average signal at the latest origin quartile was slightly higher in *mec1-100* than in *WT* cells, consistent with the previous report of a partial advancement of late origin timing in *rad53-1* cells [27]. Overall, however, the results indicate that the intra-S checkpoint plays a minor role in maintaining the temporal program of replication origin firing in budding yeast.

To address the possibility that potential residual function of *mec1-100* maintains normal replication timing, we examined replication timing in *mec1Δ* cells. Viability of *mec1Δ* cells depends on an alternative means of upregulating ribonucleotide reductase activity, which can be accomplished by deletion of *SML1*, which inhibits ribonucleotide reductase. Thus, we compared *sml1Δ*, *sml1Δ rif1Δ*, and *sml1Δ mec1Δ* cells in an experiment carried out identically to the previous. The results show qualitatively similar results with advanced timing of many later origins in *sml1Δ ri1Δ* cells, while *sml1Δ mec1Δ* are similar to *sml1Δ* (Fig. 2B, D, S4). Compared with the *WT* cells in the previous analysis (Fig. 2A, C), deletion of *SML1* resulted in a lower BrdU signal at 25 min (Fig. 2B, D), which was likely due to higher endogenous pools of deoxyribonucleotides reducing the effective, initial BrdU concentration. The *sml1Δ* strains also showed slightly reduced synchrony than the *SML1* strains, which may also have contributed to the slightly reduced signals at 25 min (Fig. S2). These results clearly allow us to conclude that the function of Rif1 in replication origin timing control does not reflect a role in intra-S checkpoint signaling.

**Figure 3:**
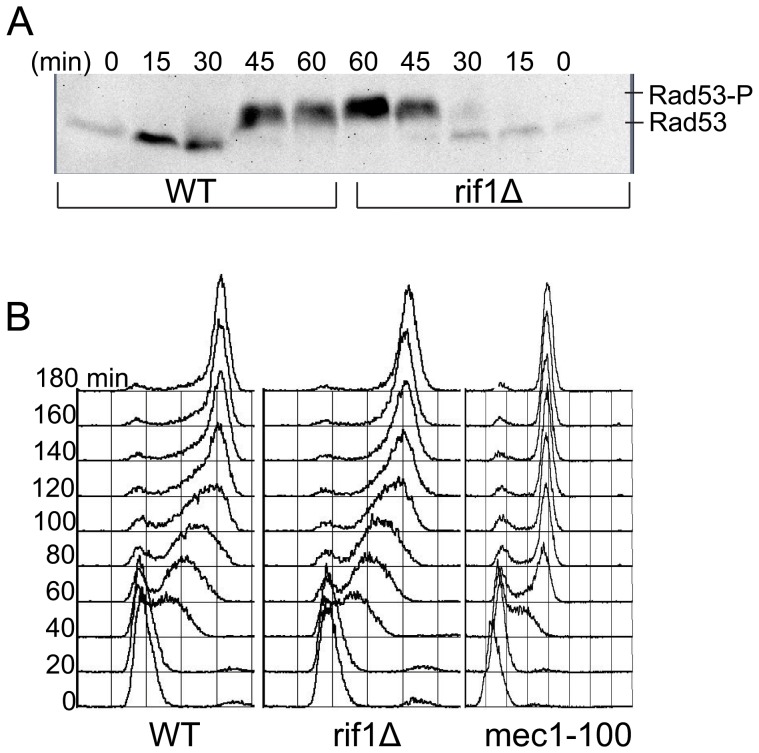
Analysis of intra-S checkpoint response. (**A**) Immunoblot analysis of Rad53 phosphorylation in cells released into HU. (**B**) DNA content analysis of cells released into MMS.

To test the integrity of the intra-S checkpoint in *rif1Δ* cells, we examined two indicators of a functional checkpoint response: Rad53 phosphorylation in response to HU treatment, and slowing of bulk DNA synthesis in the presence of the DNA damaging agent methyl-methansulfonate (MMS) [28]–[30]. First we analyzed Rad53 phosphorylation in *WT* and *rif1Δ* cells released into S-phase in the presence of HU. Rad53 phosphorylation retards its mobility in SDS-PAGE, which can be detected by immunoblotting [31]. In *WT* cells, phosphorylation of Rad53 was apparent as a slower migrating form that began to appear at 30 min and became the predominant form by 45 min after release (Fig. 3A). In *rif1Δ* cells, the timing and degree of phosphorylation of Rad53 were indistinguishable from *WT* under these conditions (Fig. 3A). Thus, intra-S checkpoint signaling to activate Rad53 in response to HU is intact in *rif1Δ* cells.

Next, we analyzed replication slowing in response to MMS treatment. We released G1-synchronized *WT*, *rif1Δ*, and *mec1-100* cells into S-phase in the presence of MMS and analyzed bulk DNA content by flow cytometry. *WT* and *rif1Δ* cells exhibited slow progression of bulk DNA replication, with most cells requiring over 2 hrs to reach fully replicated (2C) DNA content (Fig. 3B). In contrast, *mec1-100* cells attained ∼2C DNA content by 1 hr, reflecting their intra-S checkpoint defect [26], [28]. These results indicate that replication slowing dependent on intra-S checkpoint signaling is functional in *rif1Δ* cells. Taken together, our results demonstrate that Rif1 regulates origin initiation timing throughout the genome, through a mechanism independent of intra-S checkpoint signaling.

### Landscape of Rif1 Function/Rif1 Recruitment

**Figure 4:**
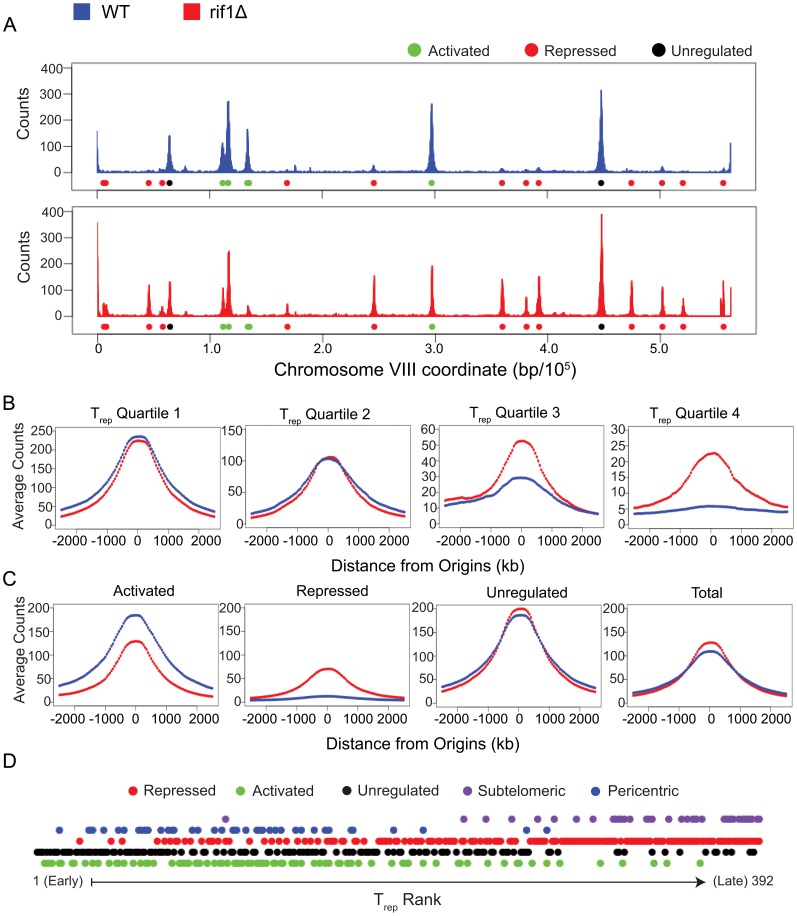
Analysis of early S-phase by BrdU-IP-Seq. Plot colors are keyed above. (**A**) Plots show average BrdU incorporation from duplicate experiments. Origin classes are color-coded below each plot. (**B**) Plots show average BrdU incorporation signals centered on origins in each T_Rep_ quartile. (**C**) Plots show average BrdU incorporation signals centered on origins in each class as described in the text. (**D**) Origins are plotted along the x-axis according to T_Rep_ rank and color-coded according to class and genomic location.

To gain a better understanding of how Rif1 regulates replication timing, we wished to define its target origins for further examination. To facilitate comparison of replication origin activities in *WT* versus *rif1Δ* cells, we repeated the analysis of BrdU incorporation in HU-arrested cells, this time using BrdU-IP analyzed by massively parallel DNA sequencing (BrdU-IP-Seq), which permits more reliable quantification of the immunoprecipitated material and better genome coverage, particularly of pseudo-repetitive sequences, such as subtelomeric regions. The resulting replication profiles recapitulate the previous BrdU-IP-chip results, showing widespread deregulation of normally HU-dormant origins throughout internal and subtelomeric regions in *rif1Δ* cells (Fig. 4A and S5). Genome-wide analysis using MACS shows significant (p<0.01) BrdU incorporation at 241 known origins in HU in *WT* cells versus 373 in *rif1Δ* cells, together accounting for 392 total origins (Table S1). We directly examined the relationship of Rif1 regulation to origin timing by dividing the identified origins into T_Rep_ quartiles and plotting the averaged BrdU signal at each group of origins, centered on the origin sequences (Fig. 4B). The results show similar levels of BrdU incorporation at origins in the earliest two timing quartiles in *WT* and *rif1Δ* cells, while *rif1Δ* cells show markedly higher levels at origins in the later-firing quartiles. These data confirm that Rif1 specifically regulates later-firing origins.

We further examined these data to classify origins according to their changes in signal between *WT* and *rif1Δ* cells. Analysis of 392 total origins using DiffBind (q<0.05) determined that 174 (44%) showed higher signals in *rif1Δ* cells, 137 (35%) showed no change, and 81 (21%) showed lower signals in *rif1Δ* cells; these origins are termed, Rif1-repressed, Rif1-unregulated, and Rif1-activated, respectively (Fig. 4A, C and Table S1). Rif1-activated origins showed a relatively small change in signal magnitude, whereas Rif1-repressed origins showed several-fold greater average signal magnitude in *rif1Δ* cells (Fig. 4C). To reveal the distribution of these origin classes in relation to replication timing, we arranged origins according to their replication timings and annotated the origins by class (Fig. 4D). Consistent with Rif1 regulating later-firing origins genome-wide, the data show that virtually all later-firing origins are in the Rif1-repressed class. Interestingly, Rif1-activated and Rif1-unregulated origins are similarly distributed across the earlier half of origins. These data also show the greater relative effect of Rif1 on later-firing versus earlier-firing origins.

To examine the genomic distribution of Rif1-regulated origins more precisely, we determined the numbers of origins in each Rif1-regulation class with respect to major chromosomal landmarks like centromeres (CENs) and telomeres (TELs). Among 392 origins identified in this study, 41 are within 20 kb of CENs. 15 of these 41 (37%) are classified as Rif1-unregulated, 26 (63%) are Rif1-activated, and zero (0%) are Rif1-repressed (Fig. 4D). Thus, pericentric regions contain an over-representation of Rif1-unregulated and -activated origins and are devoid of Rif1-repressed origins. Similar analysis finds 39 origins within 20 kb of TELs, of which six (15%) are Rif1-unregulated, zero (0%) are Rif1-activated, and 33 (85%) are Rif1-repressed (Fig. 4D). Thus, subtelomeric regions contain a predominance of Rif1-repressed origins and are devoid of Rif1-activated origins. Although the effect of CENs and TELs on replication origins appear to be limited to a distance of ∼20 kb [8], [12], [13], [32], [33], we wondered whether there might be a distance effect along chromosome arms relative the centromeres. To address this question we determined the average distance of origins in each of the Rif1 regulation classes after excluding pericentric and subtelomeric origins, which would skew these results. Interestingly, the results show that on average, Rif1-repressed origins (349 kb, n = 141) lie significantly (p<0.001, two-sided t-tests) more distal from CENs than Rif1-activated (230 kb, n = 55) and Rif1-unregulated origins (212 kb, n = 116). This finding suggests that linear chromosomal distance from CENs favors origin repression by Rif1.

We wished to determine how chromatin binding of Rif1 relates to its function in origin regulation throughout the genome, particularly at internal chromosomal loci. Rif1 binds telomeric and subtelomeric chromatin through interaction with the DNA binding protein Rap1 [22]. However, Rif1 also binds internal chromosomal loci, most of which do not appear to bind Rap1, suggesting a novel mode of Rif1 recruitment to these loci [22]. We used the available Rif1 genome-wide binding data (ChIP-chip) to compare against the Rif1-regulated origin loci identified here [22]. We used the top 10% of Rif1 binding sites (the least stringent cutoff used in the previous study), comprising 543 non-telomeric binding loci, and tested for proximity (<∼1 kb) to Rif1-regulated origins. We excluded subtelomeric origins to focus the analysis on the possibility of Rif1 recruitment to internal origin loci. The results show 35 origins (n = 353) proximal to Rif1 binding sites, which includes 14 Rif1-repressed (n = 141), 12 Rif1-unregulated (n = 131), and nine Rif1-activated (n = 81). Thus, there appears to be no specific enrichment of Rif1 binding near internal Rif1-regulated origins. Similar analysis with 601 non-telomeric Rap1 binding sites shows 20 proximal origins, of which 14 are Rif1-repressed, one is Rif1-unregulated, and five are Rif1-activated, yet none of these loci show Rif1 binding.

## Discussion

### Rif1 is a Global Regulator of Late Origins

We set out to determine the genomic landscape of Rif1’s function in regulation of replication origin timing in *S. cerevisiae*, whether palmitoylation of Rif1, and by implication peripheral nuclear localization of origins, is required for this regulation, and whether Rif1’s reported checkpoint functions are involved in origin control. Our results clearly reveal that Rif1 regulates the initiation timing of later-firing origins in subtelomeric as well as internal chromosomal loci. The regulation of internal origins suggests that Rif1 delays origin firing through a mechanism independent of telomere proximity, although the results are fully consistent with the idea that telomere length can modulate the amount of Rif1 binding and hence, the replication timing of subtelomeric origins [34]. The independence of replication timing control from *PFA4* function is further consistent with the telomere-independence of Rif1’s mechanism, and suggests strongly that Rif1’s function in timing regulation does not require Rif1 anchoring to the nuclear envelope.

Whereas the major effect of *RIF1* deletion is to advance timing of later-firing origins, deletion of *RIF1* also results in slightly lower HU efficiency of some early origins (Rif1-activated), suggesting a slight delay in their initiation timing. For example, the majority of pericentric origins fall into this group. The much greater relative magnitude of change in Rif1-repressed origins than Rif1-activated origins as a result of *RIF1* deletion suggests that Rif1 plays a direct role to delay origin activation while the effect on early origins is likely an indirect consequence of more origins competing in G1 or early S-phase for limiting factors. In *S. pombe*, deletion of *rif1^+^* strongly reduced the HU efficiency of pericentric origins, consistent with an indirect effect due to titration of replication factors [15]. The relatively minor effect on pericentric origin firing in *S. cerevisiae* likely reflects the efficiency of the dedicated mechanism of DDK recruitment by the kinetochore complex to promote early CEN replication [13], whereas *S. pombe* uses a different mechanism to recruit DDK and promote early firing of pericentric origins [35], [36], which is insufficient in the absence of *rif1^+^*.

### Rif1 as a Checkpoint Regulator

Our results show that loss of Rif1 deregulates replication timing but maintains intra-S checkpoint signaling to Rad53 and inhibits replication rate in response to MMS. In contrast, checkpoint-defective *mec1-100* and *mec1Δ* cells maintain replication timing similar to *WT* cells, while they are defective in replication inhibition in responses to DNA damage. These findings strongly suggest that Rif1 regulates origin timing independently of the intra-S checkpoint pathway. Similar conclusions were drawn regarding Rif1 in *S. pombe* [15]. In addition, recent reports that Rif1 regulates origin timing by recruiting a phosphatase that opposes DDK-dependent phosphorylation of Mcm4 are also consistent with our conclusion [37]–[39]. Rif1 has been characterized as an anti-checkpoint factor in the DNA damage response to uncapped telomeres in *S. cerevisiae*, which appears to involve suppression of checkpoint signaling from single-stranded DNA (ssDNA) [40], [41]. It is unclear whether origin regulation by Rif1 has any connection to its role in the uncapped telomere response; however, it is feasible that Rif1’s function in temporally distributing initiation events might reduce the total amount of ssDNA contributing to a checkpoint-signal threshold. Consistent with a primordial role of Rif1 in DNA damage sensing in yeast, mammalian Rif1 has clearly evolved a critical function in DNA damage signaling and processing in addition to its role in replication timing control [42].

### How does Rif1 Act at Internal Chromosomal Loci?

An important question that remains is whether and how Rif1 is recruited independently of Rap1 or Taz1 to chromatin to regulate internal origin firing. In *S. pombe*, origins delayed by Rif1 are found more proximal to Rif1 binding sites than other origins suggesting a direct, or at least localized, effect [15]. We did not detect a similar relationship in budding yeast; however, this may reflect limitations of the data due to experimental differences. The available budding yeast Rif1 ChIP-chip data are from unsynchronized cells, whereas the *S. pombe* data show peak binding of Rif1 to internal chromosomal loci in cells at G1/S [15]. Rif1 has also been suggested to act in higher-level chromosome organization, potentially drawing distal DNA sequences together [14]–[16]. Thus, the role of Rif1 in origin regulation may not require direct binding to an origin in *cis* to regulate its function. Future studies should examine the cell cycle binding of Rif1 throughout the genome, as well as its role in three-dimensional genome organization.

## Materials and Methods

### Yeast Strains and Methods

All strains are related to CVy63, which is W303-derived, *MAT* ***a*** *ade2-1 ura3-1 his3-11,15 trp1-1 can1-100 bar1Δ::hisG LEU2::BrdU-Inc* [43]. TRy1 (*rif1Δ)* and TRy3 (*pfa4Δ*) were constructed by deletion of *RIF1* and *PFA4*, respectively, in CVy63 by long oligonucleotide-based replacement and selection for KanMx. YZy52 (*mec1-100*) is congenic with the above strains and has been described previously [44]. OAy1050 (*sml1Δ rif1Δ)* is a haploid segregant derived from a cross of TRy1 and SSy164 (*Mat* ***α*** *sml1Δ::HIS3*), and OAy1056 (*sml1Δ*) and OAy1059 (*sml1Δ mec1Δ*) are haploid segregants derived from a cross of DGy159 [45] and CVy70 (*Mat* ***α*** *URA3::BrdU-Inc* ***)***, respectively. For G1-phase block-and-release, log-phase cell cultures were resuspended in fresh YEPD at O.D. 0.5, and incubated with 7.5 nM α-factor at 23°C for 4 hrs. Arrested cultures were released from α-factor arrest by resuspension in fresh YEPD at O.D. 1.0 with 200 µg/mL Pronase E (Sigma-Aldrich, P5147) and gentle sonication to disperse cells. BrdU (Sigma-Aldrich, B5002) was used at 400 µg/mL. For early S-phase analysis of replication, cells were released into the presence of 0.2 M HU (Sigma-Aldrich, H8627) 45 min at 23°C. MMS (Sigma-Aldrich, 129925) was added to 0.033% to cells released from α-factor arrest at 30°C. DNA content analysis was perfomed with SYTOX Green (Molecular Probes, S7020) as described previously [44]. Analysis of Rad53 by immunoblotting was performed with anti-Rad53 antibody (SC6749; Santa Cruz Biotech.) at 1∶1000 using previously described conditions [45].

### BrdU-IP-chip

Genomic DNA was isolated from 25 mL BrdU-labeled cultures, 1 µg total genomic DNA was immunoprecipitated, and half of the immunoprecipitated DNA was subjected to whole genome amplification (Sigma-Aldrich, WGA2), labeling, and hybridization as previously described [43]. Samples were hybridized to custom-designed DNA oligonucleotide tiling arrays (Roche-Nimblegen) containing 135,000 probes, with one ∼60 mer probe for every ∼80 bp of unique genomic sequence. Array data from two experimental replicates were processed as previously described [12], [46]. A final.txt file containing averaged data from the two experimental replicates was used for generating plots and T_Rep_ quartile analysis.

### BrdU-IP-Seq

Genomic DNA was isolated from 50 mL BrdU-labeled cultures, 5 µg total genomic DNA was immunoprecipitated as described [12], and the entire immunoprecipitate was amplified by Illumina protocols with inclusion of barcodes and indexes to allow pooling of samples [47]. Sequencing (50 bp paired-end) was carried out on the Illumina Hi-Seq platform by the USC Epigenome Center.

### Analysis of Sequencing Data

Barcodes were split using the barcode splitter from the FAST-X toolkit [48]. Sequence libraries were aligned to *S. cerevisiae* genome release r.64 using Bowtie2 [49]. The first 10 bp were trimmed from the 5′ end to account for the barcode and allow for proper alignment. Aligned sequences were sorted and binned into 50 bp non-overlapping bins [50], [51], median-smoothed over a 1 kb window and quantile-normalized between replicates. Two experimental replicates were averaged and smoothed again. BrdU peaks were called using MACS (p<0.01) [52]. Called peaks were then cross-referenced against origins defined in OriDB [53] as “confirmed” or “likely” to eliminate any peaks not aligning with an origin. Origin peaks were subjected to DiffBind analysis (FDR <0.05) for calling of differential peak sizes [54]. A final.txt file containing averaged data from the two experimental replicates was used for generating plots and for further analysis.

### Analysis of Origin in Relation to Genomic Features and Other Datasets

Origin T_rep_ was taken from OriDB; T_rep_ data for the 392 origins called in this study were divided into four quartiles based on their T_rep_ rank, and the average signal from the.bed file was aligned on the midpoint of the origin (ARS) sequence as defined in OriDB. Data were plotted for a 40 kb (Fig. 2C, D) or 5 kb (Fig. 4B) window around the origins for each quartile. For analysis of intersection of origins with other features, a 1 kb window was centered on the midpoint of the origin sequences as defined in OriDB. For analysis of overlap with origins, Rif1 binding sites were assigned coordinates corresponding to the feature they were associated with in the published dataset [22], as follows: for ORFs, the whole ORF coordinates were used; for intergenics, 1 kb of the intergenic sequence nearest to the gene for which the intergenic is named was used. Any overlap (≥1 bp) between these defined windows was determined using Bedtools intersect [51]. The average distance from CENs of all non-telomeric and non-pericentric origins was determined by using Bedtools closest function [51].

### Data Accession

Data files have been deposited at GEO, accession number: GSE55156.

## Supporting Information

Figure S1 **Analysis of early S-phase by BrdU-IP-chip for all chromosomes.** Plots show BrdU incorporation in HU-arrested cells. Data from a single replicate is shown. Plot colors are keyed above. Data for the second experimental replicate is available at GEO.
(ZIP)

Figure S2 **DNA content analysis of cells released into S-phase for the temporal analysis of replication in** **Fig. 2** **.**
(PDF)

Figure S3 **Temporal analysis of replication by BrdU-IP-chip for all chromosomes.** Plots show average BrdU incorporation from duplicate experiments. Plot colors are keyed above. The lack of signal at origins (*ARS305*, *ARS608*, and *ARS609*, on chromosomes III and VI) in the *mec1-100* strain is due to deletion of these origins, which was done for the purposes of a previous study, but is inconsequential for the current study.
(ZIP)

Figure S4 **Temporal analysis of replication by BrdU-IP-chip for all chromosomes.** Plots show average BrdU incorporation from duplicate experiments. Plot colors are keyed above.
(ZIP)

Figure S5 **Analysis of early S-phase by BrdU-IP-Seq for all chromosomes.** Plots show average BrdU incorporation from duplicate HU experiments. Origin classes are color-coded below each plot. Plot colors are keyed above.
(ZIP)

Table S1 **List of origins classified by Rif1 regulation.** Columns are labeled at top. Class refers to regulation by Rif1.
(TXT)
