# Supplementary figures and images for: Rif1 Regulates Initiation Timing of Late Replication Origins throughout the S. cerevisiae Genome
Source: PLoS One. 2014 May 30;9(5):e98501. doi: 10.1371/journal.pone.0098501 (PMC4039536; doi:10.1371/journal.pone.0098501)

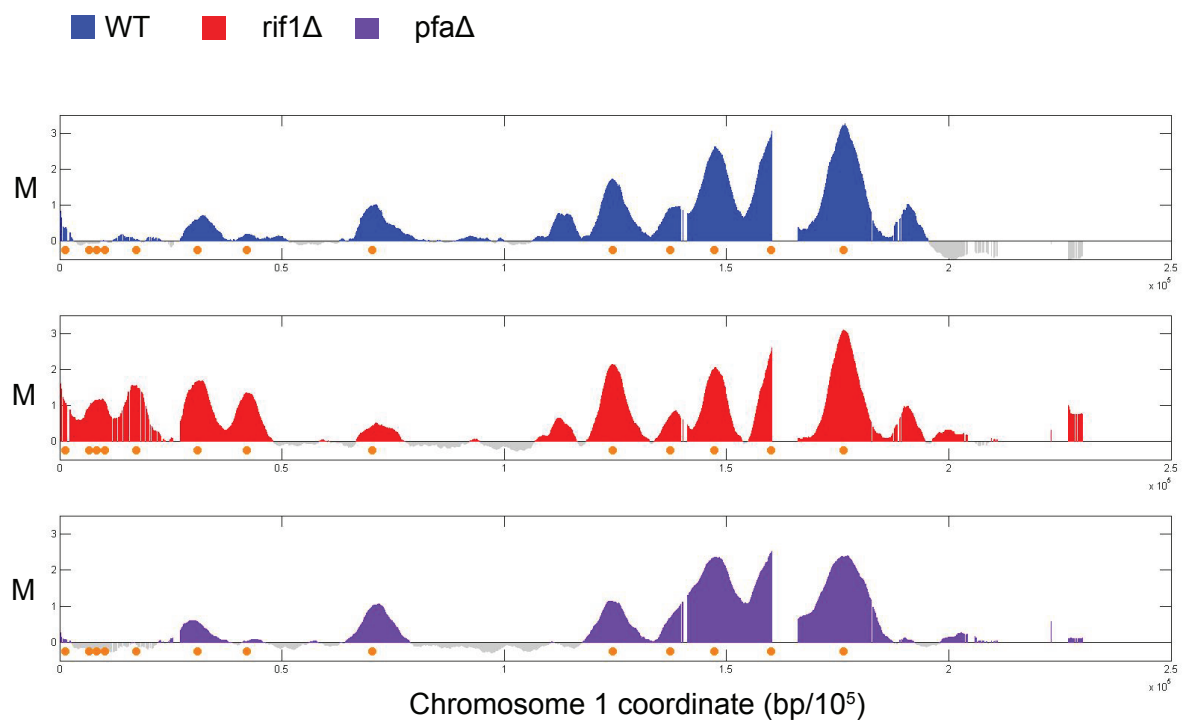

Supplement: Figure S1 — Analysis of early S-phase by BrdU-IP-chip for all chromosomes. Plots show BrdU incorporation in HU-arrested cells. Data from a single replicate is shown. Plot colors are keyed above. Data for the second experimental replicate is available at GEO. (ZIP) [file pone.0098501.s001.zip › FigS1/S1.1.pdf]

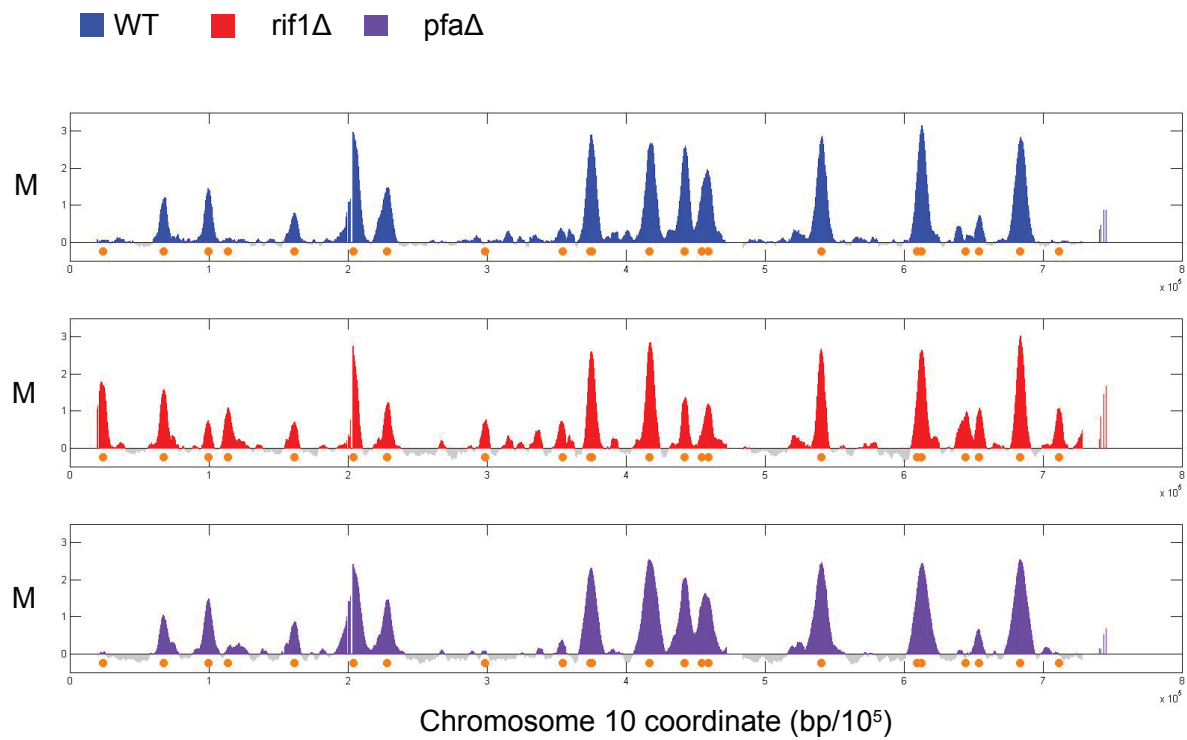

Supplement: Figure S1 — Analysis of early S-phase by BrdU-IP-chip for all chromosomes. Plots show BrdU incorporation in HU-arrested cells. Data from a single replicate is shown. Plot colors are keyed above. Data for the second experimental replicate is available at GEO. (ZIP) [file pone.0098501.s001.zip › FigS1/S1.10.pdf]

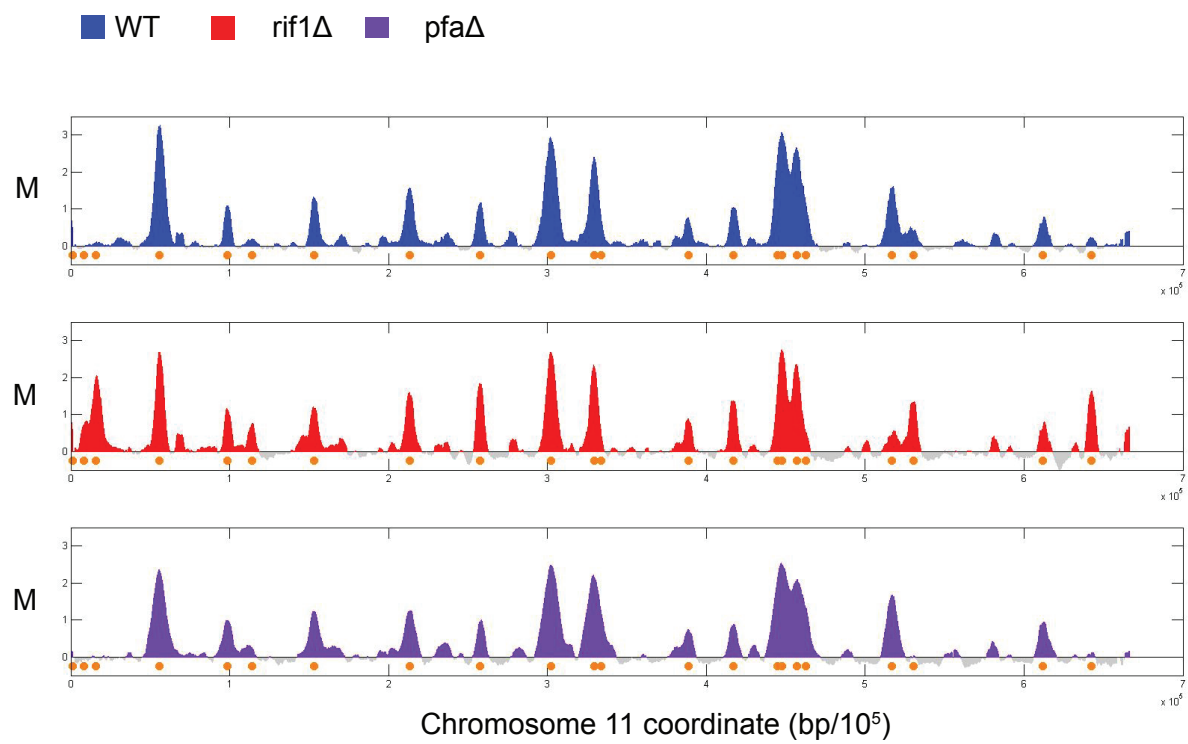

Supplement: Figure S1 — Analysis of early S-phase by BrdU-IP-chip for all chromosomes. Plots show BrdU incorporation in HU-arrested cells. Data from a single replicate is shown. Plot colors are keyed above. Data for the second experimental replicate is available at GEO. (ZIP) [file pone.0098501.s001.zip › FigS1/S1.11.pdf]

■ WT   ■ rif1Δ   ■ pfaΔ

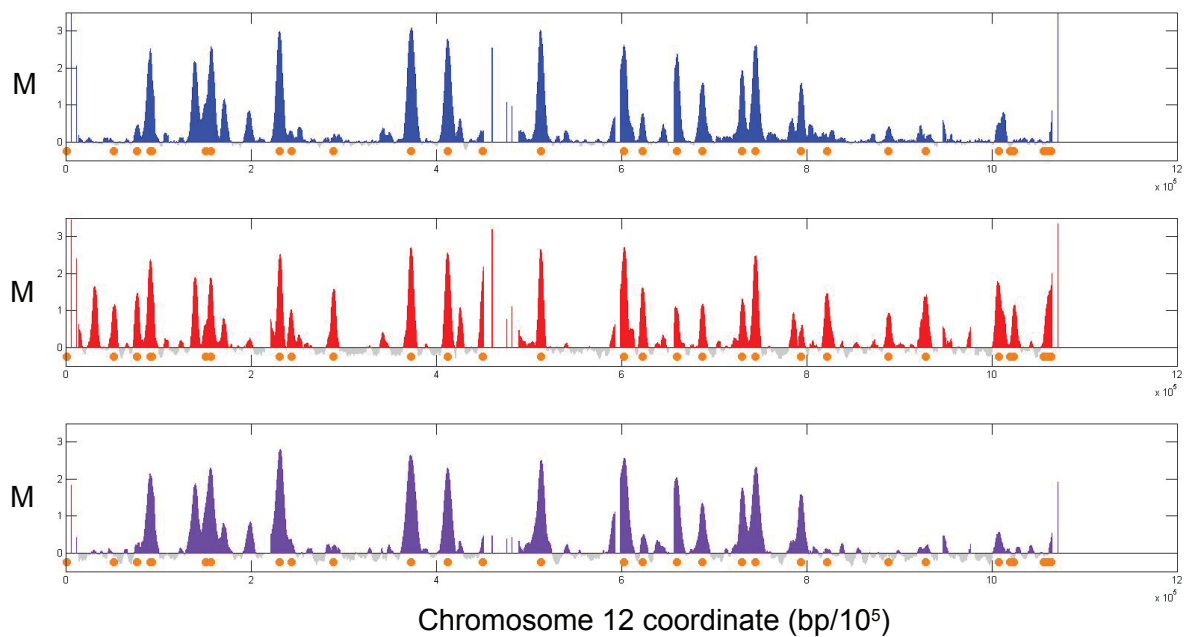

Supplement: Figure S1 — Analysis of early S-phase by BrdU-IP-chip for all chromosomes. Plots show BrdU incorporation in HU-arrested cells. Data from a single replicate is shown. Plot colors are keyed above. Data for the second experimental replicate is available at GEO. (ZIP) [file pone.0098501.s001.zip › FigS1/S1.12.pdf]

■ WT   ■ rif1Δ   ■ pfaΔ

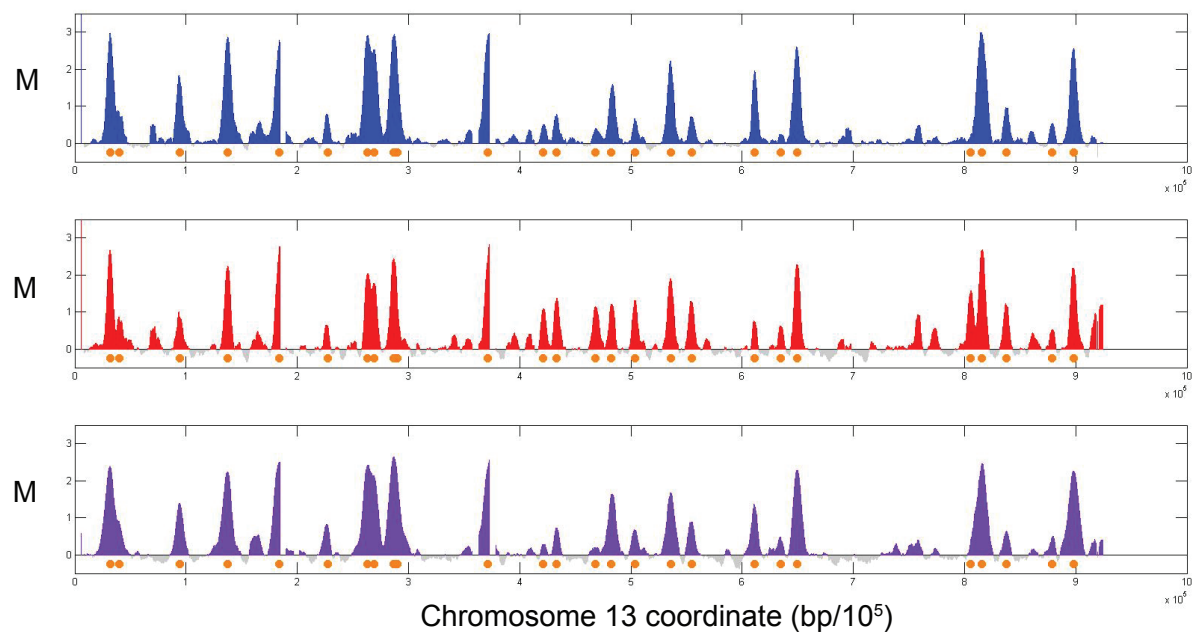

Supplement: Figure S1 — Analysis of early S-phase by BrdU-IP-chip for all chromosomes. Plots show BrdU incorporation in HU-arrested cells. Data from a single replicate is shown. Plot colors are keyed above. Data for the second experimental replicate is available at GEO. (ZIP) [file pone.0098501.s001.zip › FigS1/S1.13.pdf]

■ WT   ■ rif1Δ   ■ pfaΔ

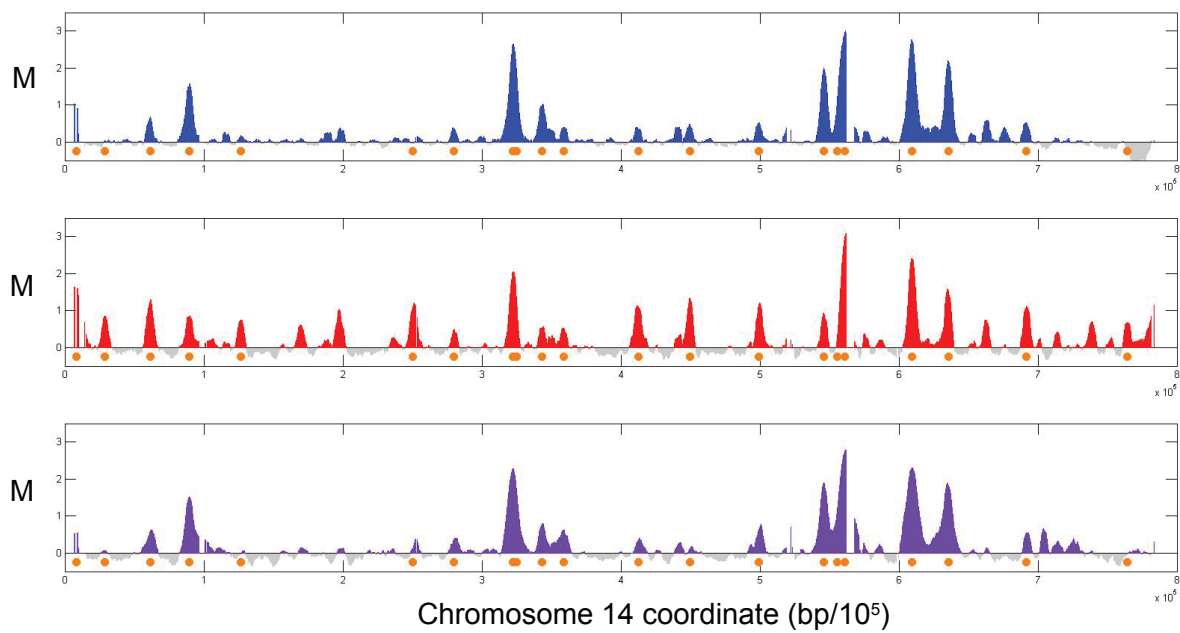

Supplement: Figure S1 — Analysis of early S-phase by BrdU-IP-chip for all chromosomes. Plots show BrdU incorporation in HU-arrested cells. Data from a single replicate is shown. Plot colors are keyed above. Data for the second experimental replicate is available at GEO. (ZIP) [file pone.0098501.s001.zip › FigS1/S1.14.pdf]

■ WT   ■ *rif1Δ*   ■ *pfaΔ*

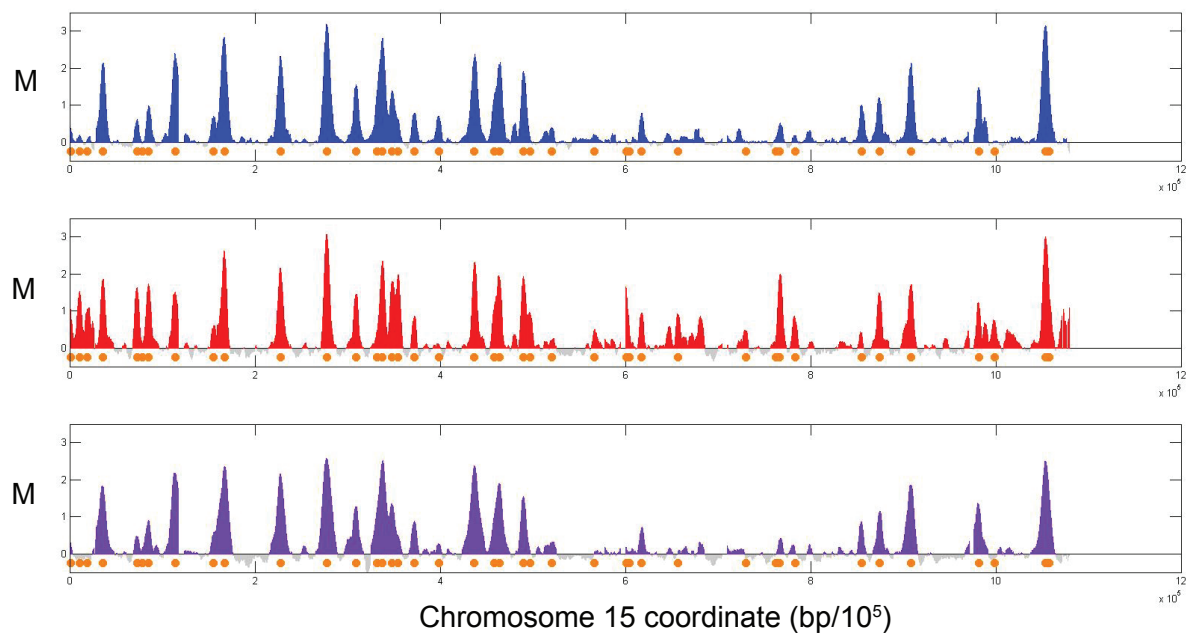

Supplement: Figure S1 — Analysis of early S-phase by BrdU-IP-chip for all chromosomes. Plots show BrdU incorporation in HU-arrested cells. Data from a single replicate is shown. Plot colors are keyed above. Data for the second experimental replicate is available at GEO. (ZIP) [file pone.0098501.s001.zip › FigS1/S1.15.pdf]

■ WT   ■ *rif1*Δ   ■ *pfa*Δ

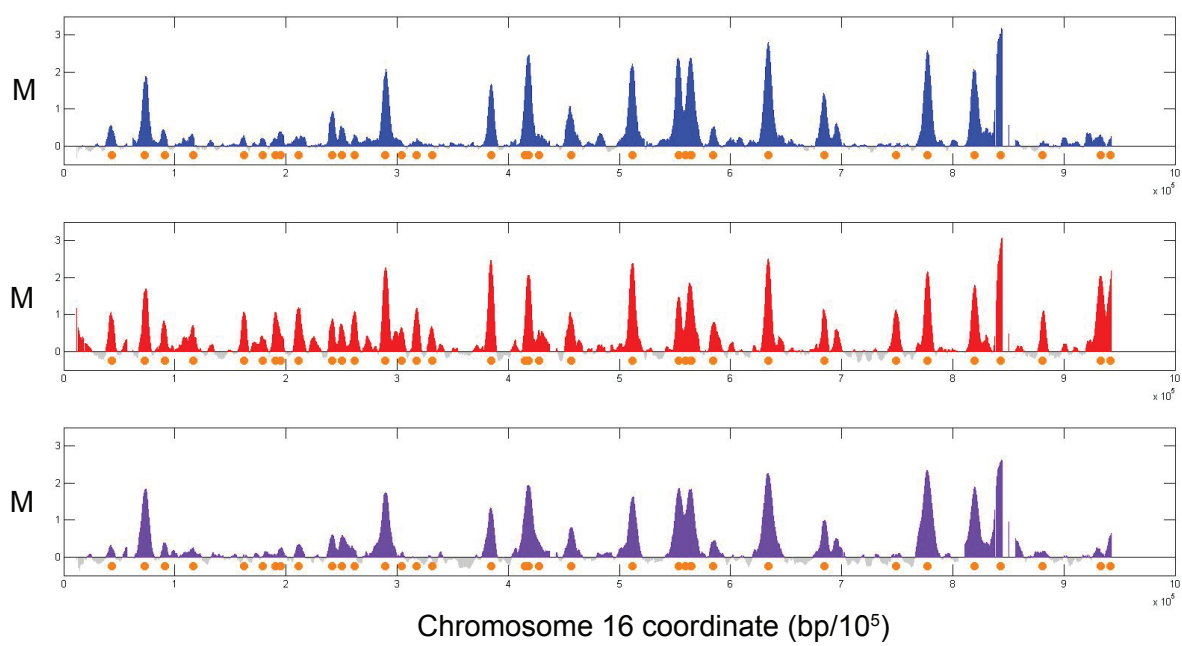

Supplement: Figure S1 — Analysis of early S-phase by BrdU-IP-chip for all chromosomes. Plots show BrdU incorporation in HU-arrested cells. Data from a single replicate is shown. Plot colors are keyed above. Data for the second experimental replicate is available at GEO. (ZIP) [file pone.0098501.s001.zip › FigS1/S1.16.pdf]

■ WT   ■ *rif1*Δ   ■ *pfa*Δ

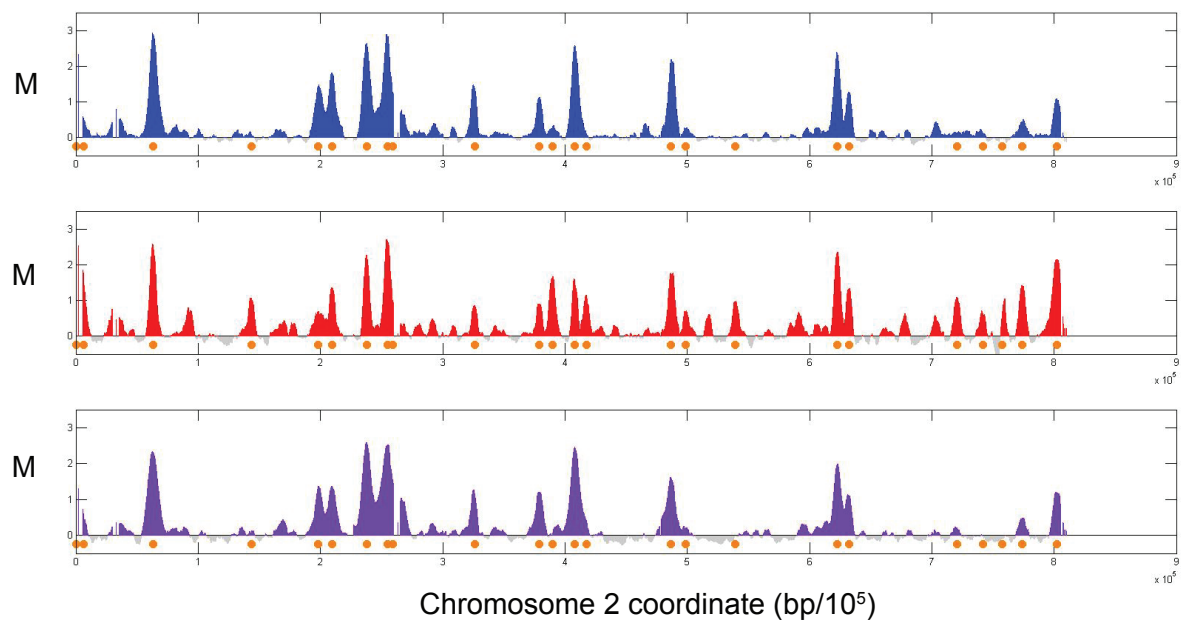

Supplement: Figure S1 — Analysis of early S-phase by BrdU-IP-chip for all chromosomes. Plots show BrdU incorporation in HU-arrested cells. Data from a single replicate is shown. Plot colors are keyed above. Data for the second experimental replicate is available at GEO. (ZIP) [file pone.0098501.s001.zip › FigS1/S1.2.pdf]

■ WT   ■ *rif1Δ*   ■ *pfaΔ*

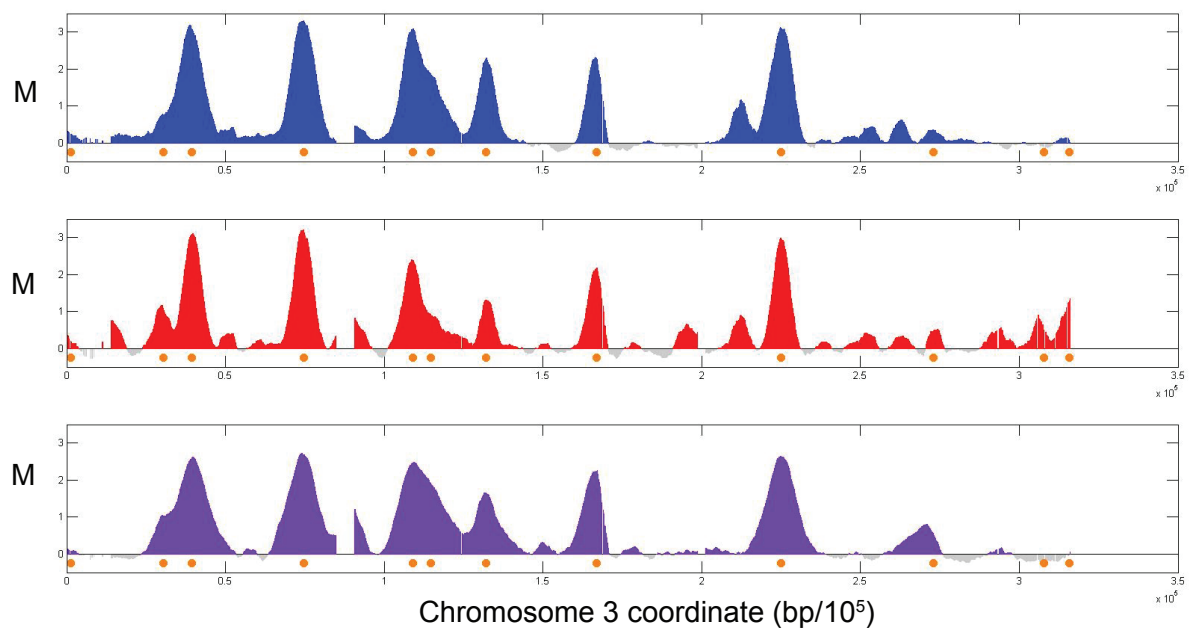

Supplement: Figure S1 — Analysis of early S-phase by BrdU-IP-chip for all chromosomes. Plots show BrdU incorporation in HU-arrested cells. Data from a single replicate is shown. Plot colors are keyed above. Data for the second experimental replicate is available at GEO. (ZIP) [file pone.0098501.s001.zip › FigS1/S1.3.pdf]

■ WT   ■ rif1Δ   ■ pfaΔ

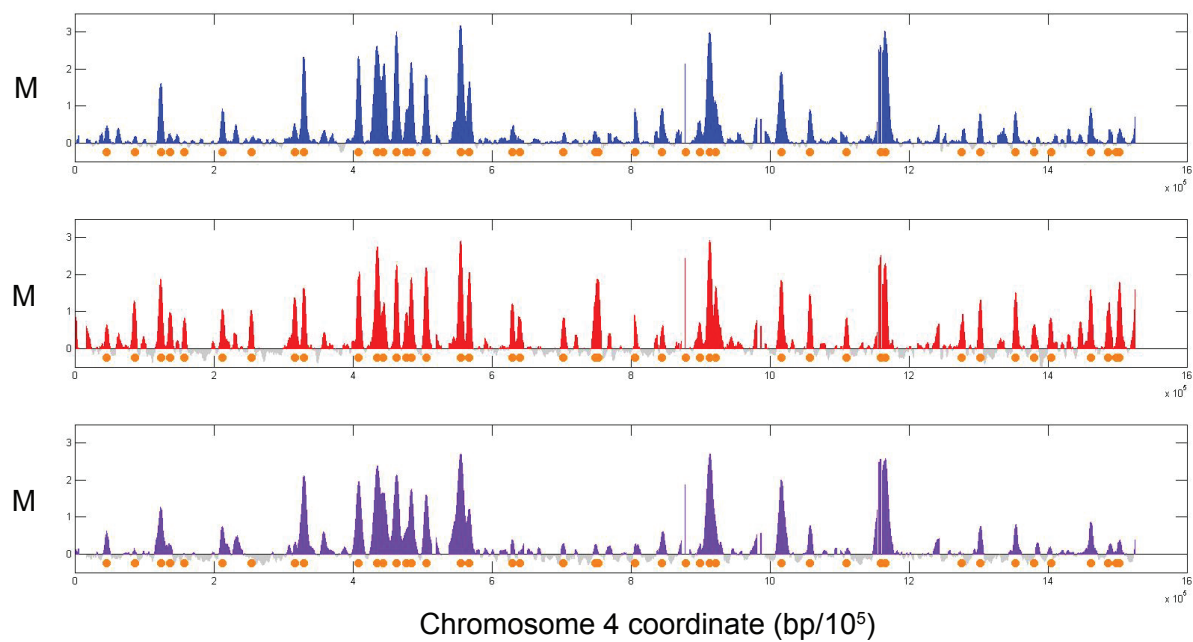

Supplement: Figure S1 — Analysis of early S-phase by BrdU-IP-chip for all chromosomes. Plots show BrdU incorporation in HU-arrested cells. Data from a single replicate is shown. Plot colors are keyed above. Data for the second experimental replicate is available at GEO. (ZIP) [file pone.0098501.s001.zip › FigS1/S1.4.pdf]

■ WT   ■ rif1Δ   ■ pfaΔ

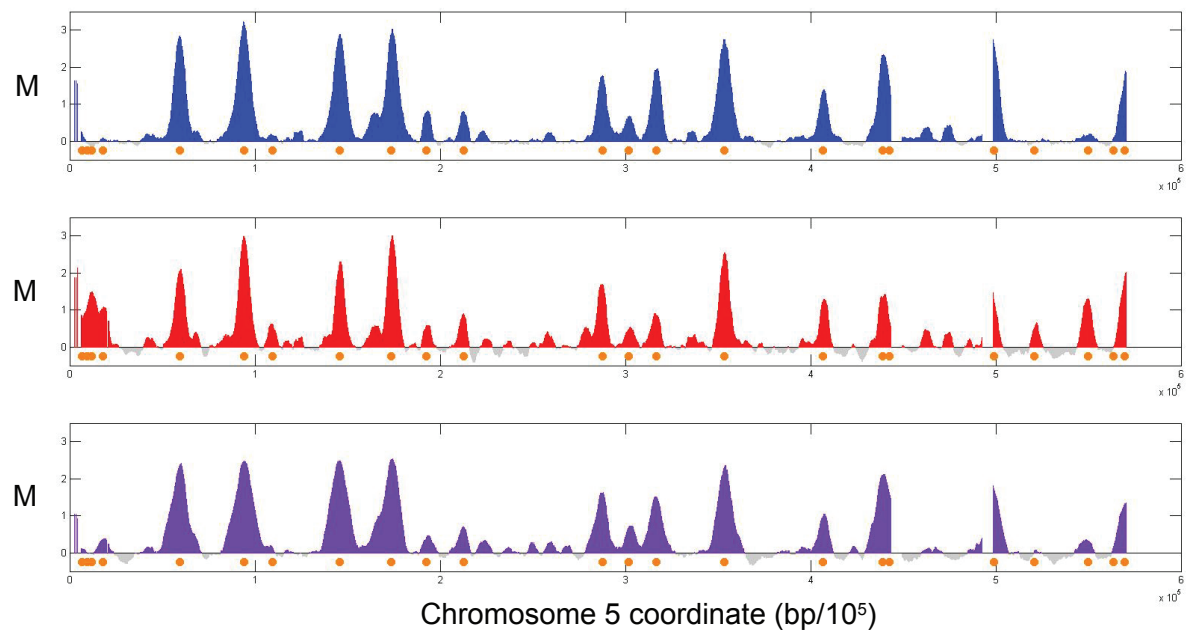

Supplement: Figure S1 — Analysis of early S-phase by BrdU-IP-chip for all chromosomes. Plots show BrdU incorporation in HU-arrested cells. Data from a single replicate is shown. Plot colors are keyed above. Data for the second experimental replicate is available at GEO. (ZIP) [file pone.0098501.s001.zip › FigS1/S1.5.pdf]

■ WT   ■ rif1Δ   ■ pfaΔ

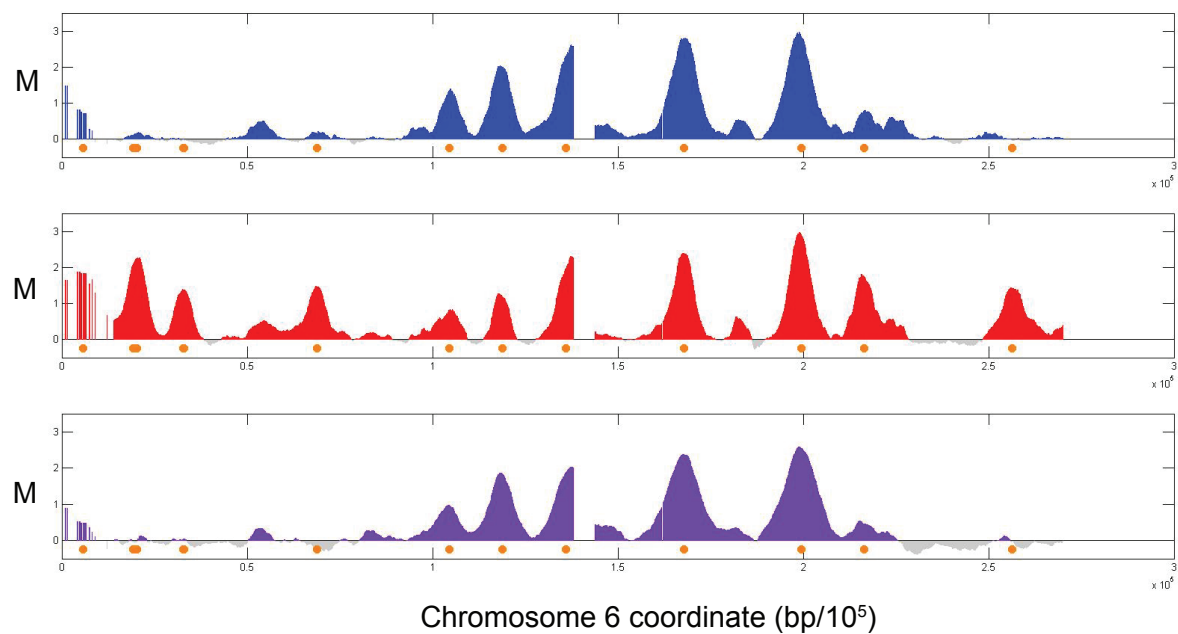

Supplement: Figure S1 — Analysis of early S-phase by BrdU-IP-chip for all chromosomes. Plots show BrdU incorporation in HU-arrested cells. Data from a single replicate is shown. Plot colors are keyed above. Data for the second experimental replicate is available at GEO. (ZIP) [file pone.0098501.s001.zip › FigS1/S1.6.pdf]

■ WT   ■ rif1Δ   ■ pfaΔ

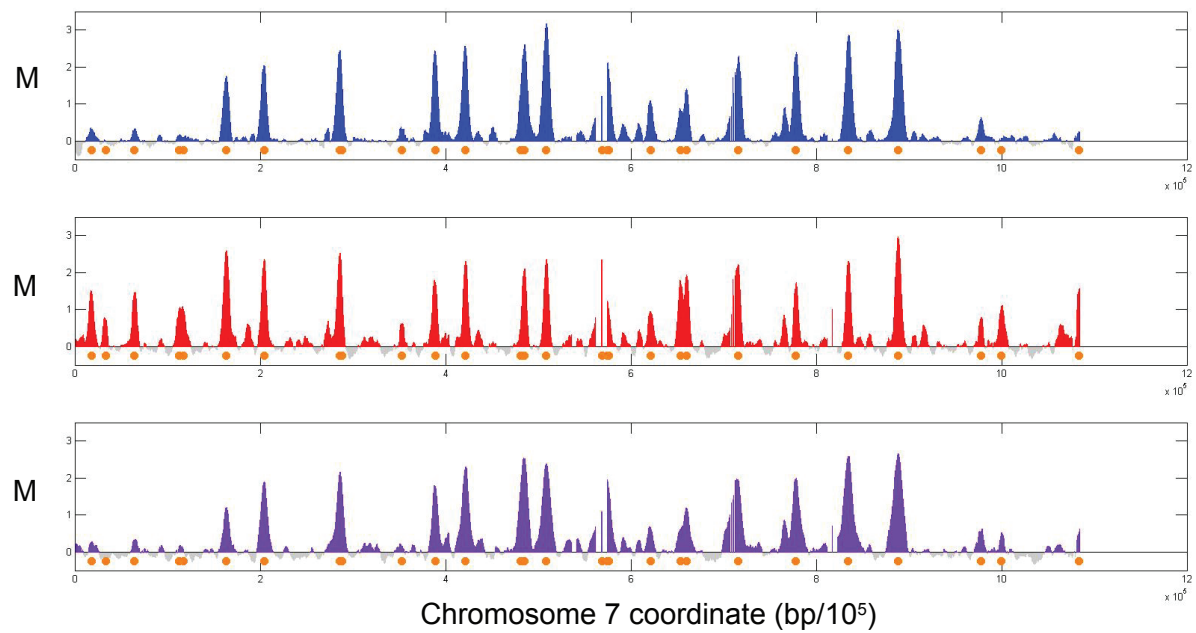

Supplement: Figure S1 — Analysis of early S-phase by BrdU-IP-chip for all chromosomes. Plots show BrdU incorporation in HU-arrested cells. Data from a single replicate is shown. Plot colors are keyed above. Data for the second experimental replicate is available at GEO. (ZIP) [file pone.0098501.s001.zip › FigS1/S1.7.pdf]

■ WT   ■ *rif1Δ*   ■ *pfaΔ*

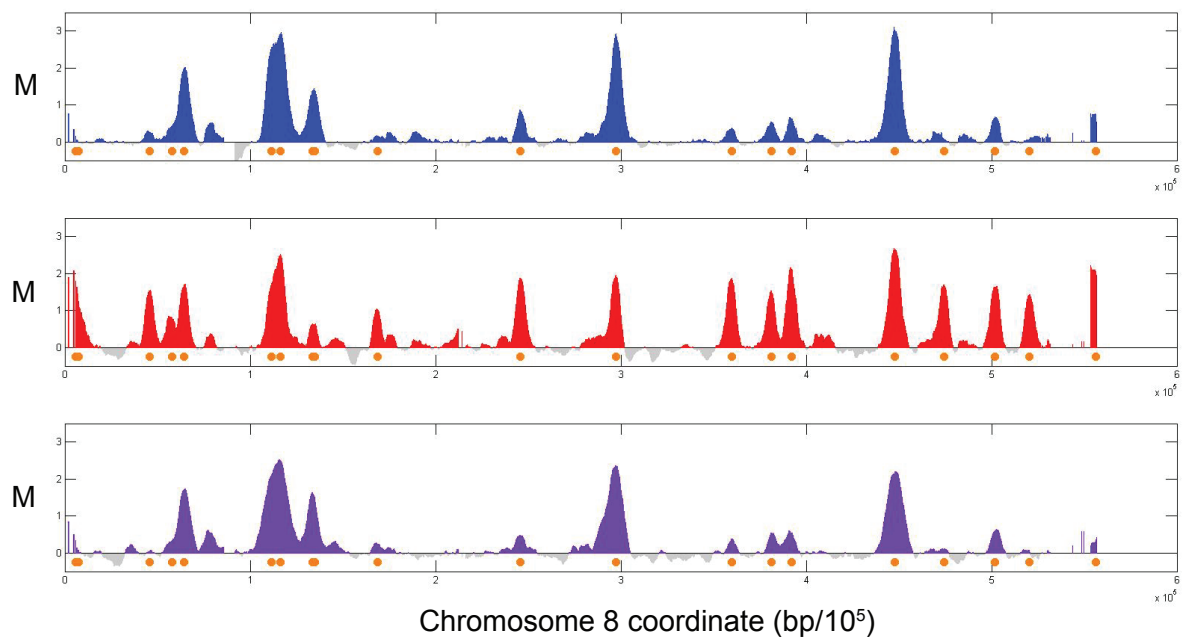

Supplement: Figure S1 — Analysis of early S-phase by BrdU-IP-chip for all chromosomes. Plots show BrdU incorporation in HU-arrested cells. Data from a single replicate is shown. Plot colors are keyed above. Data for the second experimental replicate is available at GEO. (ZIP) [file pone.0098501.s001.zip › FigS1/S1.8.pdf]

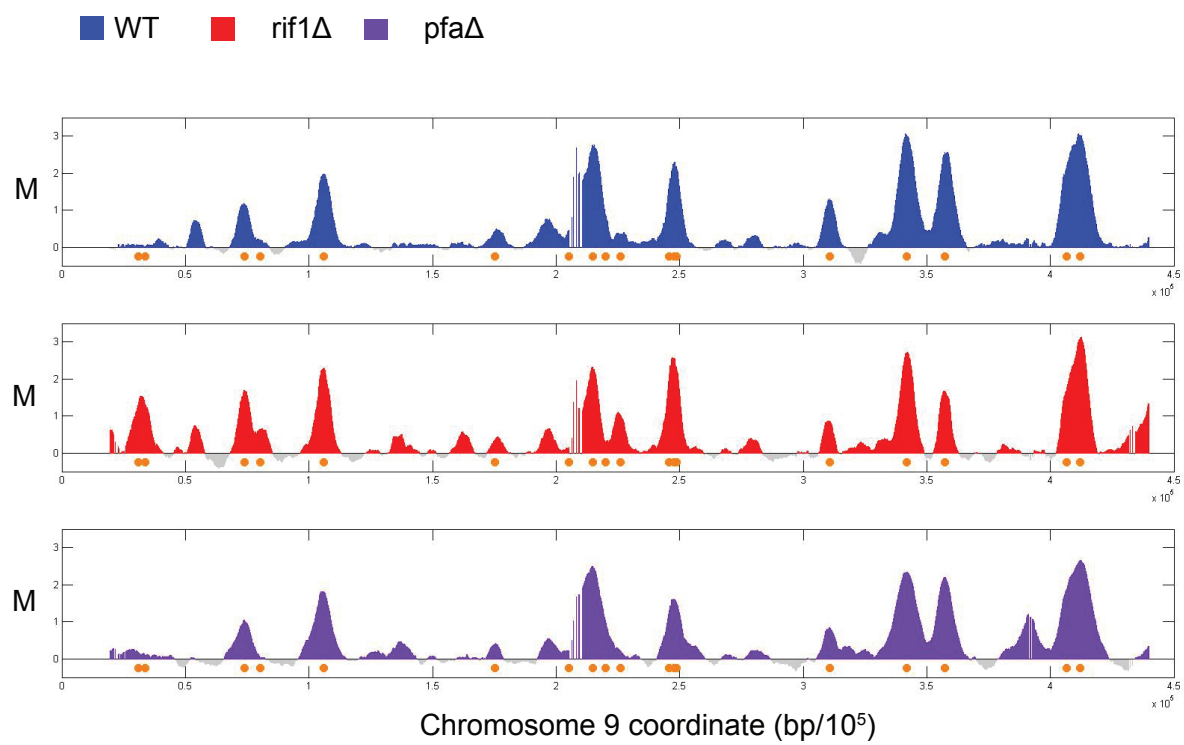

Supplement: Figure S1 — Analysis of early S-phase by BrdU-IP-chip for all chromosomes. Plots show BrdU incorporation in HU-arrested cells. Data from a single replicate is shown. Plot colors are keyed above. Data for the second experimental replicate is available at GEO. (ZIP) [file pone.0098501.s001.zip › FigS1/S1.9.pdf]

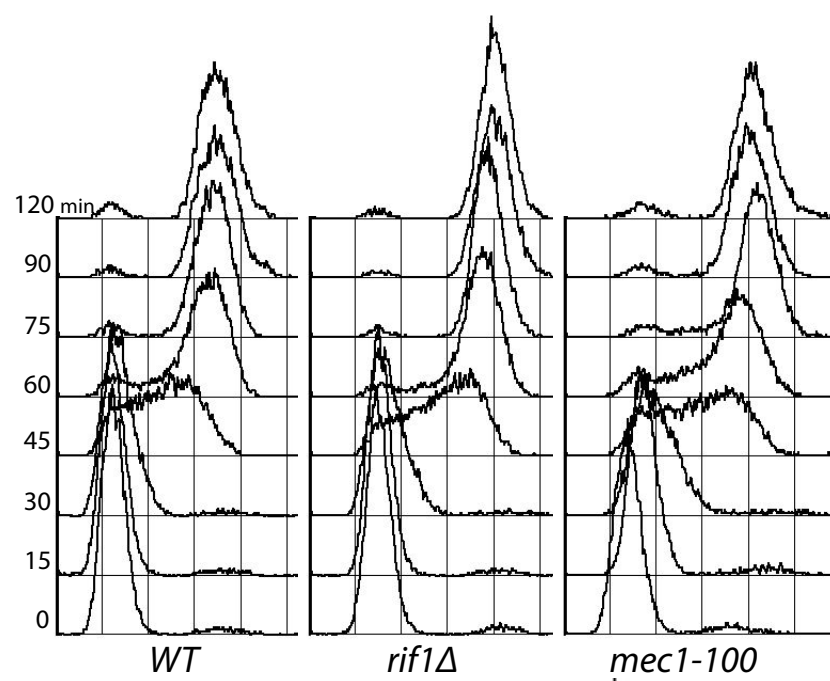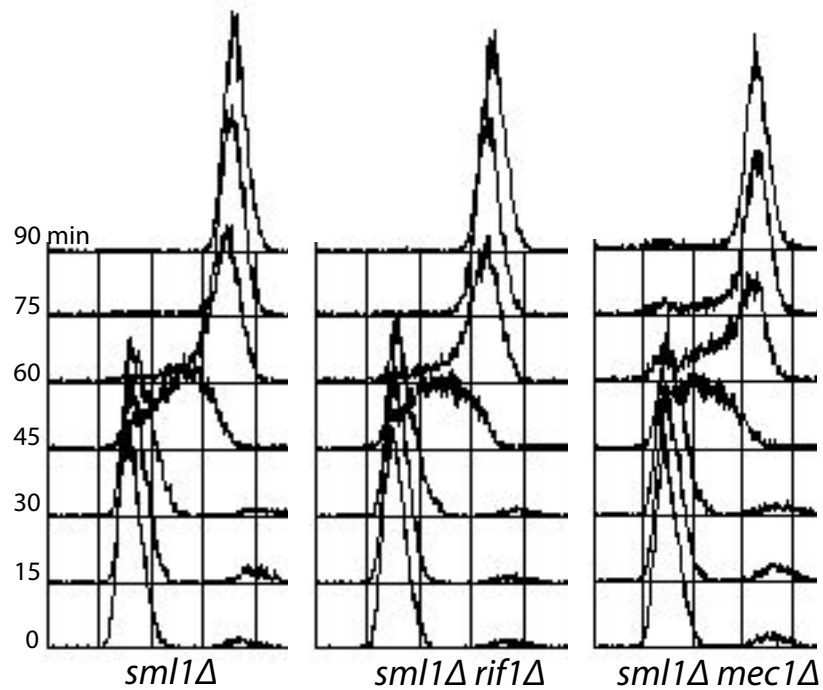

Supplement: Figure S2 — DNA content analysis of cells released into S-phase for the temporal analysis of replication in Fig. 2 . (PDF) [file pone.0098501.s002.pdf]

■ WT      ■ *rif1Δ*      ■ *mec1-100*

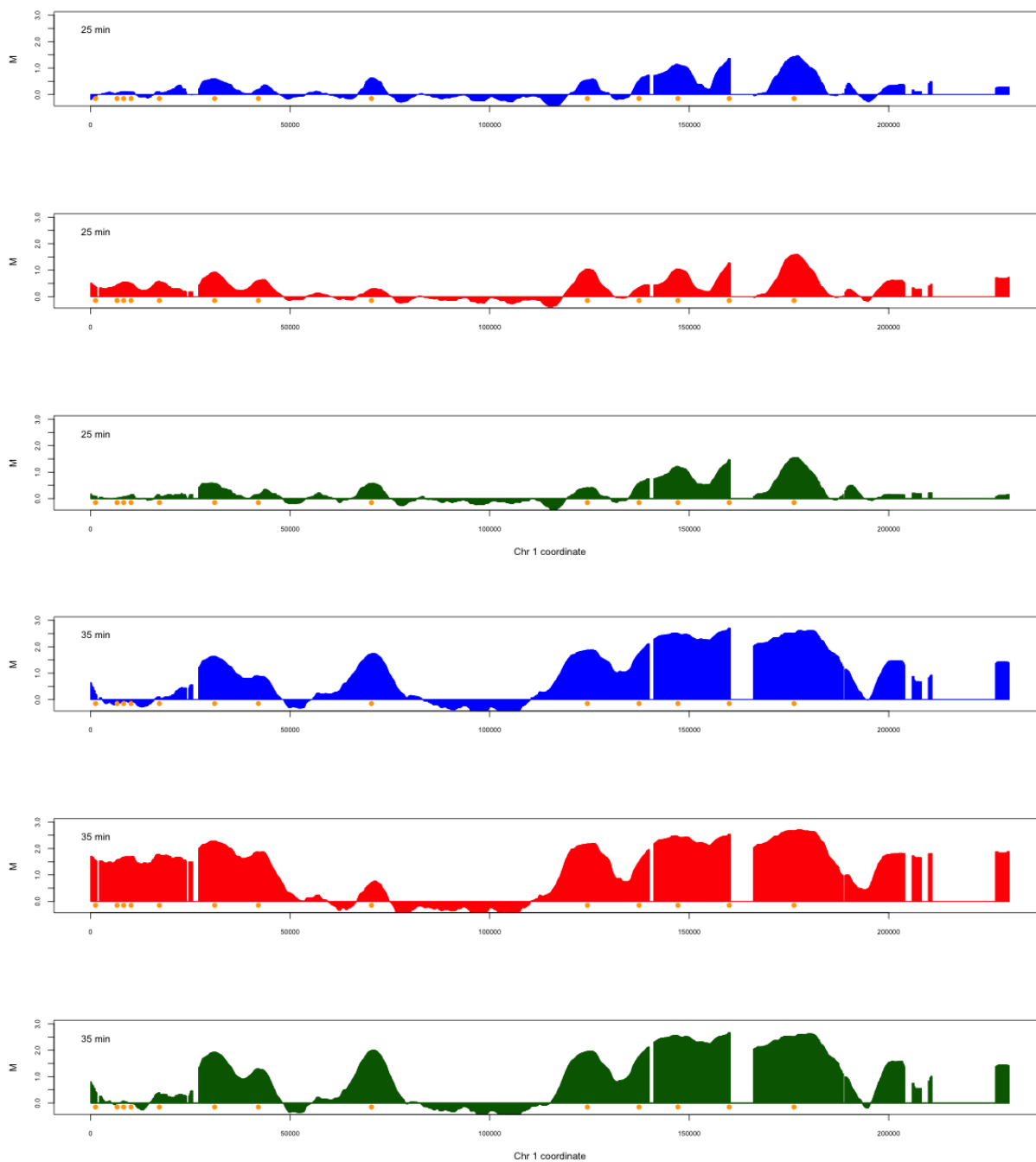

Supplement: Figure S3 — Temporal analysis of replication by BrdU-IP-chip for all chromosomes. Plots show average BrdU incorporation from duplicate experiments. Plot colors are keyed above. The lack of signal at origins (ARS305, ARS608, and ARS609, on chromosomes III and VI) in the mec1-100 strain is due to deletion of these origins, which was done for the purposes of a previous study, but is inconsequential for the current study. (ZIP) [file pone.0098501.s003.zip › FigS3/S3.1.pdf]

■ WT   ■ rif1Δ   ■ mec1-100

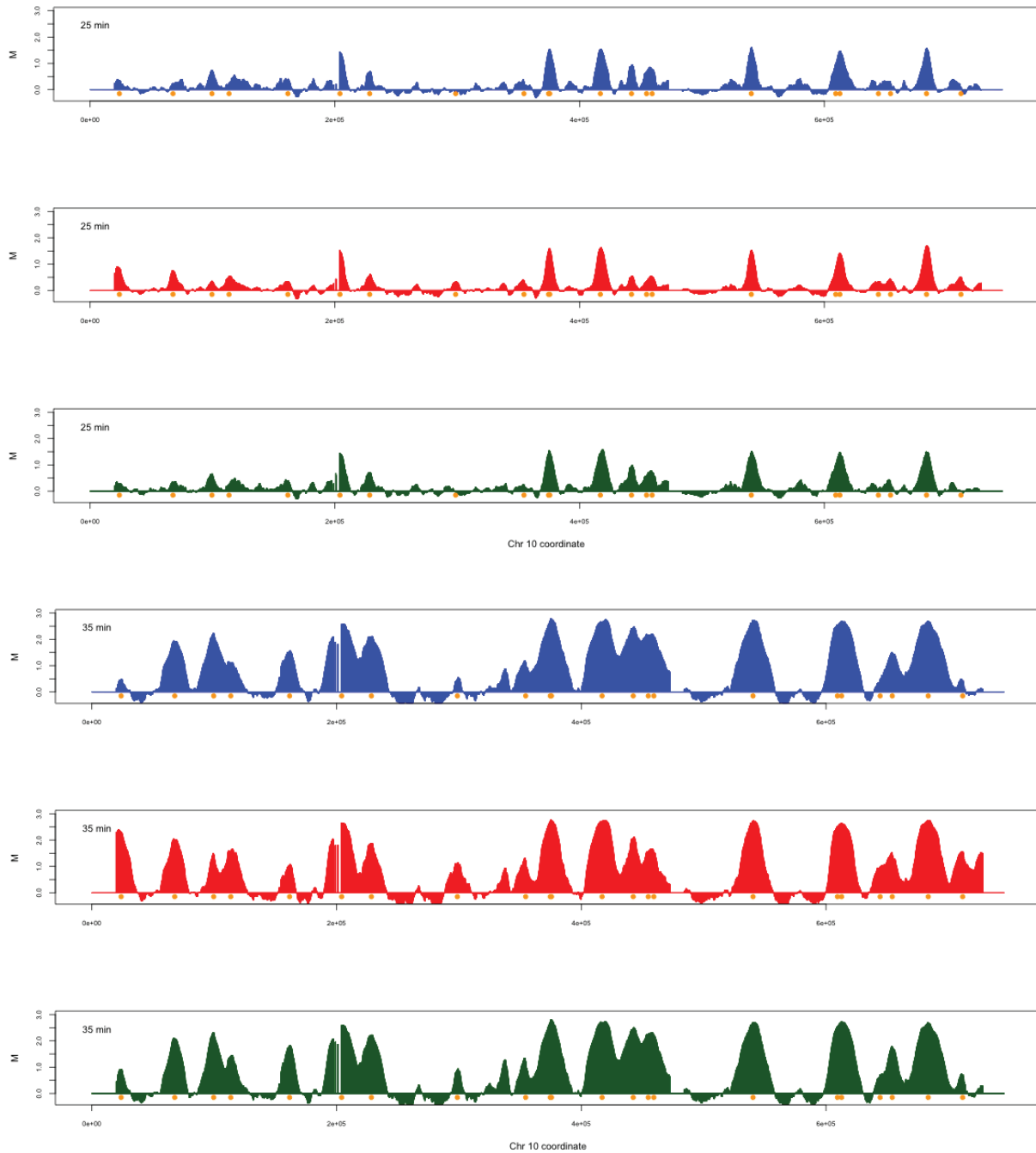

Supplement: Figure S3 — Temporal analysis of replication by BrdU-IP-chip for all chromosomes. Plots show average BrdU incorporation from duplicate experiments. Plot colors are keyed above. The lack of signal at origins (ARS305, ARS608, and ARS609, on chromosomes III and VI) in the mec1-100 strain is due to deletion of these origins, which was done for the purposes of a previous study, but is inconsequential for the current study. (ZIP) [file pone.0098501.s003.zip › FigS3/S3.10.pdf]

■ WT   ■ rif1Δ   ■ mec1-100

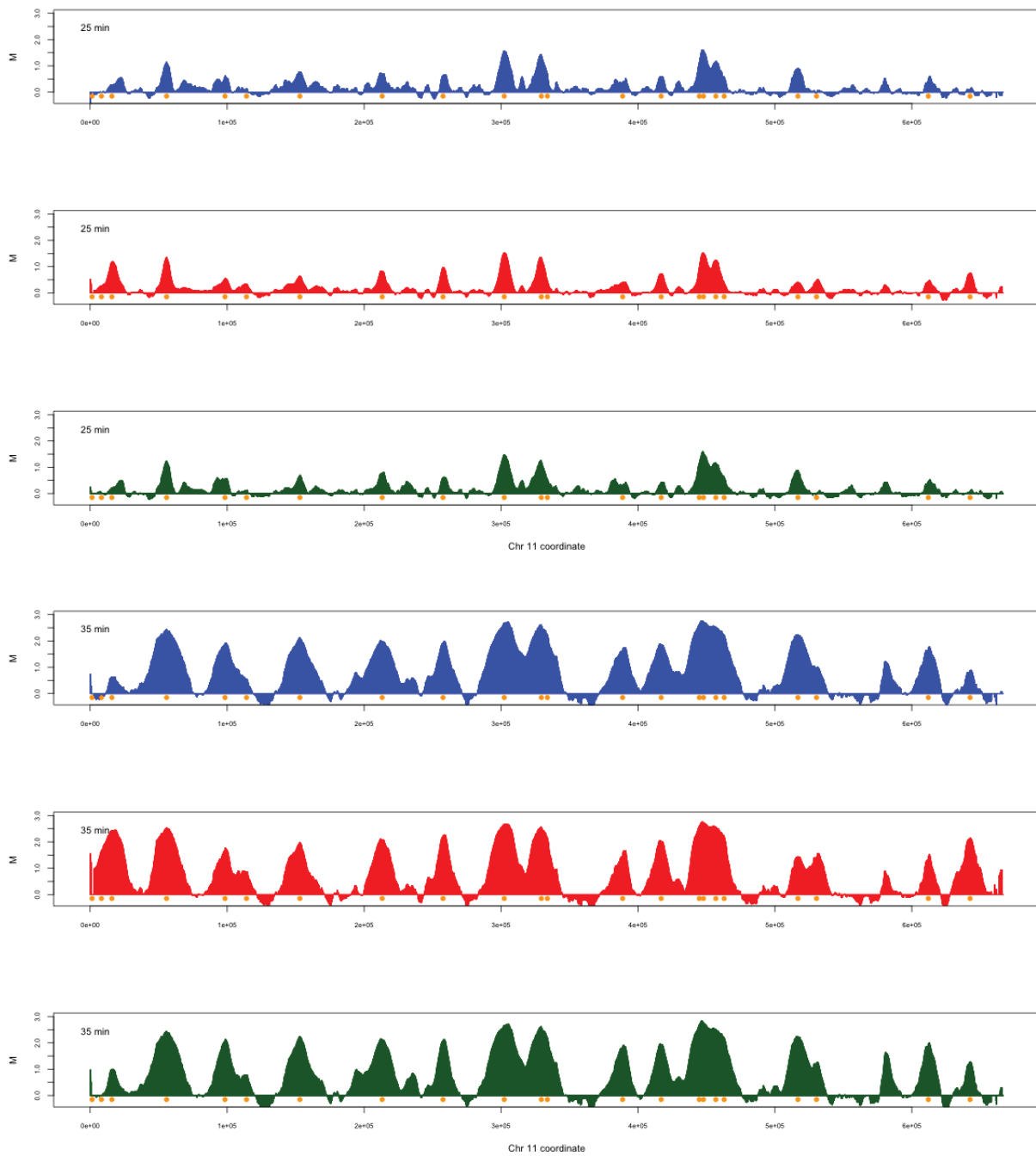

Supplement: Figure S3 — Temporal analysis of replication by BrdU-IP-chip for all chromosomes. Plots show average BrdU incorporation from duplicate experiments. Plot colors are keyed above. The lack of signal at origins (ARS305, ARS608, and ARS609, on chromosomes III and VI) in the mec1-100 strain is due to deletion of these origins, which was done for the purposes of a previous study, but is inconsequential for the current study. (ZIP) [file pone.0098501.s003.zip › FigS3/S3.11.pdf]

■ WT   ■ rif1Δ   ■ mec1-100

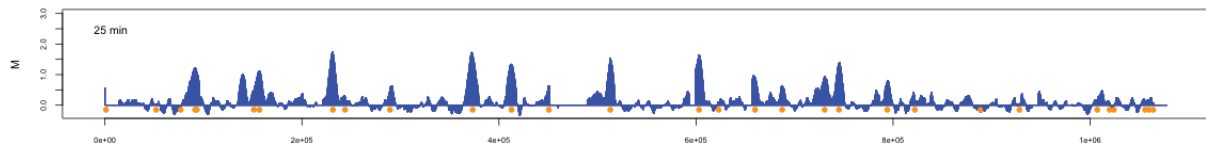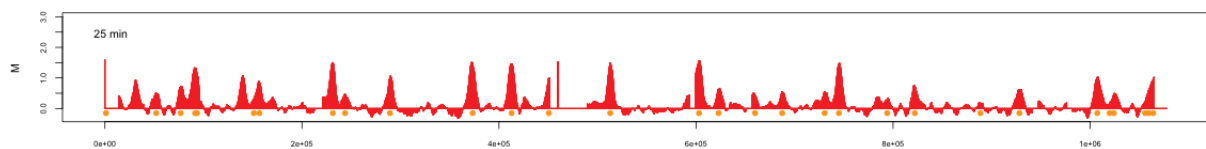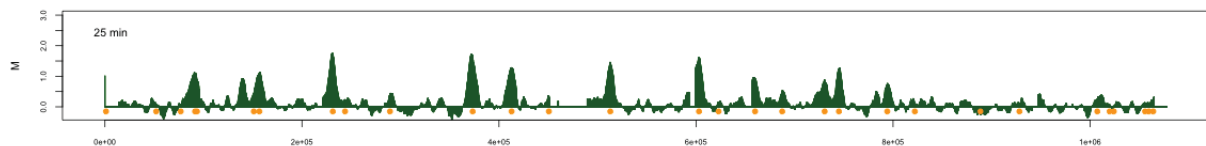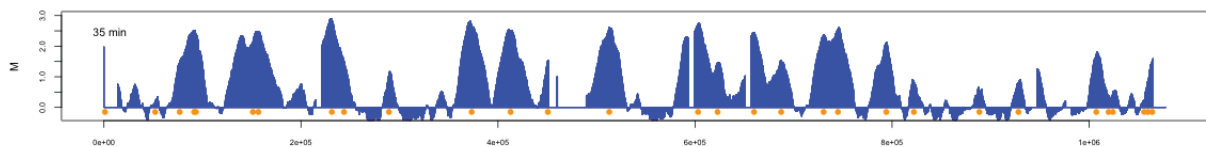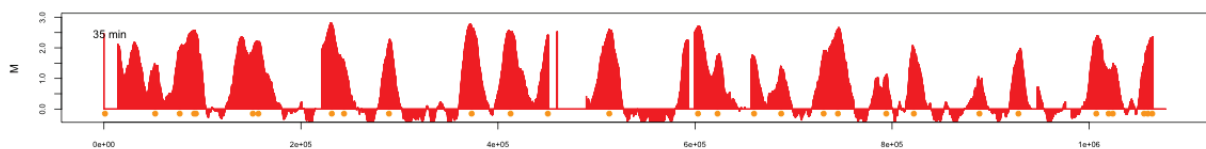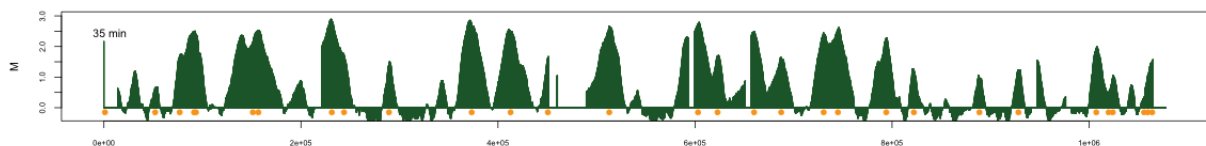

Supplement: Figure S3 — Temporal analysis of replication by BrdU-IP-chip for all chromosomes. Plots show average BrdU incorporation from duplicate experiments. Plot colors are keyed above. The lack of signal at origins (ARS305, ARS608, and ARS609, on chromosomes III and VI) in the mec1-100 strain is due to deletion of these origins, which was done for the purposes of a previous study, but is inconsequential for the current study. (ZIP) [file pone.0098501.s003.zip › FigS3/S3.12.pdf]

■ WT   ■ rif1Δ   ■ mec1-100

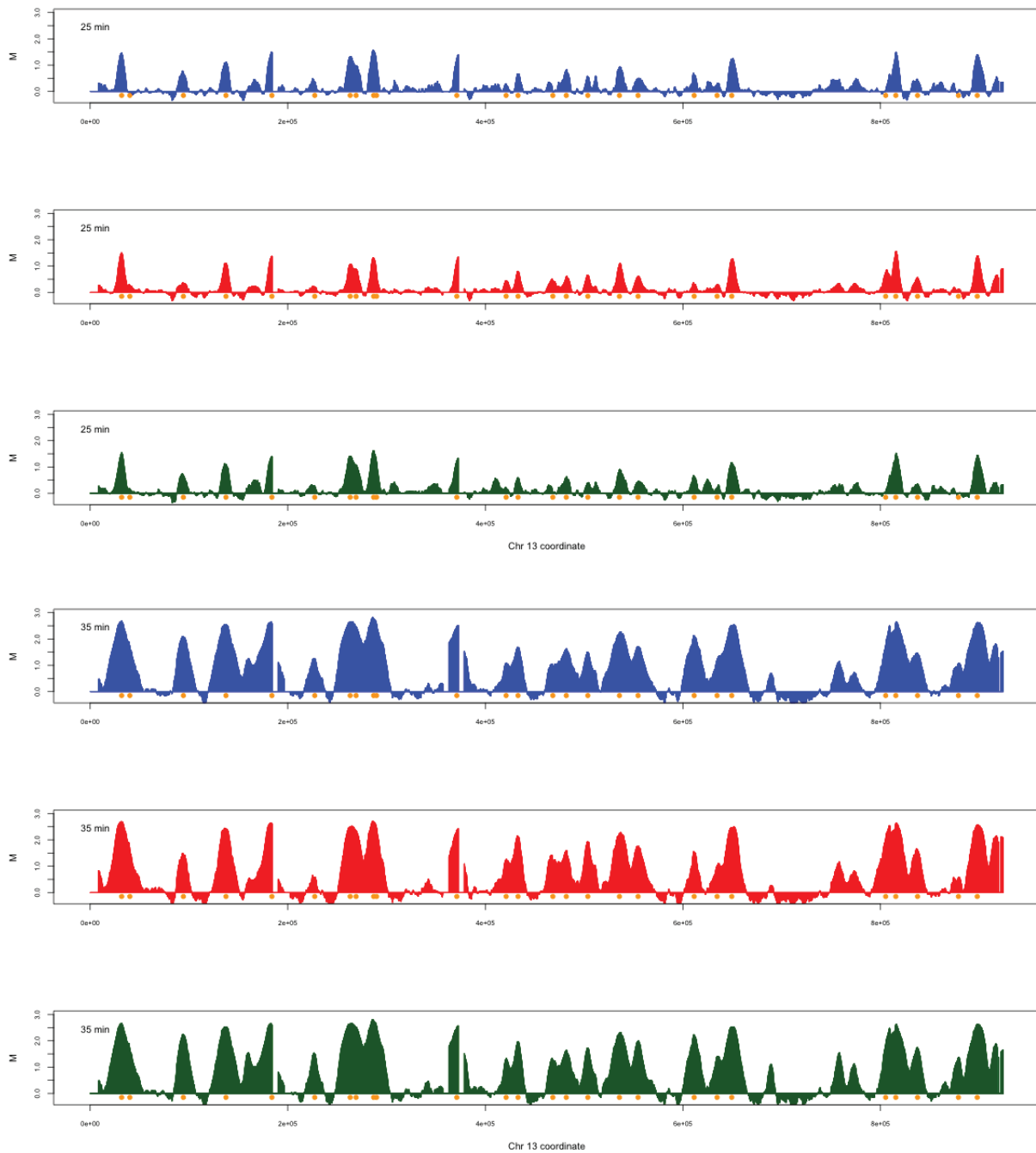

Supplement: Figure S3 — Temporal analysis of replication by BrdU-IP-chip for all chromosomes. Plots show average BrdU incorporation from duplicate experiments. Plot colors are keyed above. The lack of signal at origins (ARS305, ARS608, and ARS609, on chromosomes III and VI) in the mec1-100 strain is due to deletion of these origins, which was done for the purposes of a previous study, but is inconsequential for the current study. (ZIP) [file pone.0098501.s003.zip › FigS3/S3.13.pdf]

■ WT   ■ rif1Δ   ■ mec1-100

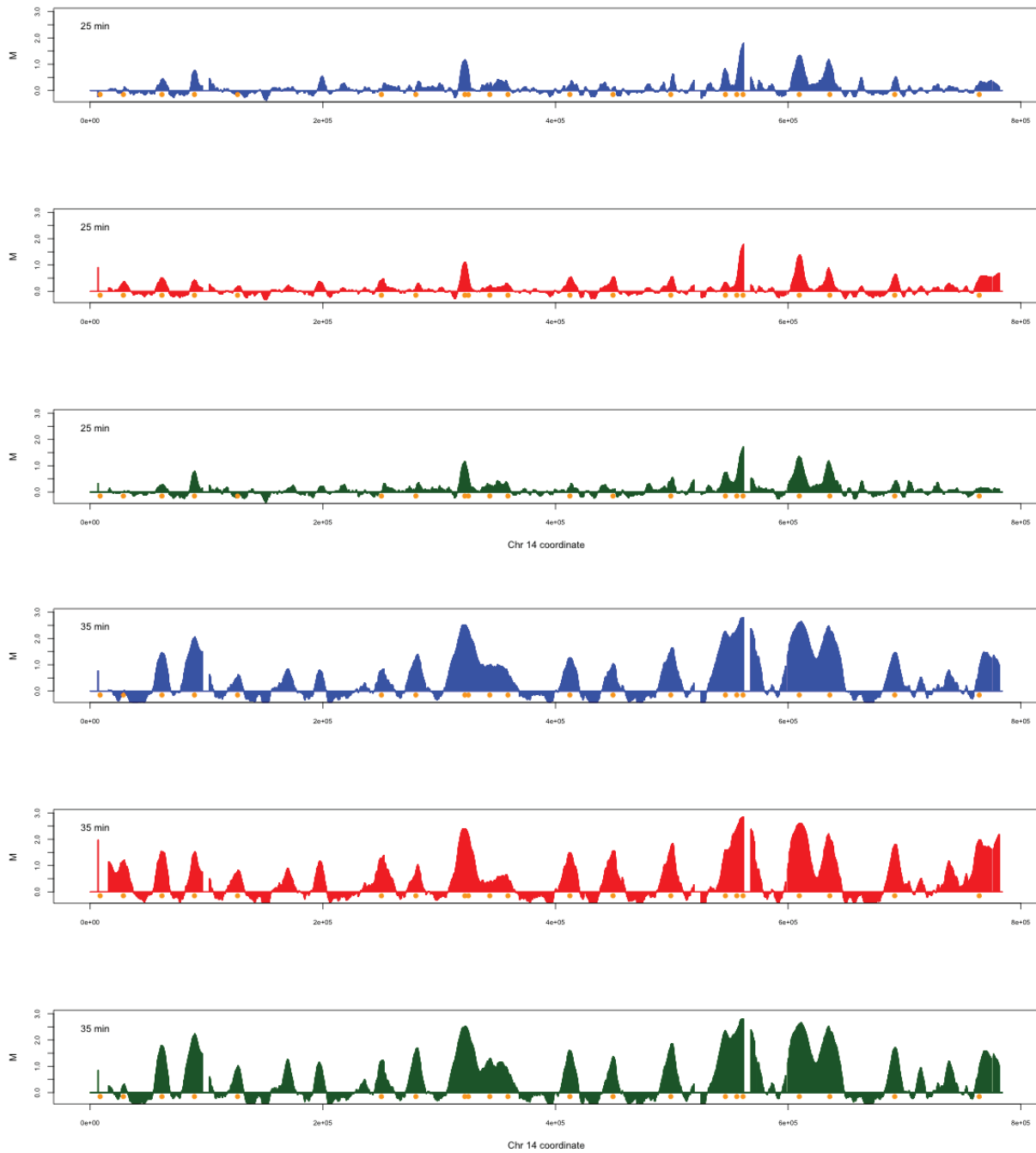

Supplement: Figure S3 — Temporal analysis of replication by BrdU-IP-chip for all chromosomes. Plots show average BrdU incorporation from duplicate experiments. Plot colors are keyed above. The lack of signal at origins (ARS305, ARS608, and ARS609, on chromosomes III and VI) in the mec1-100 strain is due to deletion of these origins, which was done for the purposes of a previous study, but is inconsequential for the current study. (ZIP) [file pone.0098501.s003.zip › FigS3/S3.14.pdf]

■ WT   ■ rif1Δ   ■ mec1-100

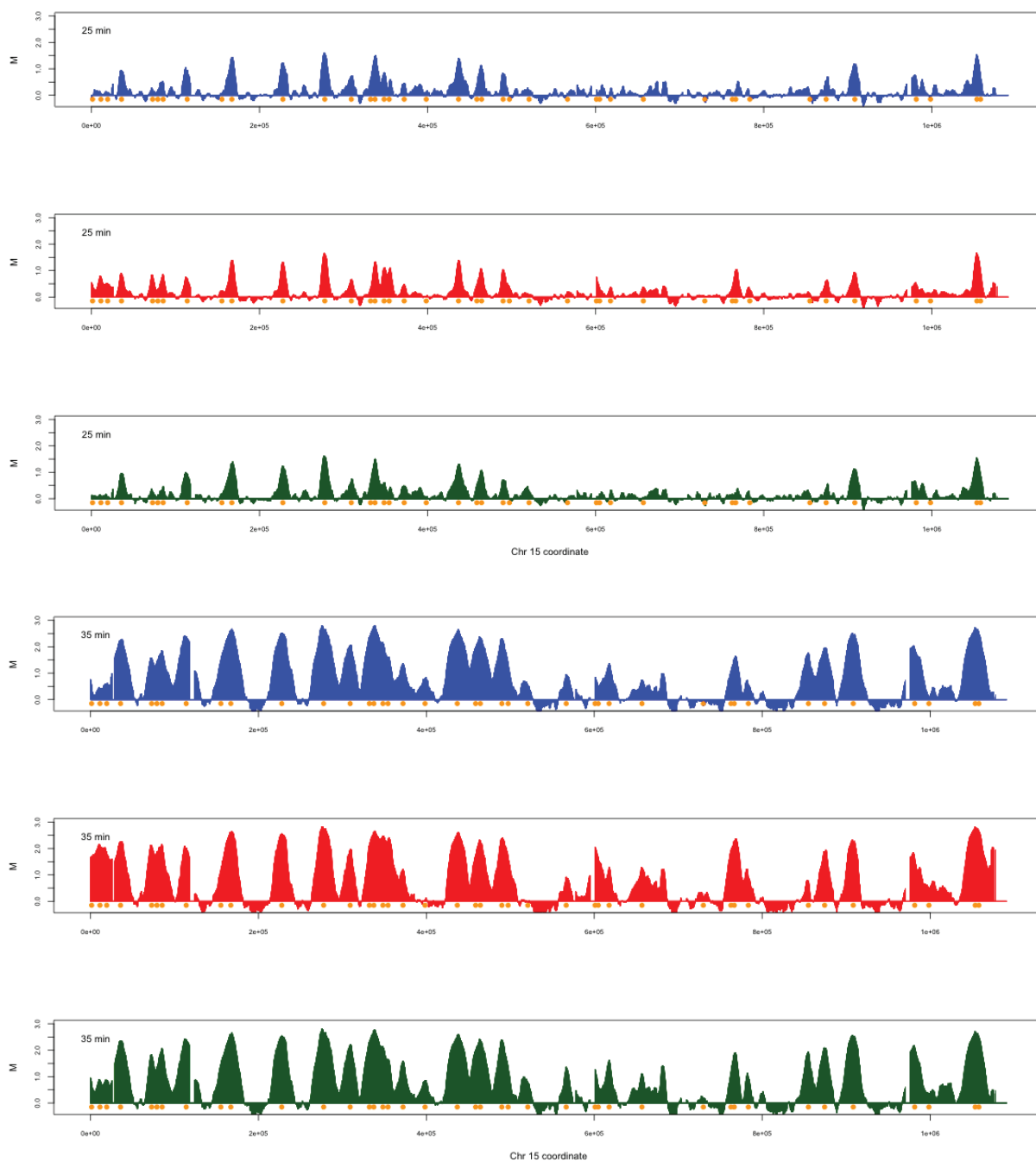

Supplement: Figure S3 — Temporal analysis of replication by BrdU-IP-chip for all chromosomes. Plots show average BrdU incorporation from duplicate experiments. Plot colors are keyed above. The lack of signal at origins (ARS305, ARS608, and ARS609, on chromosomes III and VI) in the mec1-100 strain is due to deletion of these origins, which was done for the purposes of a previous study, but is inconsequential for the current study. (ZIP) [file pone.0098501.s003.zip › FigS3/S3.15.pdf]

■ WT   ■ *rif1*Δ   ■ *mec1-100*

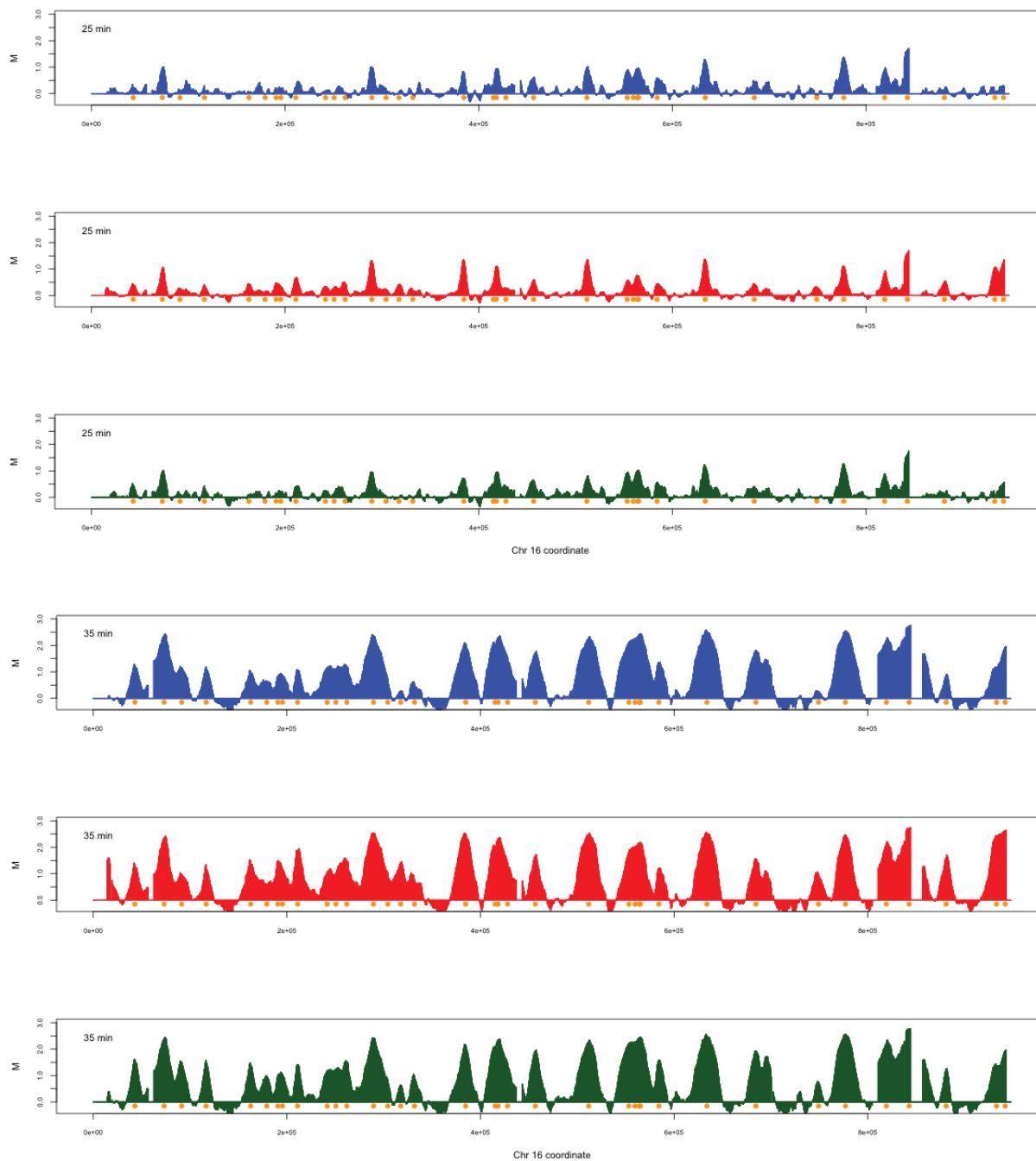

Supplement: Figure S3 — Temporal analysis of replication by BrdU-IP-chip for all chromosomes. Plots show average BrdU incorporation from duplicate experiments. Plot colors are keyed above. The lack of signal at origins (ARS305, ARS608, and ARS609, on chromosomes III and VI) in the mec1-100 strain is due to deletion of these origins, which was done for the purposes of a previous study, but is inconsequential for the current study. (ZIP) [file pone.0098501.s003.zip › FigS3/S3.16.pdf]

■ WT   ■ rif1Δ   ■ mec1-100

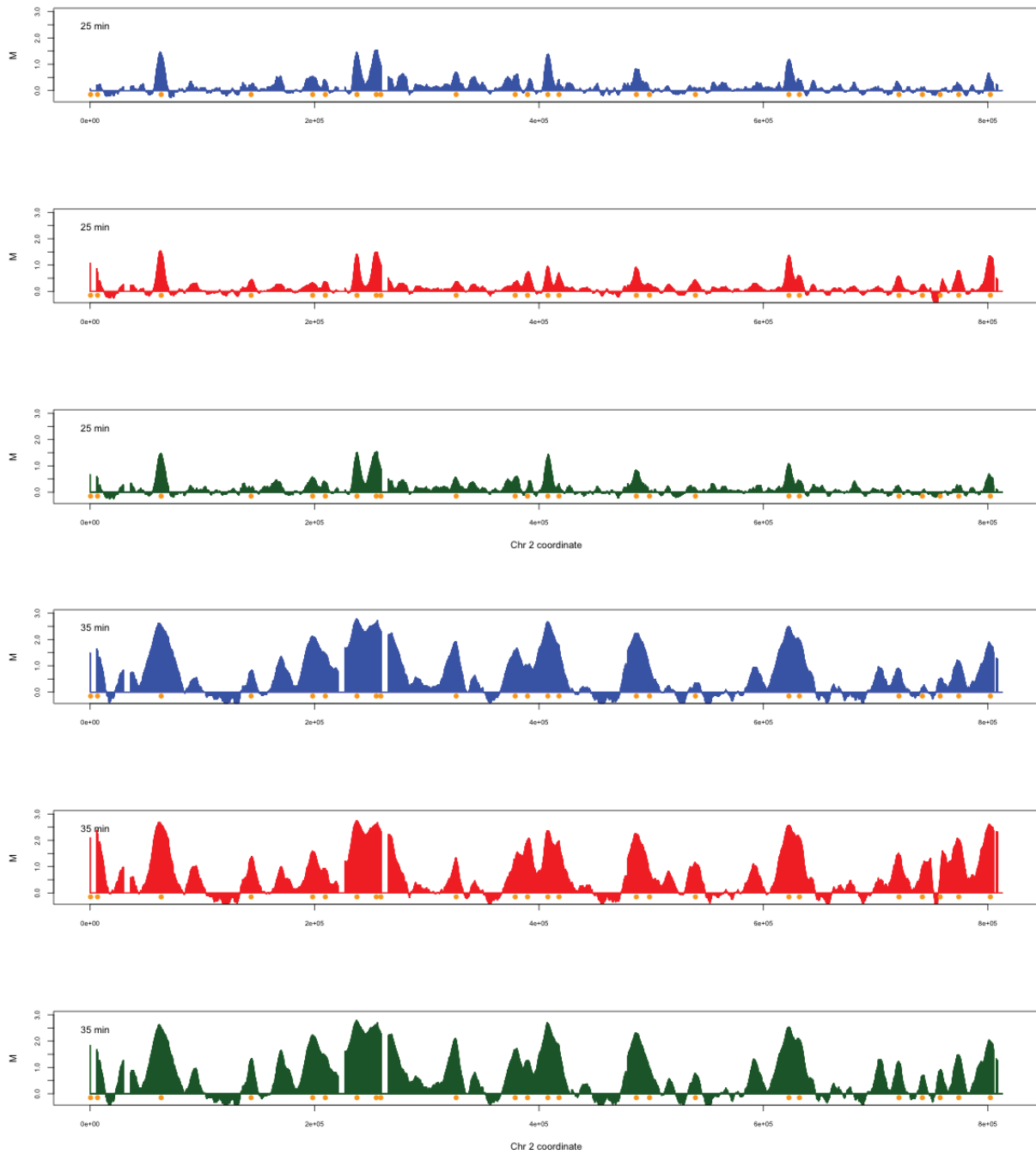

Supplement: Figure S3 — Temporal analysis of replication by BrdU-IP-chip for all chromosomes. Plots show average BrdU incorporation from duplicate experiments. Plot colors are keyed above. The lack of signal at origins (ARS305, ARS608, and ARS609, on chromosomes III and VI) in the mec1-100 strain is due to deletion of these origins, which was done for the purposes of a previous study, but is inconsequential for the current study. (ZIP) [file pone.0098501.s003.zip › FigS3/S3.2.pdf]

■ WT   ■ rif1Δ   ■ mec1-100

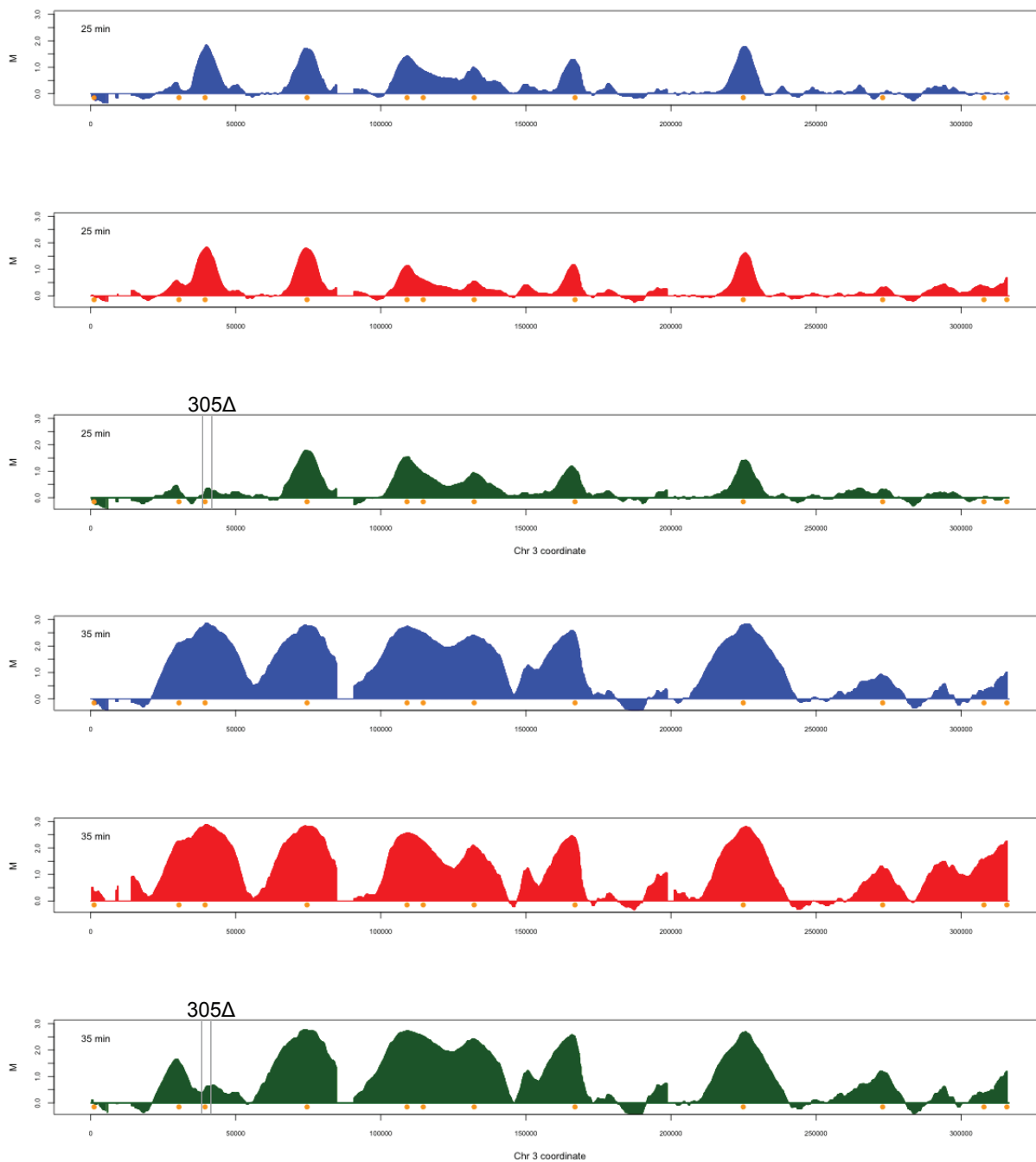

Supplement: Figure S3 — Temporal analysis of replication by BrdU-IP-chip for all chromosomes. Plots show average BrdU incorporation from duplicate experiments. Plot colors are keyed above. The lack of signal at origins (ARS305, ARS608, and ARS609, on chromosomes III and VI) in the mec1-100 strain is due to deletion of these origins, which was done for the purposes of a previous study, but is inconsequential for the current study. (ZIP) [file pone.0098501.s003.zip › FigS3/S3.3.pdf]

■ WT   ■ rif1Δ   ■ mec1-100

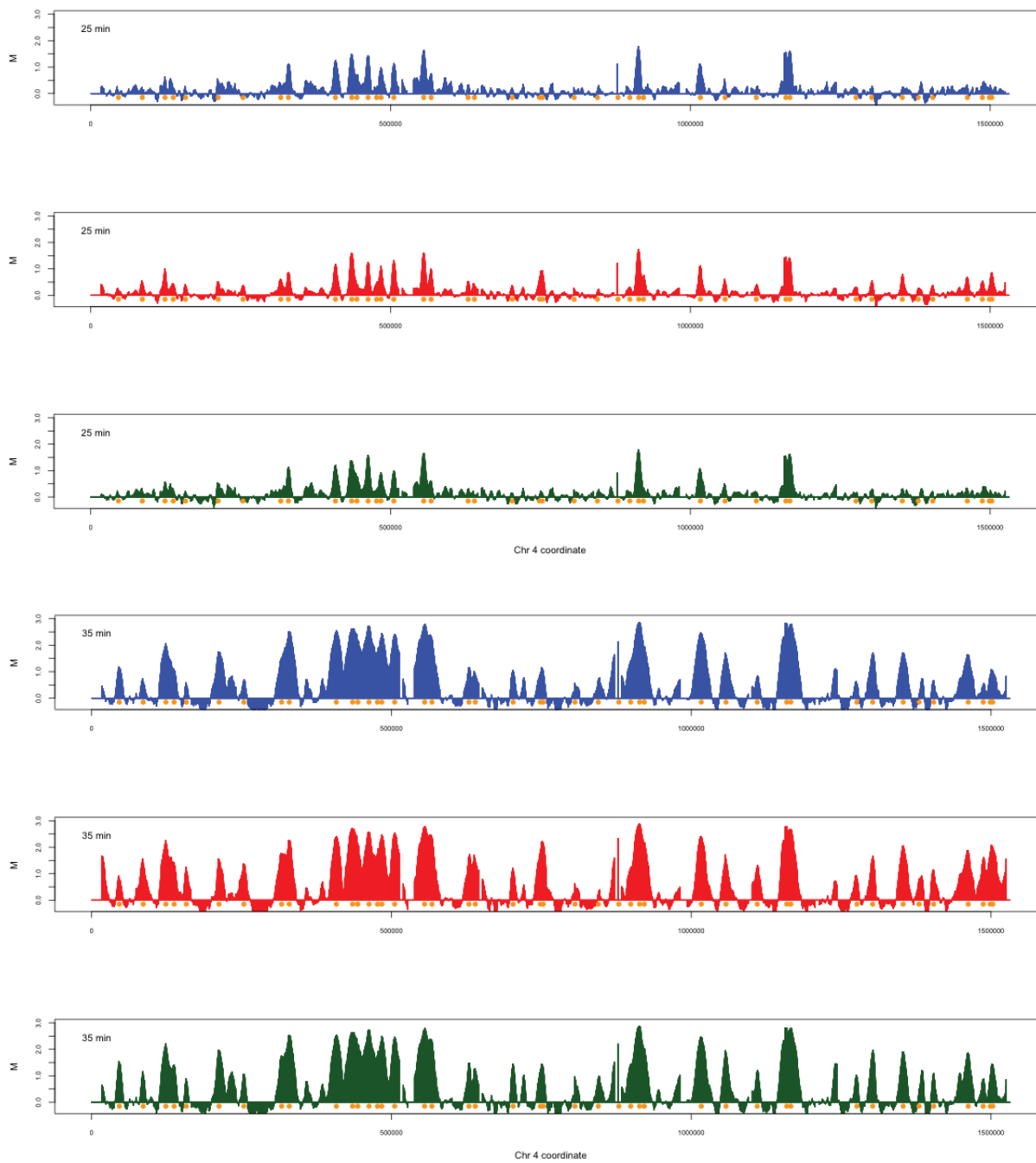

Supplement: Figure S3 — Temporal analysis of replication by BrdU-IP-chip for all chromosomes. Plots show average BrdU incorporation from duplicate experiments. Plot colors are keyed above. The lack of signal at origins (ARS305, ARS608, and ARS609, on chromosomes III and VI) in the mec1-100 strain is due to deletion of these origins, which was done for the purposes of a previous study, but is inconsequential for the current study. (ZIP) [file pone.0098501.s003.zip › FigS3/S3.4.pdf]

■ WT   ■ rif1Δ   ■ mec1-100

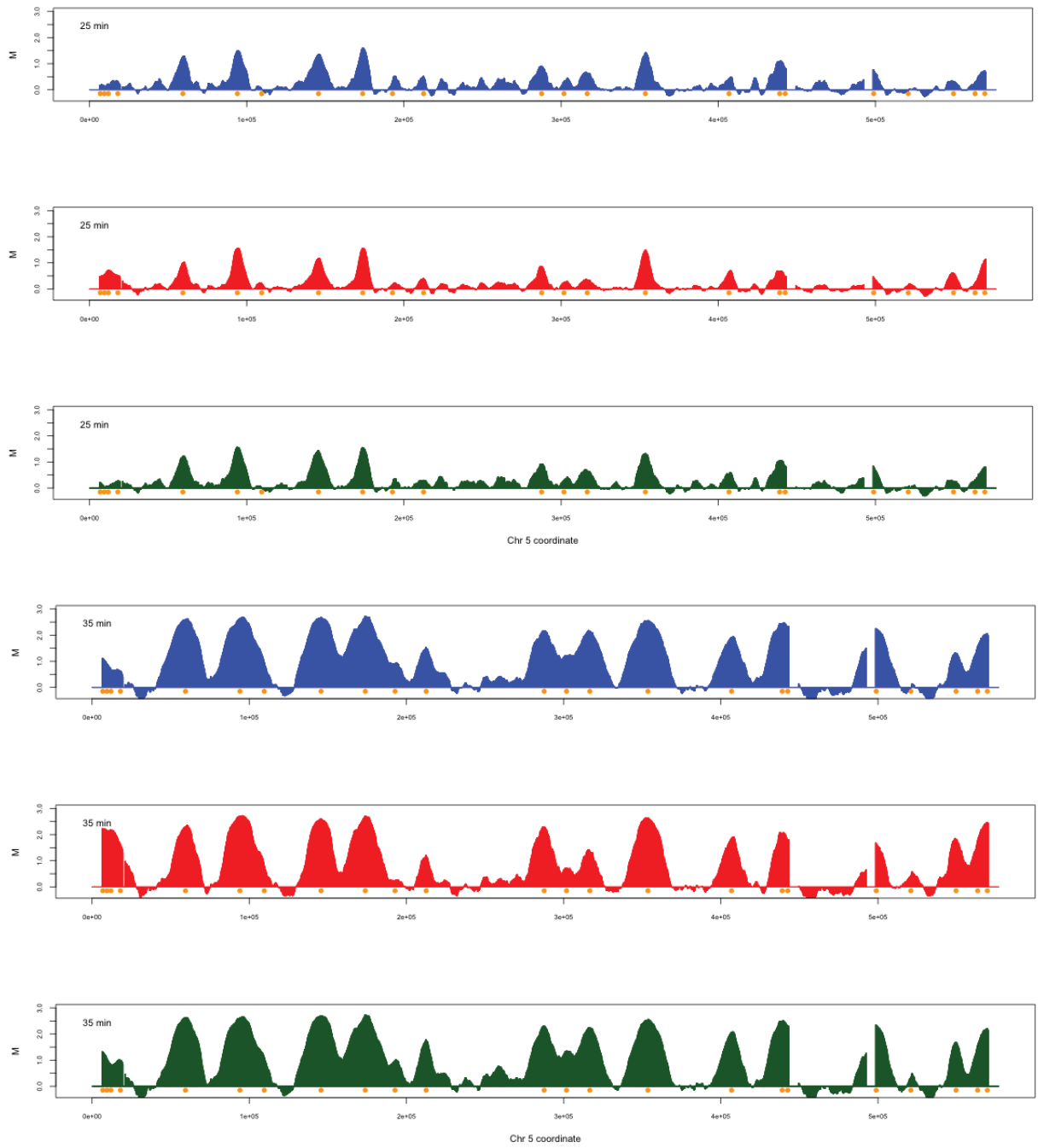

Supplement: Figure S3 — Temporal analysis of replication by BrdU-IP-chip for all chromosomes. Plots show average BrdU incorporation from duplicate experiments. Plot colors are keyed above. The lack of signal at origins (ARS305, ARS608, and ARS609, on chromosomes III and VI) in the mec1-100 strain is due to deletion of these origins, which was done for the purposes of a previous study, but is inconsequential for the current study. (ZIP) [file pone.0098501.s003.zip › FigS3/S3.5.pdf]

■ WT   ■ rif1Δ   ■ mec1-100

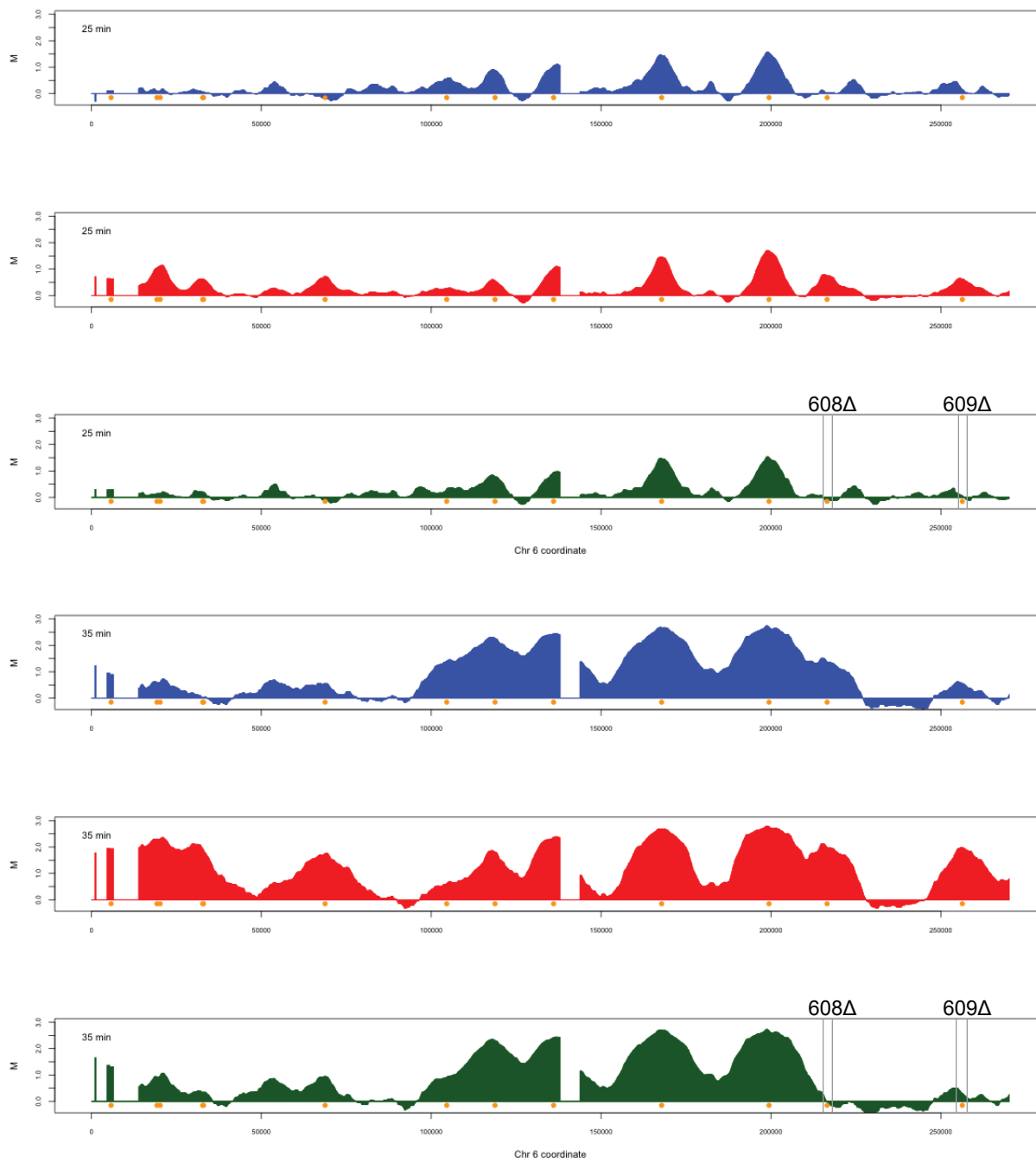

Supplement: Figure S3 — Temporal analysis of replication by BrdU-IP-chip for all chromosomes. Plots show average BrdU incorporation from duplicate experiments. Plot colors are keyed above. The lack of signal at origins (ARS305, ARS608, and ARS609, on chromosomes III and VI) in the mec1-100 strain is due to deletion of these origins, which was done for the purposes of a previous study, but is inconsequential for the current study. (ZIP) [file pone.0098501.s003.zip › FigS3/S3.6.pdf]

■ WT   ■ rif1Δ   ■ mec1-100

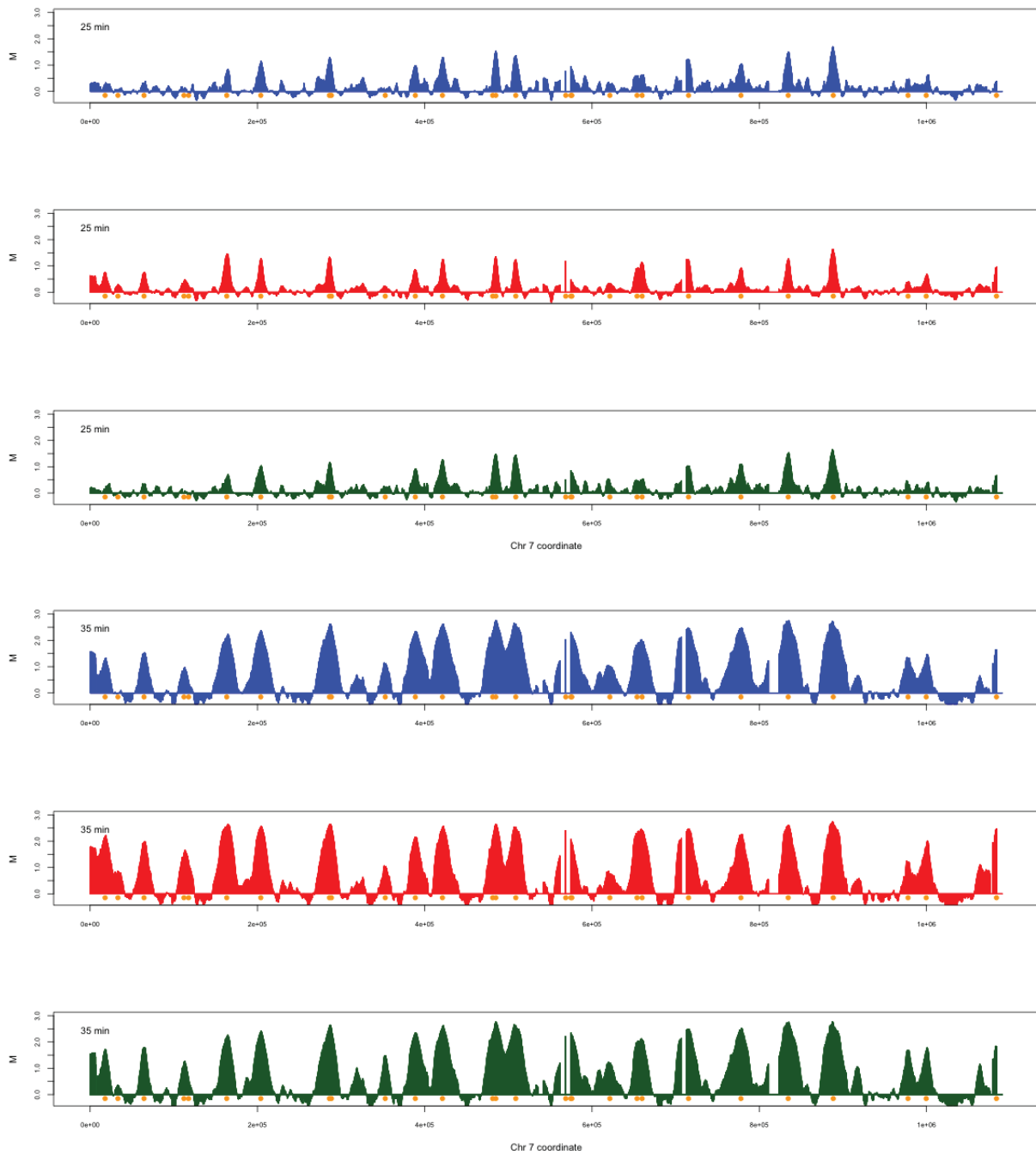

Supplement: Figure S3 — Temporal analysis of replication by BrdU-IP-chip for all chromosomes. Plots show average BrdU incorporation from duplicate experiments. Plot colors are keyed above. The lack of signal at origins (ARS305, ARS608, and ARS609, on chromosomes III and VI) in the mec1-100 strain is due to deletion of these origins, which was done for the purposes of a previous study, but is inconsequential for the current study. (ZIP) [file pone.0098501.s003.zip › FigS3/S3.7.pdf]

■ WT   ■ rif1Δ   ■ mec1-100

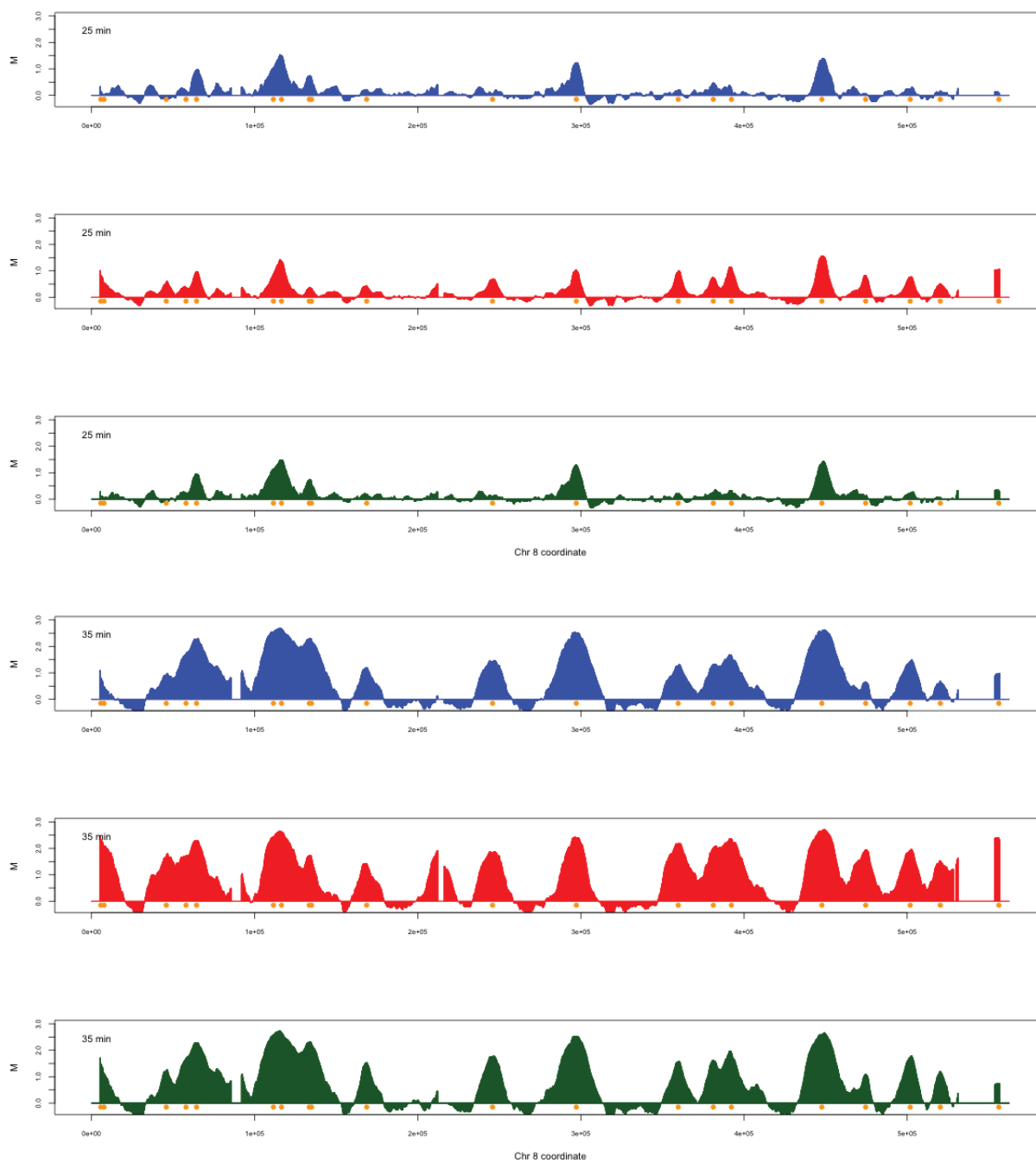

Supplement: Figure S3 — Temporal analysis of replication by BrdU-IP-chip for all chromosomes. Plots show average BrdU incorporation from duplicate experiments. Plot colors are keyed above. The lack of signal at origins (ARS305, ARS608, and ARS609, on chromosomes III and VI) in the mec1-100 strain is due to deletion of these origins, which was done for the purposes of a previous study, but is inconsequential for the current study. (ZIP) [file pone.0098501.s003.zip › FigS3/S3.8.pdf]

■ WT   ■ rif1Δ   ■ mec1-100

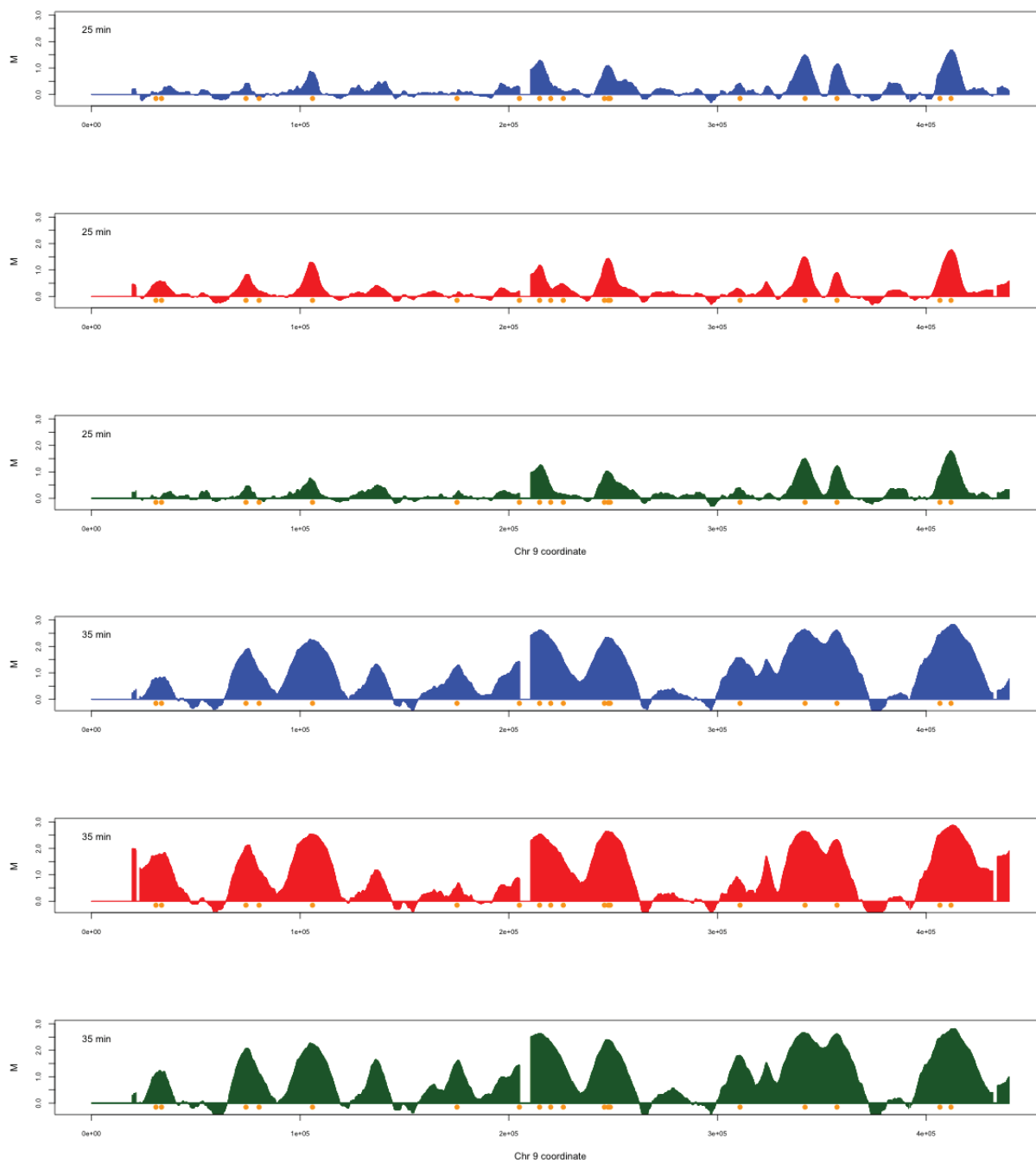

Supplement: Figure S3 — Temporal analysis of replication by BrdU-IP-chip for all chromosomes. Plots show average BrdU incorporation from duplicate experiments. Plot colors are keyed above. The lack of signal at origins (ARS305, ARS608, and ARS609, on chromosomes III and VI) in the mec1-100 strain is due to deletion of these origins, which was done for the purposes of a previous study, but is inconsequential for the current study. (ZIP) [file pone.0098501.s003.zip › FigS3/S3.9.pdf]

■ *sml1Δ*   ■ *sml1Δ rif1Δ*   ■ *sml1Δ mec1Δ*

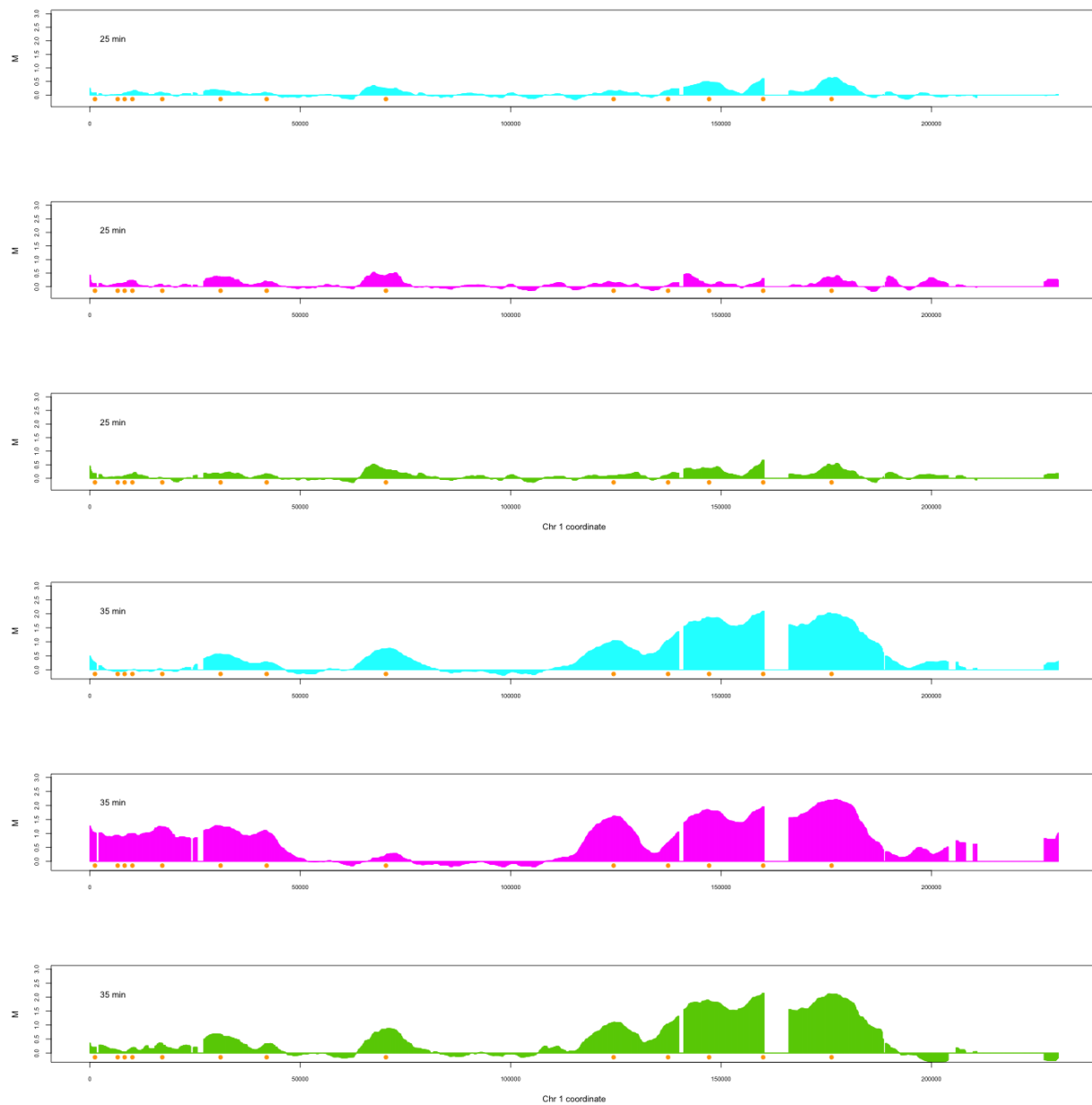

Supplement: Figure S4 — Temporal analysis of replication by BrdU-IP-chip for all chromosomes. Plots show average BrdU incorporation from duplicate experiments. Plot colors are keyed above. (ZIP) [file pone.0098501.s004.zip › FigS4/S4.1.pdf]

■ *sml1Δ*   ■ *sml1Δ rif1Δ*   ■ *sml1Δ mec1Δ*

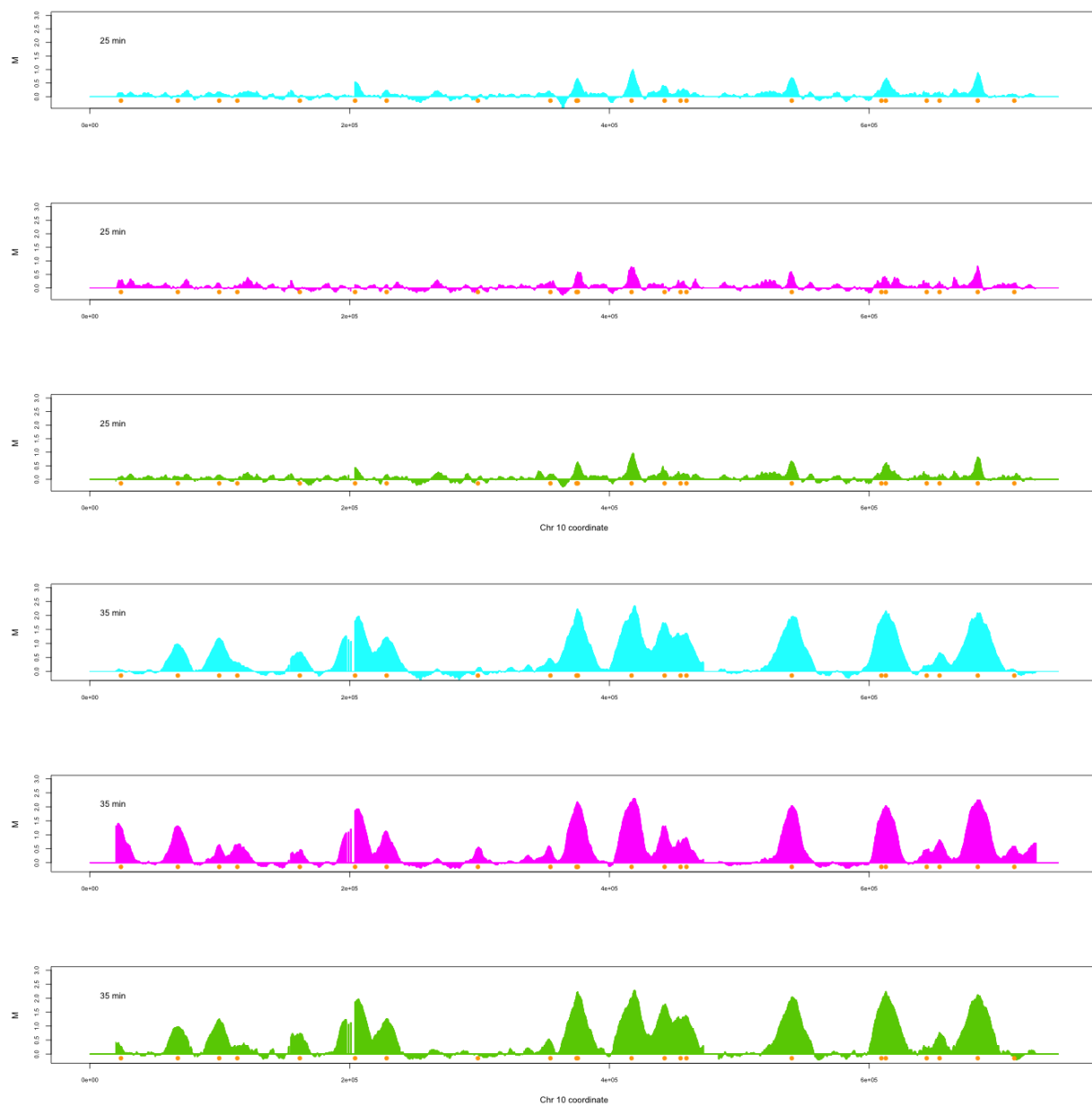

Supplement: Figure S4 — Temporal analysis of replication by BrdU-IP-chip for all chromosomes. Plots show average BrdU incorporation from duplicate experiments. Plot colors are keyed above. (ZIP) [file pone.0098501.s004.zip › FigS4/S4.10.pdf]

■ *sml1Δ*   ■ *sml1Δ rif1Δ*   ■ *sml1Δ mec1Δ*

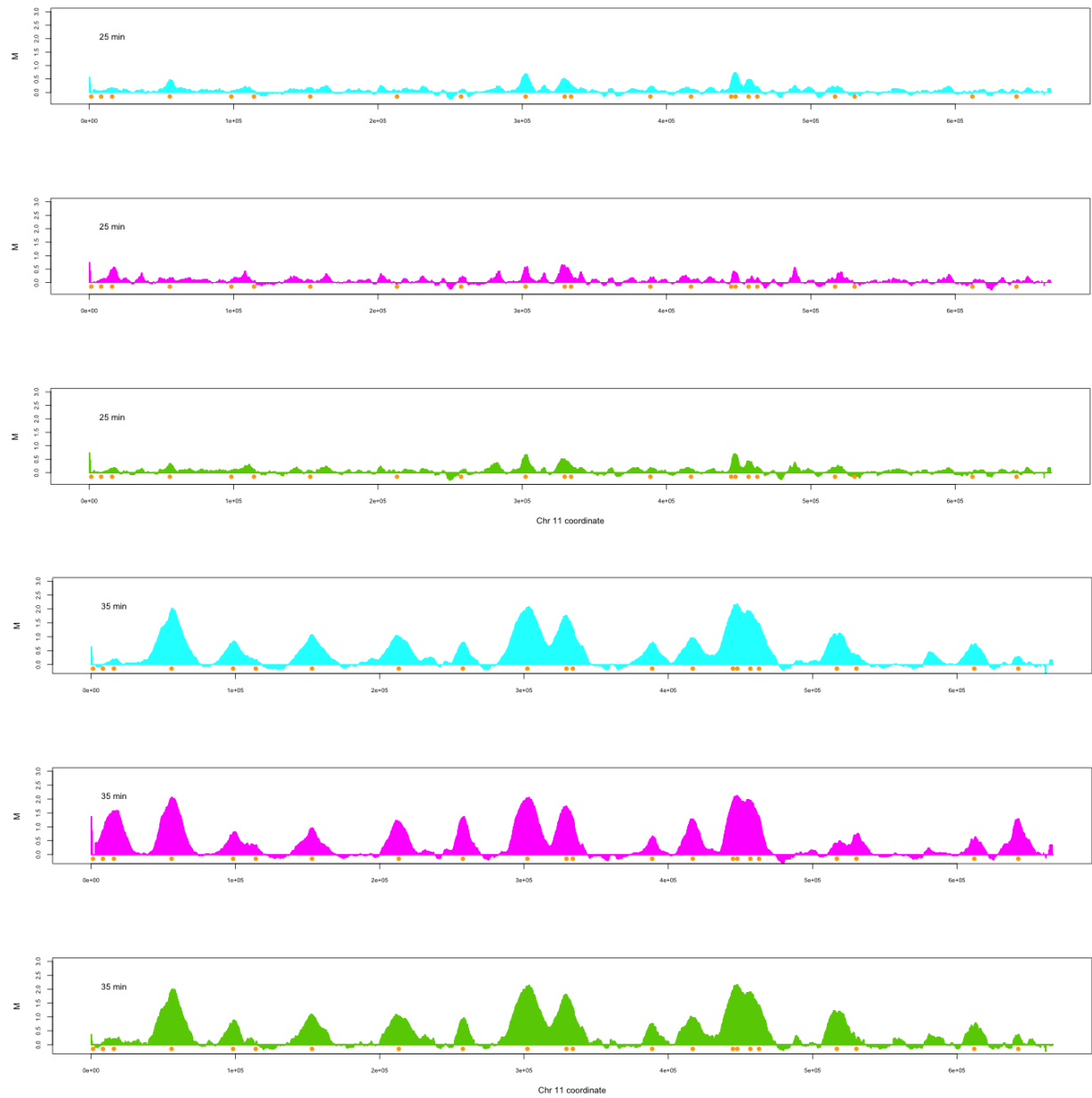

Supplement: Figure S4 — Temporal analysis of replication by BrdU-IP-chip for all chromosomes. Plots show average BrdU incorporation from duplicate experiments. Plot colors are keyed above. (ZIP) [file pone.0098501.s004.zip › FigS4/S4.11.pdf]

■ *sml1Δ*   ■ *sml1Δ rif1Δ*   ■ *sml1Δ mec1Δ*

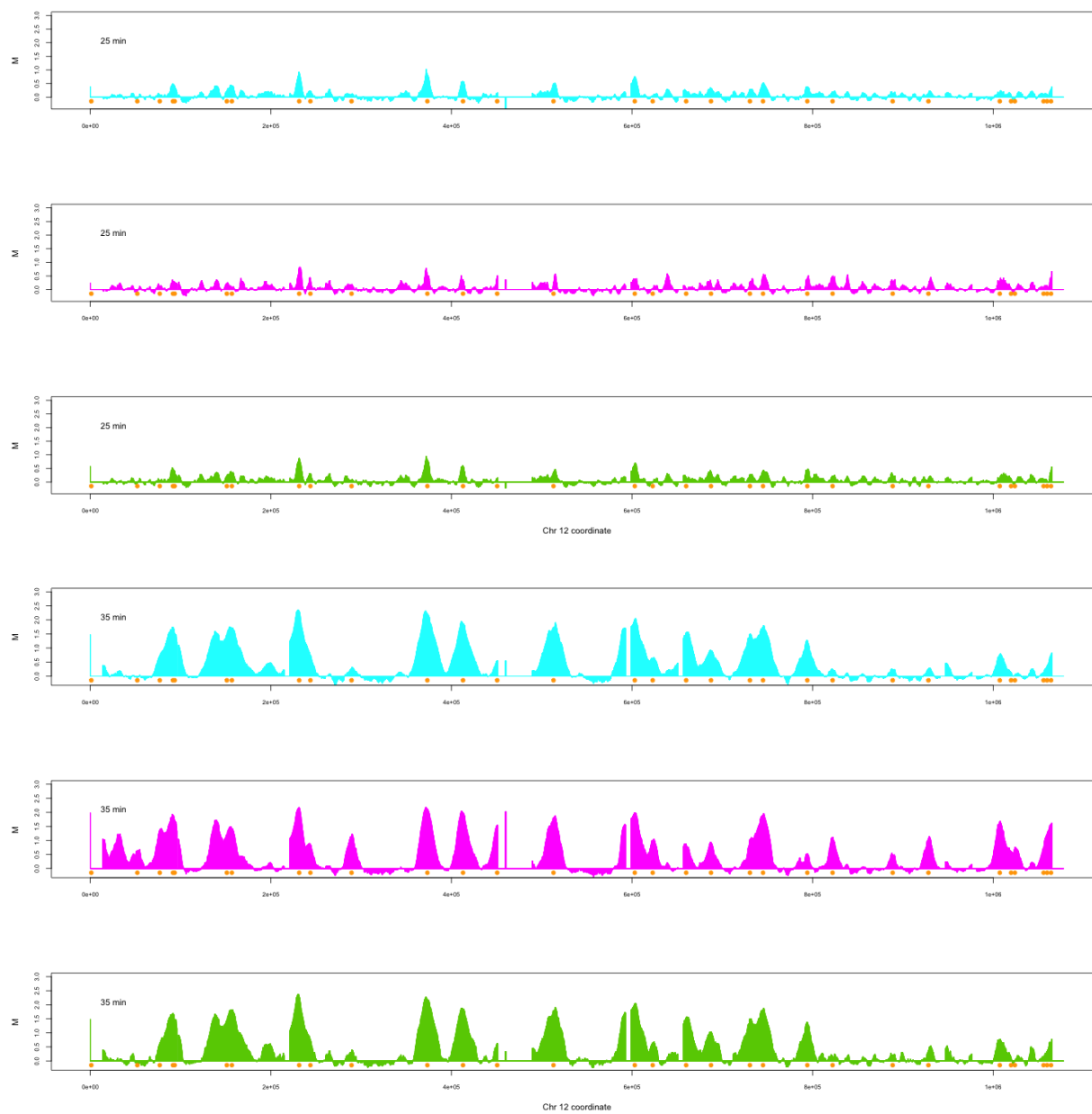

Supplement: Figure S4 — Temporal analysis of replication by BrdU-IP-chip for all chromosomes. Plots show average BrdU incorporation from duplicate experiments. Plot colors are keyed above. (ZIP) [file pone.0098501.s004.zip › FigS4/S4.12.pdf]

■ *sml1Δ*   ■ *sml1Δ rif1Δ*   ■ *sml1Δ mec1Δ*

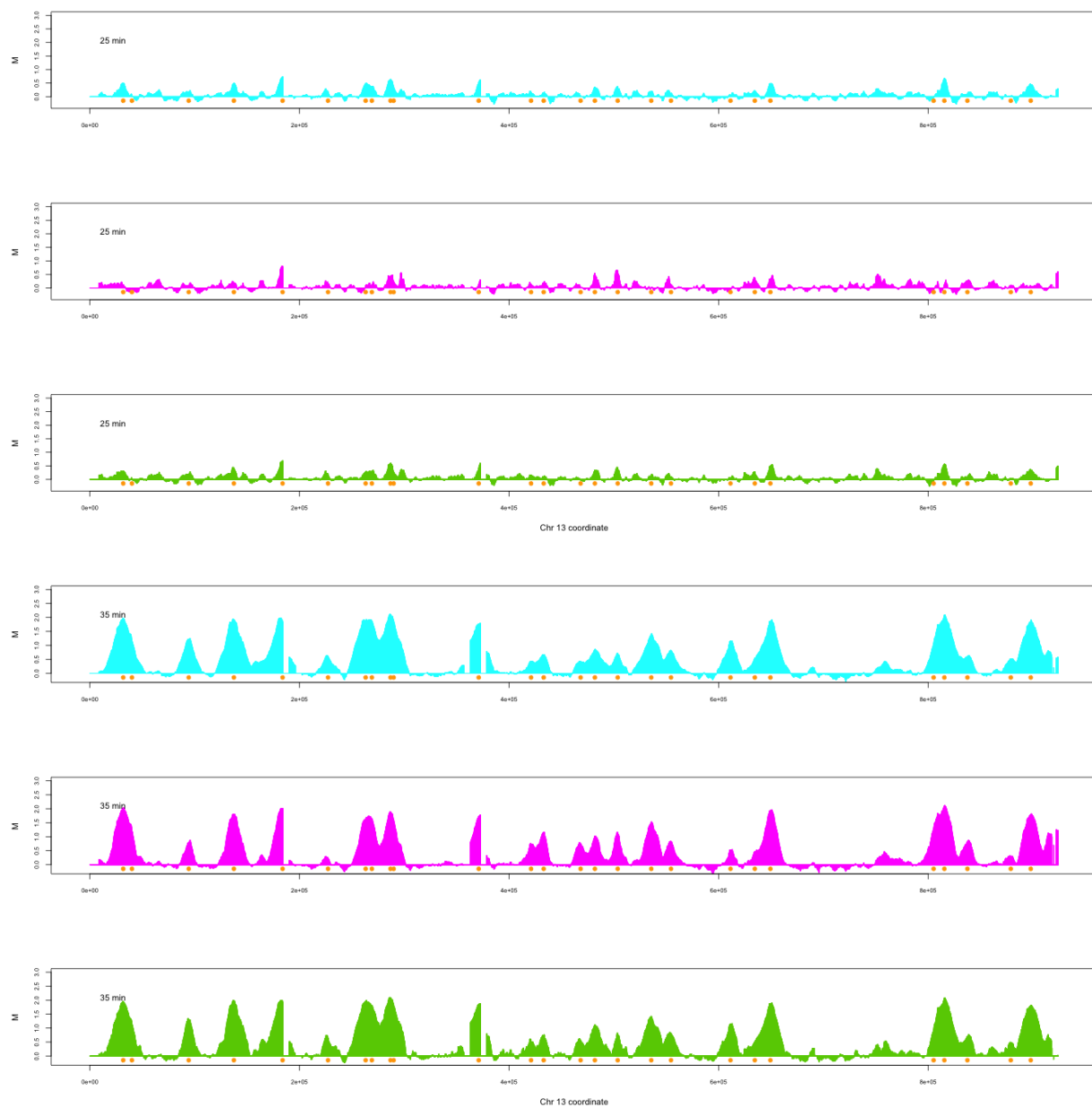

Supplement: Figure S4 — Temporal analysis of replication by BrdU-IP-chip for all chromosomes. Plots show average BrdU incorporation from duplicate experiments. Plot colors are keyed above. (ZIP) [file pone.0098501.s004.zip › FigS4/S4.13.pdf]

■ *sml1Δ*   ■ *sml1Δ rif1Δ*   ■ *sml1Δ mec1Δ*

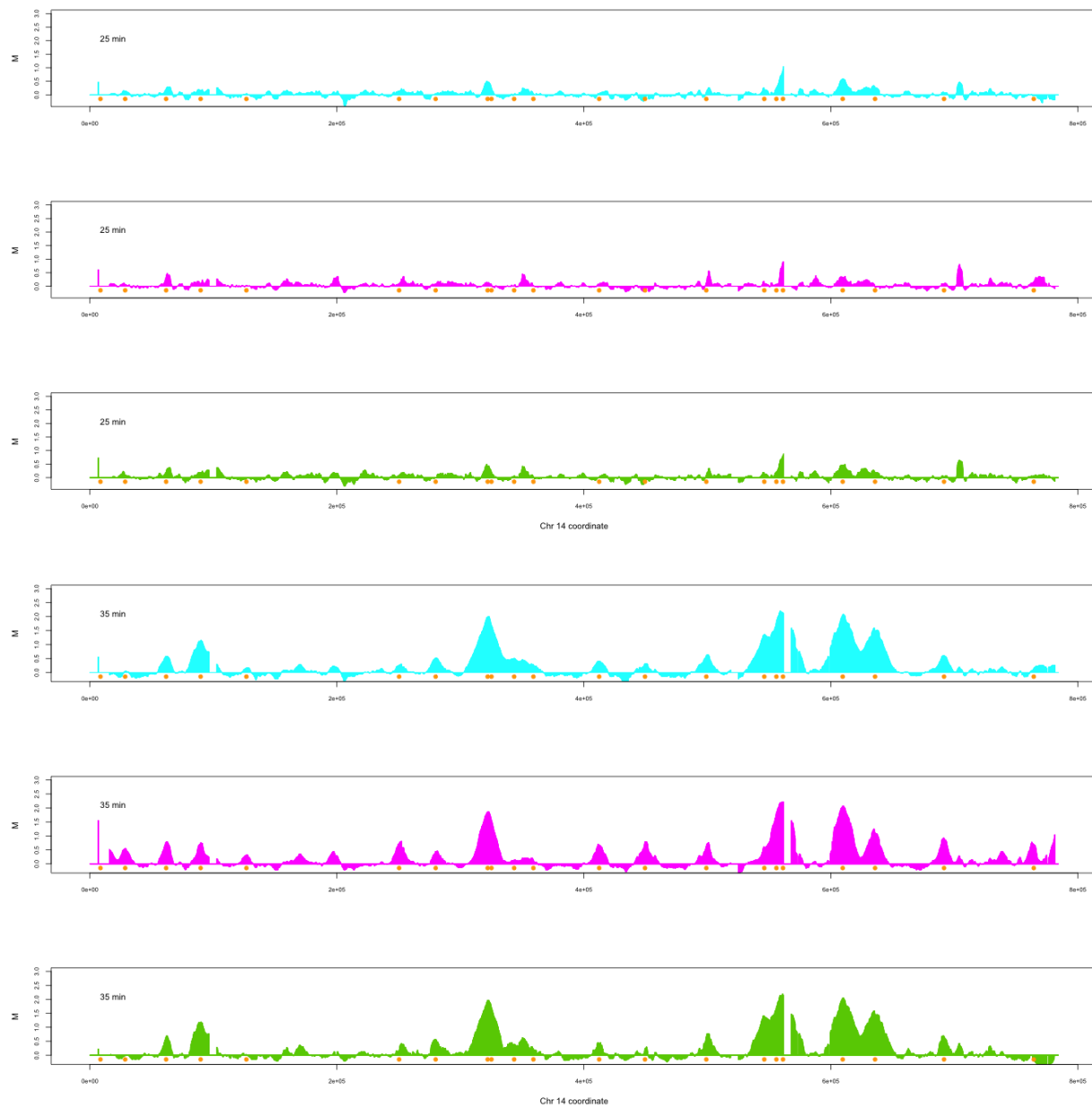

Supplement: Figure S4 — Temporal analysis of replication by BrdU-IP-chip for all chromosomes. Plots show average BrdU incorporation from duplicate experiments. Plot colors are keyed above. (ZIP) [file pone.0098501.s004.zip › FigS4/S4.14.pdf]

■ *sml1Δ*   ■ *sml1Δ rif1Δ*   ■ *sml1Δ mec1Δ*

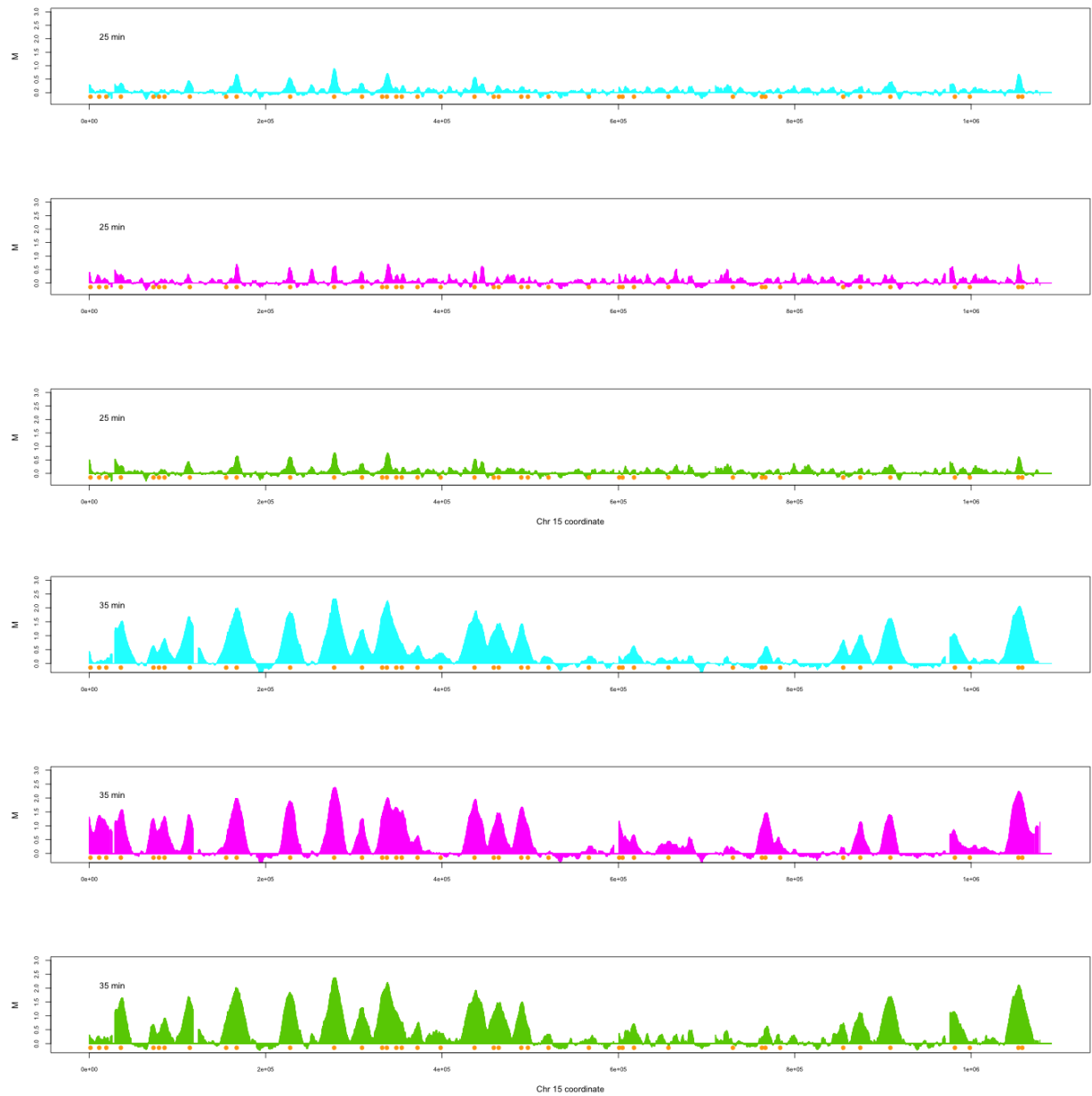

Supplement: Figure S4 — Temporal analysis of replication by BrdU-IP-chip for all chromosomes. Plots show average BrdU incorporation from duplicate experiments. Plot colors are keyed above. (ZIP) [file pone.0098501.s004.zip › FigS4/S4.15.pdf]

■ *sml1Δ*   ■ *sml1Δ rif1Δ*   ■ *sml1Δ mec1Δ*

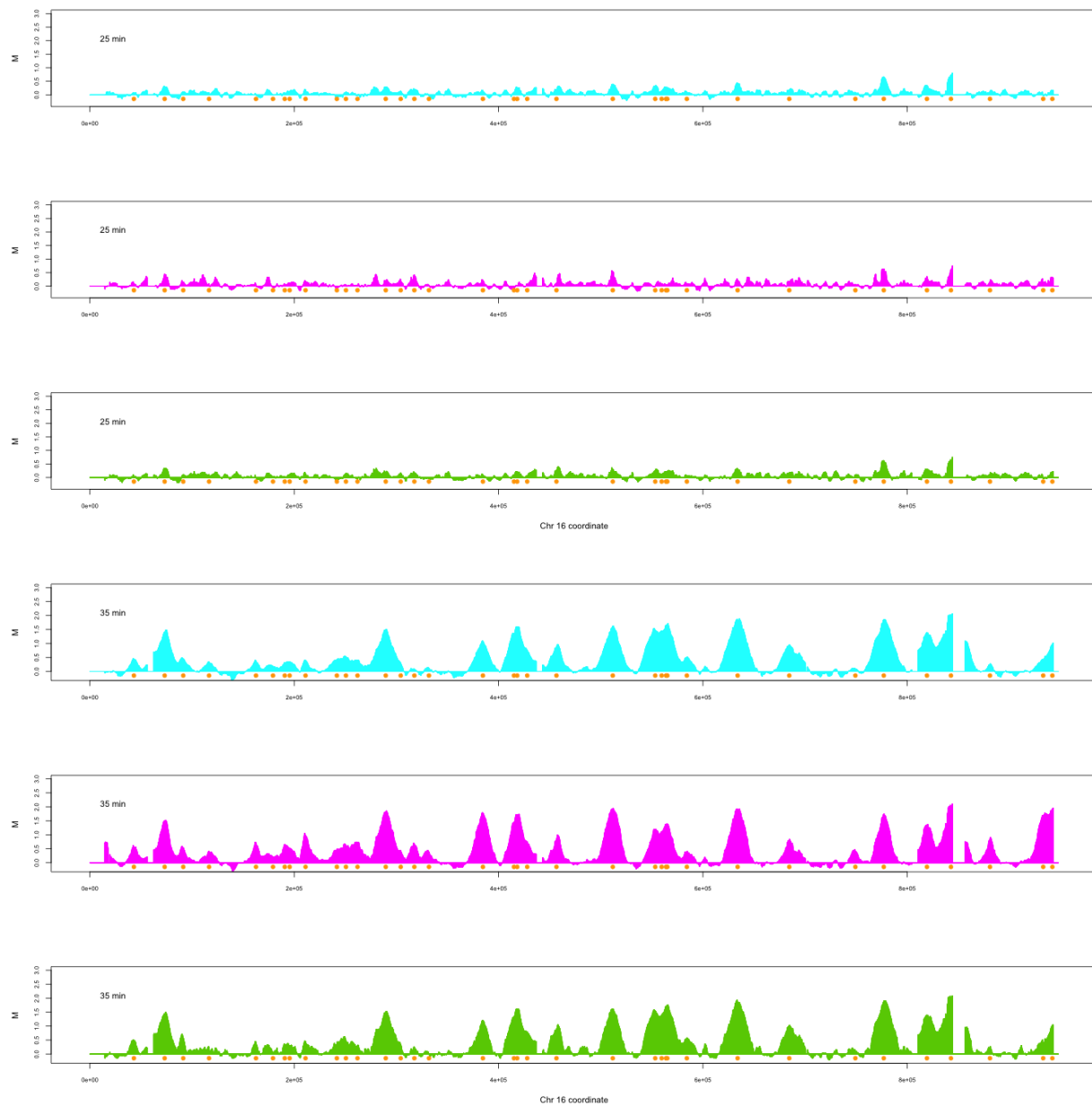

Supplement: Figure S4 — Temporal analysis of replication by BrdU-IP-chip for all chromosomes. Plots show average BrdU incorporation from duplicate experiments. Plot colors are keyed above. (ZIP) [file pone.0098501.s004.zip › FigS4/S4.16.pdf]

■ *sml1Δ*   ■ *sml1Δ rif1Δ*   ■ *sml1Δ mec1Δ*

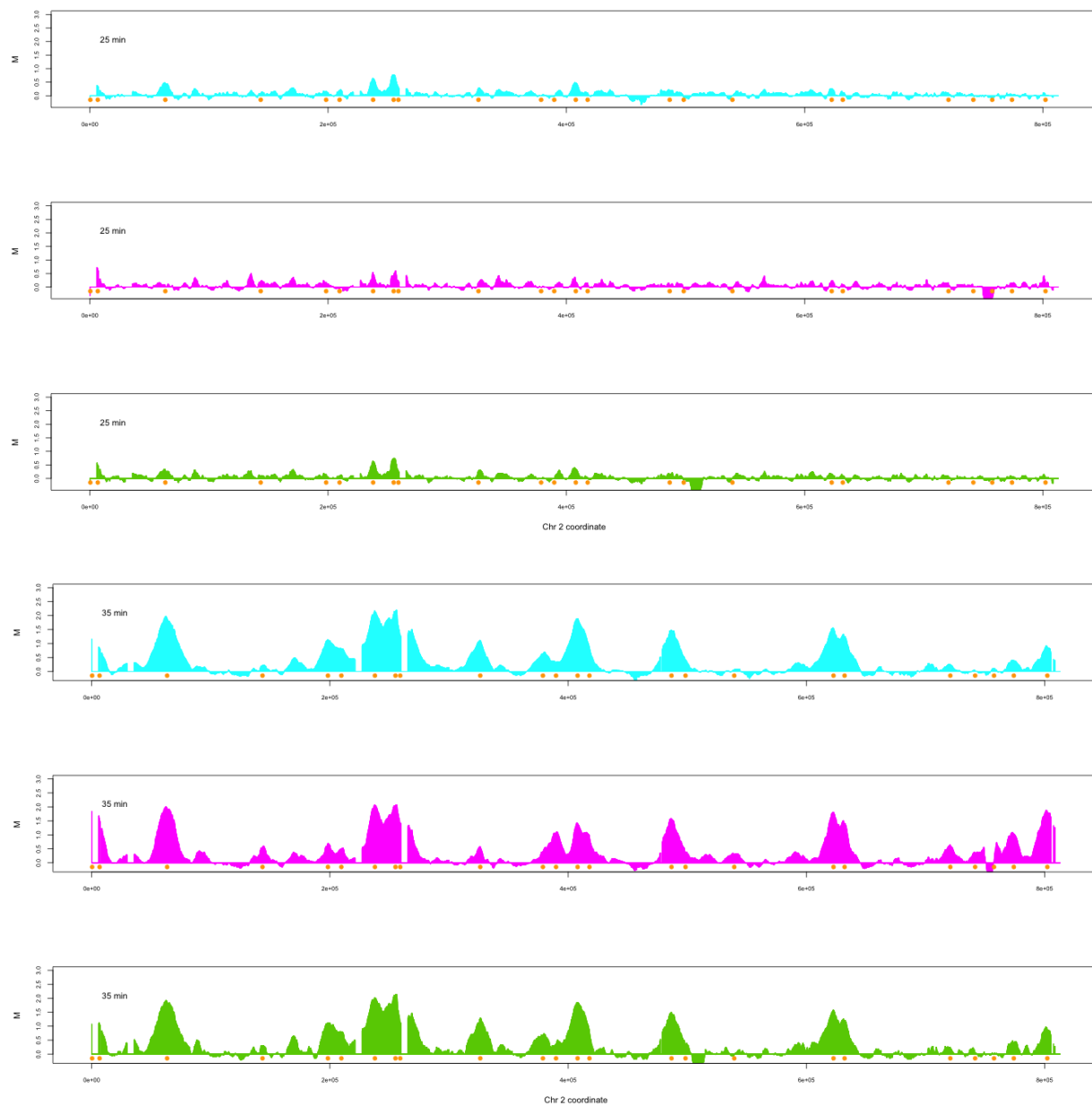

Supplement: Figure S4 — Temporal analysis of replication by BrdU-IP-chip for all chromosomes. Plots show average BrdU incorporation from duplicate experiments. Plot colors are keyed above. (ZIP) [file pone.0098501.s004.zip › FigS4/S4.2.pdf]

■ *sml1Δ*   ■ *sml1Δ rif1Δ*   ■ *sml1Δ mec1Δ*

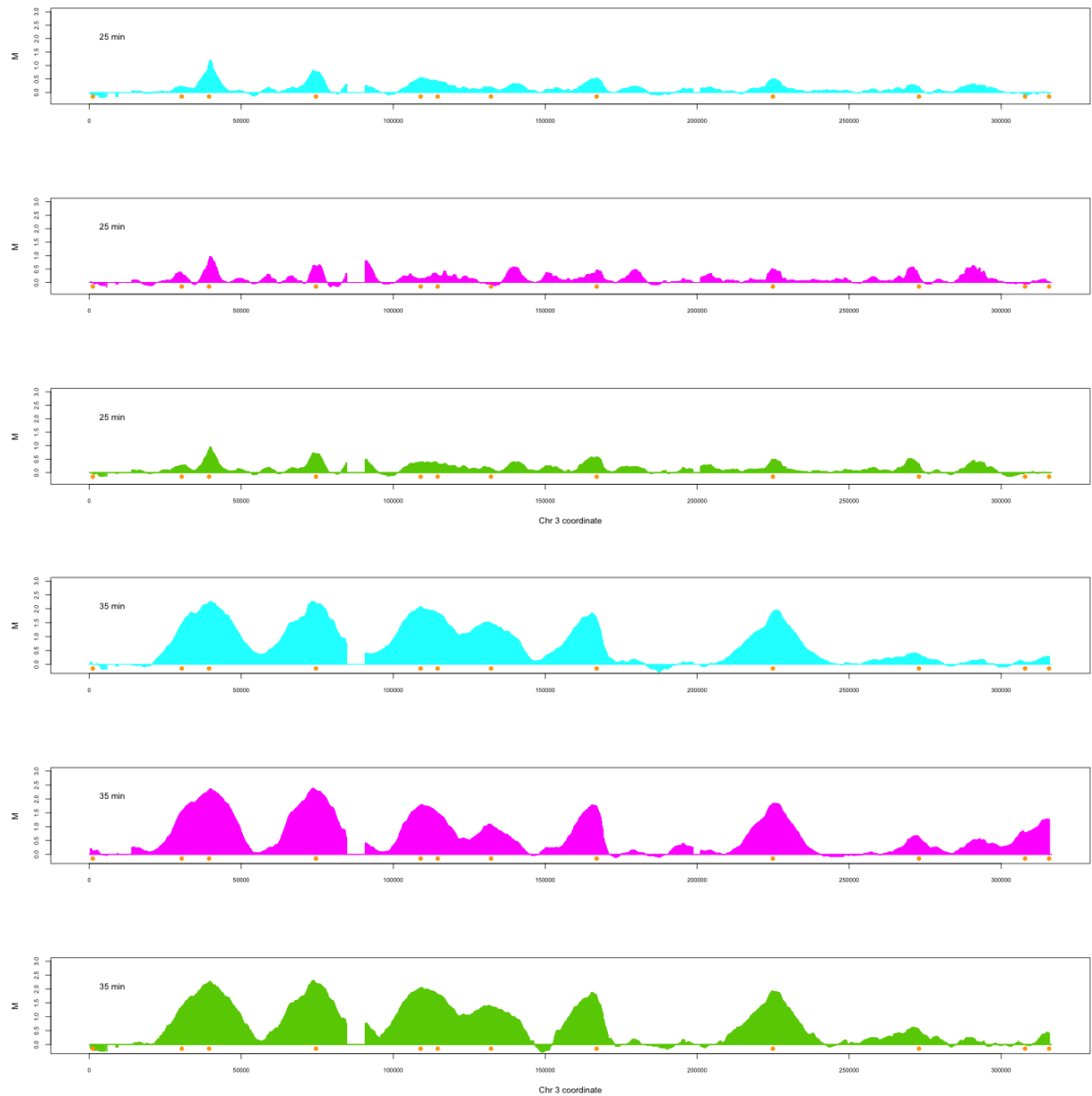

Supplement: Figure S4 — Temporal analysis of replication by BrdU-IP-chip for all chromosomes. Plots show average BrdU incorporation from duplicate experiments. Plot colors are keyed above. (ZIP) [file pone.0098501.s004.zip › FigS4/S4.3.pdf]

■ *sml1Δ*   ■ *sml1Δ rif1Δ*   ■ *sml1Δ mec1Δ*

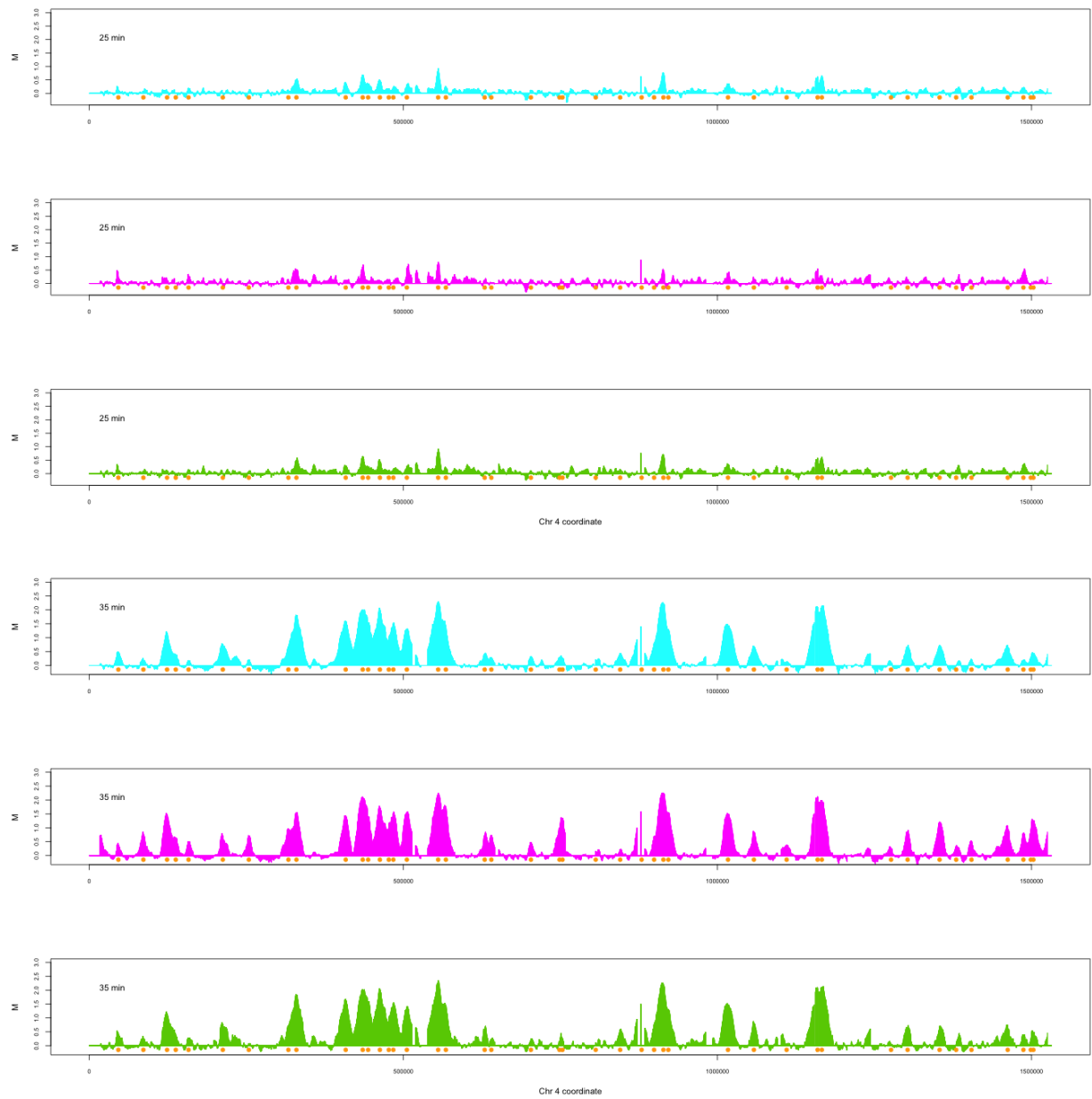

Supplement: Figure S4 — Temporal analysis of replication by BrdU-IP-chip for all chromosomes. Plots show average BrdU incorporation from duplicate experiments. Plot colors are keyed above. (ZIP) [file pone.0098501.s004.zip › FigS4/S4.4.pdf]

■ *sml1Δ*   ■ *sml1Δ rif1Δ*   ■ *sml1Δ mec1Δ*

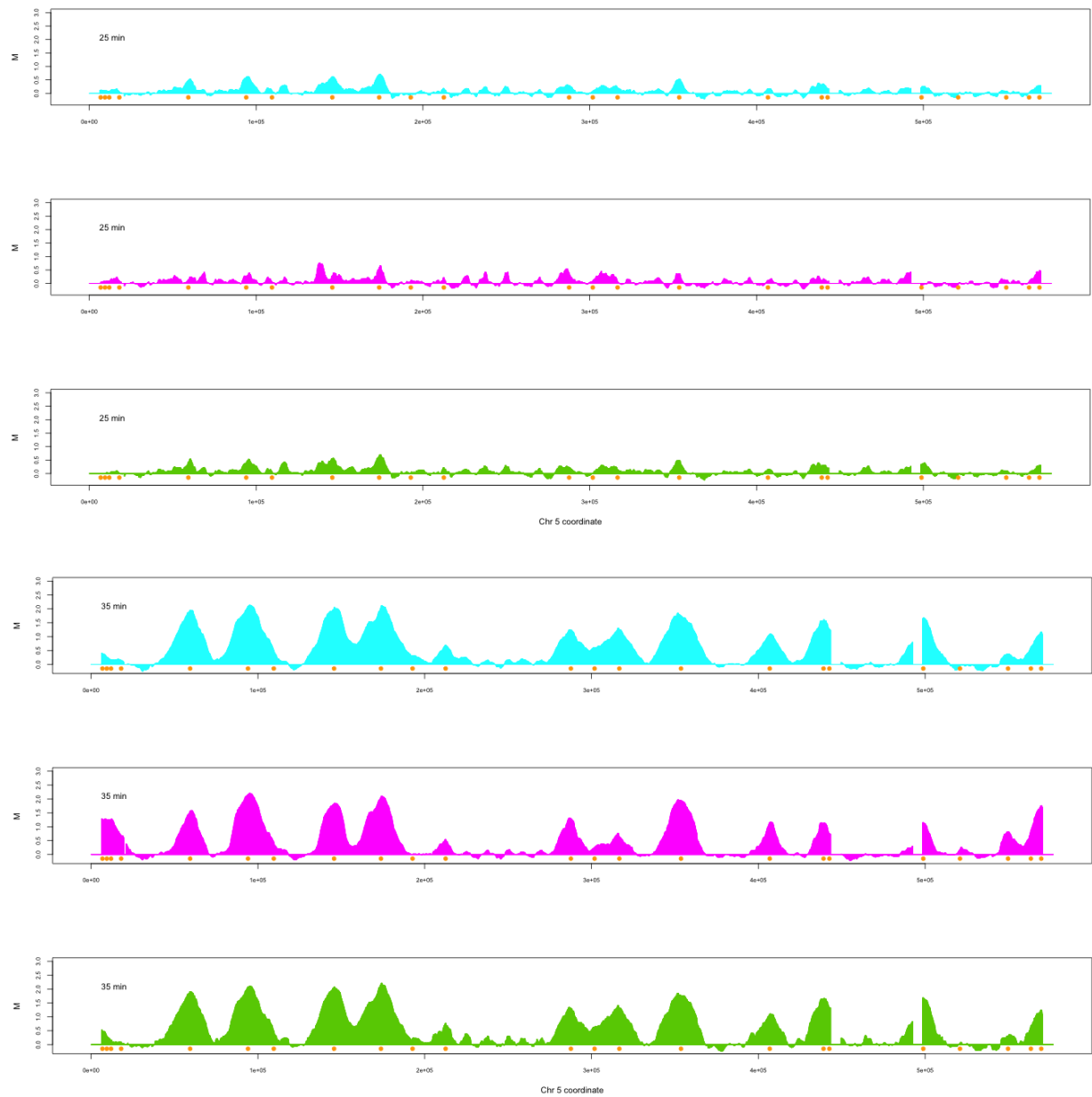

Supplement: Figure S4 — Temporal analysis of replication by BrdU-IP-chip for all chromosomes. Plots show average BrdU incorporation from duplicate experiments. Plot colors are keyed above. (ZIP) [file pone.0098501.s004.zip › FigS4/S4.5.pdf]

■ *sml1Δ*   ■ *sml1Δ rif1Δ*   ■ *sml1Δ mec1Δ*

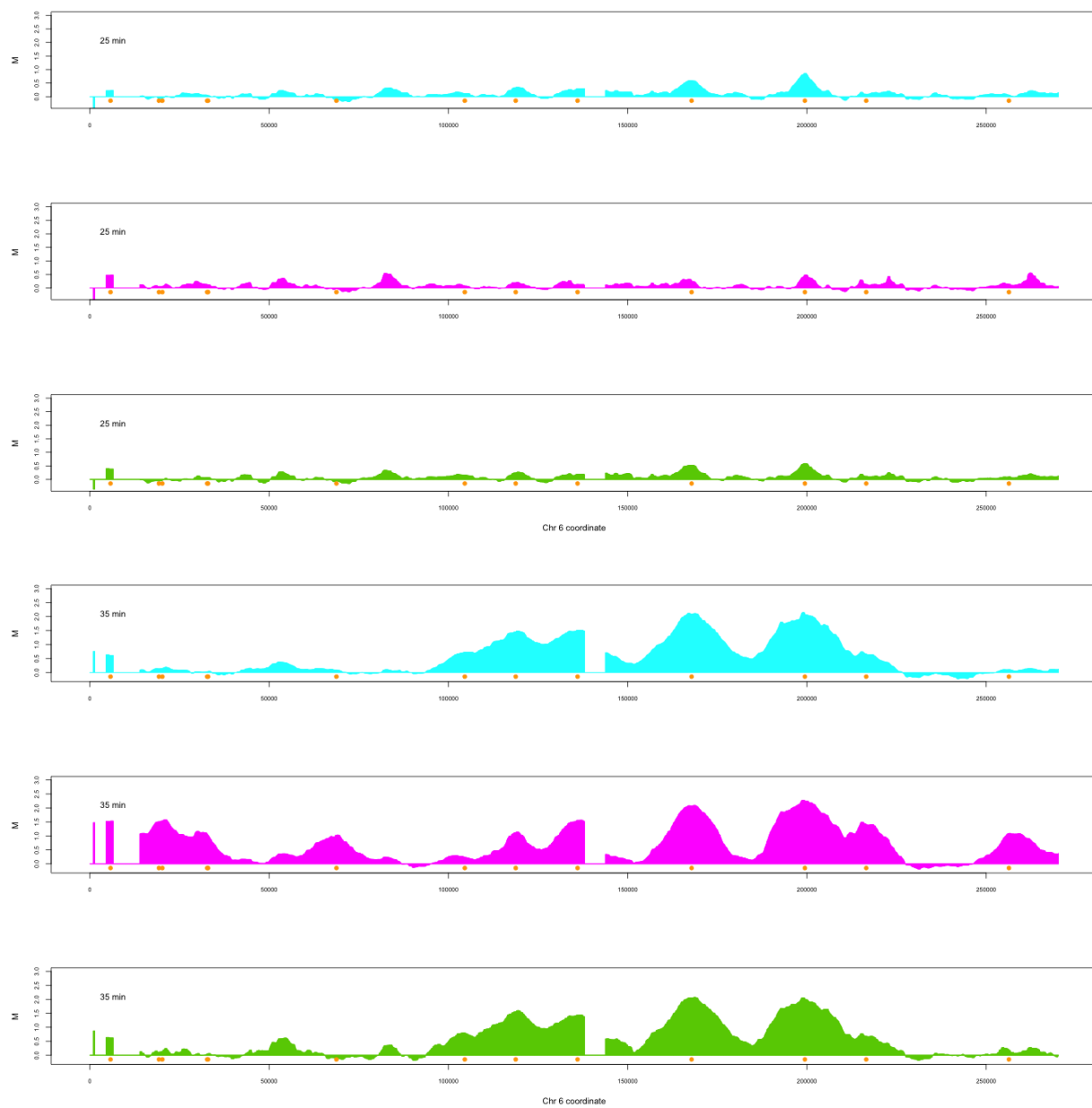

Supplement: Figure S4 — Temporal analysis of replication by BrdU-IP-chip for all chromosomes. Plots show average BrdU incorporation from duplicate experiments. Plot colors are keyed above. (ZIP) [file pone.0098501.s004.zip › FigS4/S4.6.pdf]

■ *sml1Δ*   ■ *sml1Δ rif1Δ*   ■ *sml1Δ mec1Δ*

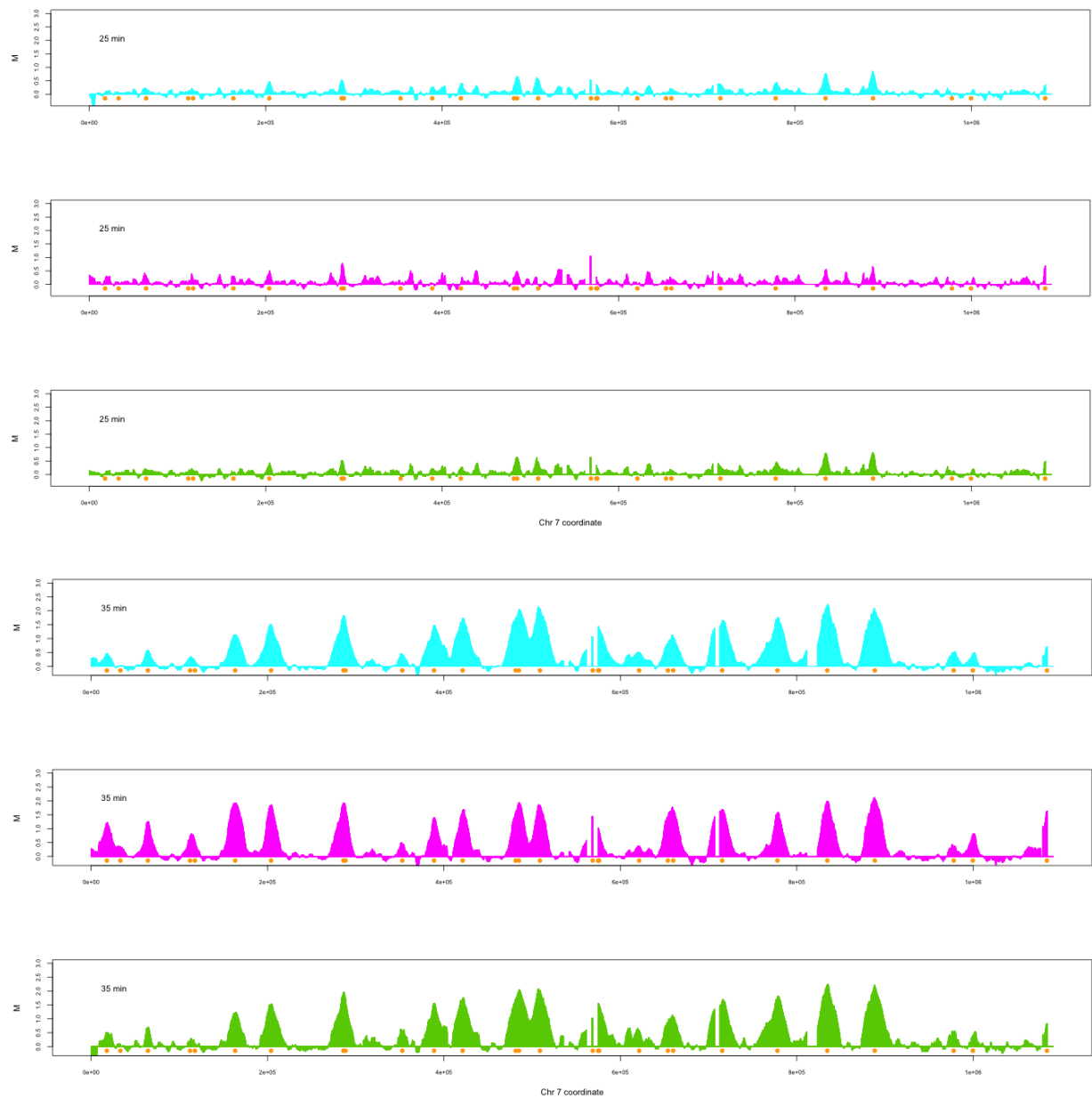

Supplement: Figure S4 — Temporal analysis of replication by BrdU-IP-chip for all chromosomes. Plots show average BrdU incorporation from duplicate experiments. Plot colors are keyed above. (ZIP) [file pone.0098501.s004.zip › FigS4/S4.7.pdf]

■ *sml1Δ*   ■ *sml1Δ rif1Δ*   ■ *sml1Δ mec1Δ*

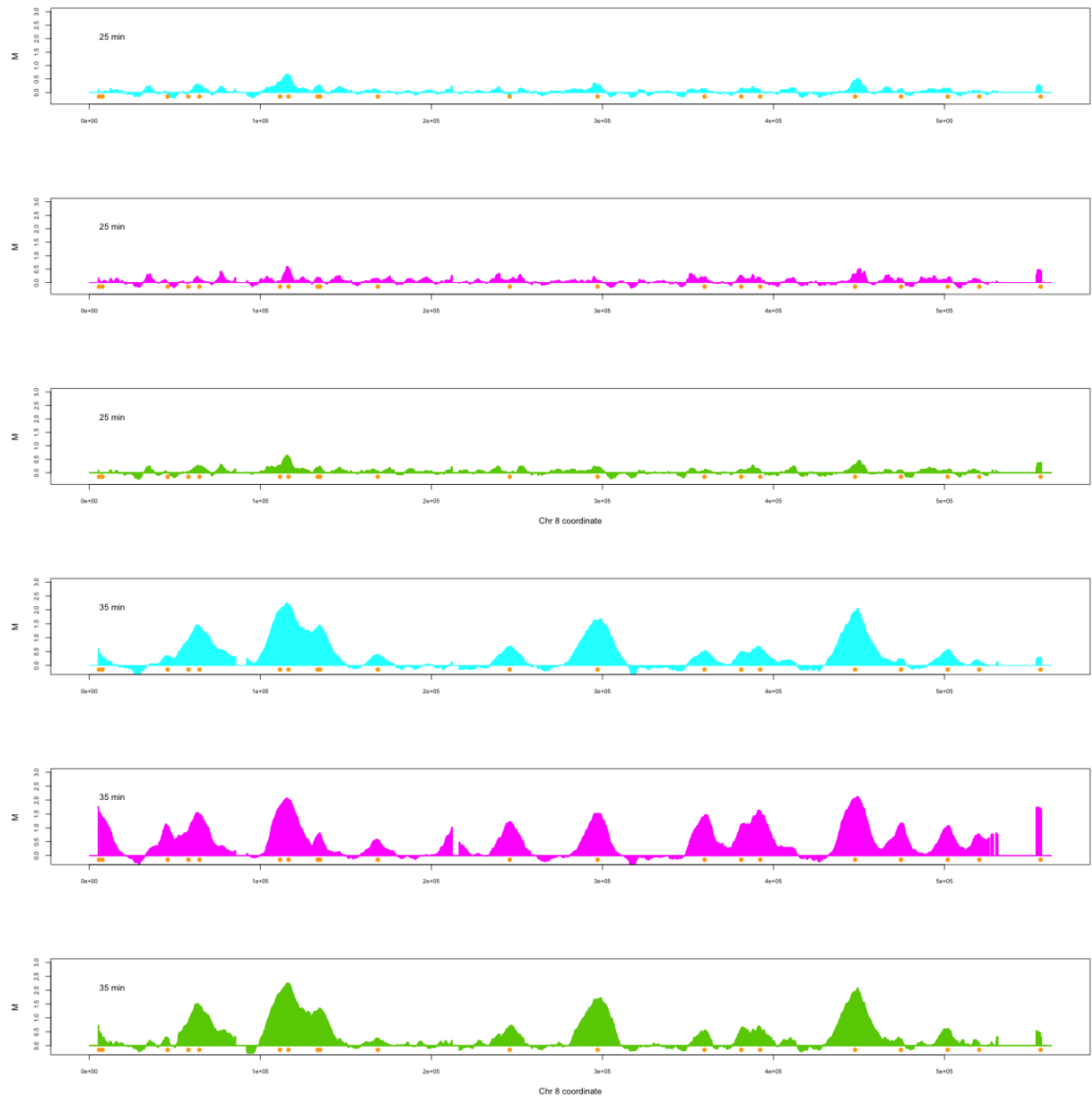

Supplement: Figure S4 — Temporal analysis of replication by BrdU-IP-chip for all chromosomes. Plots show average BrdU incorporation from duplicate experiments. Plot colors are keyed above. (ZIP) [file pone.0098501.s004.zip › FigS4/S4.8.pdf]

■ *sml1Δ*   ■ *sml1Δ rif1Δ*   ■ *sml1Δ mec1Δ*

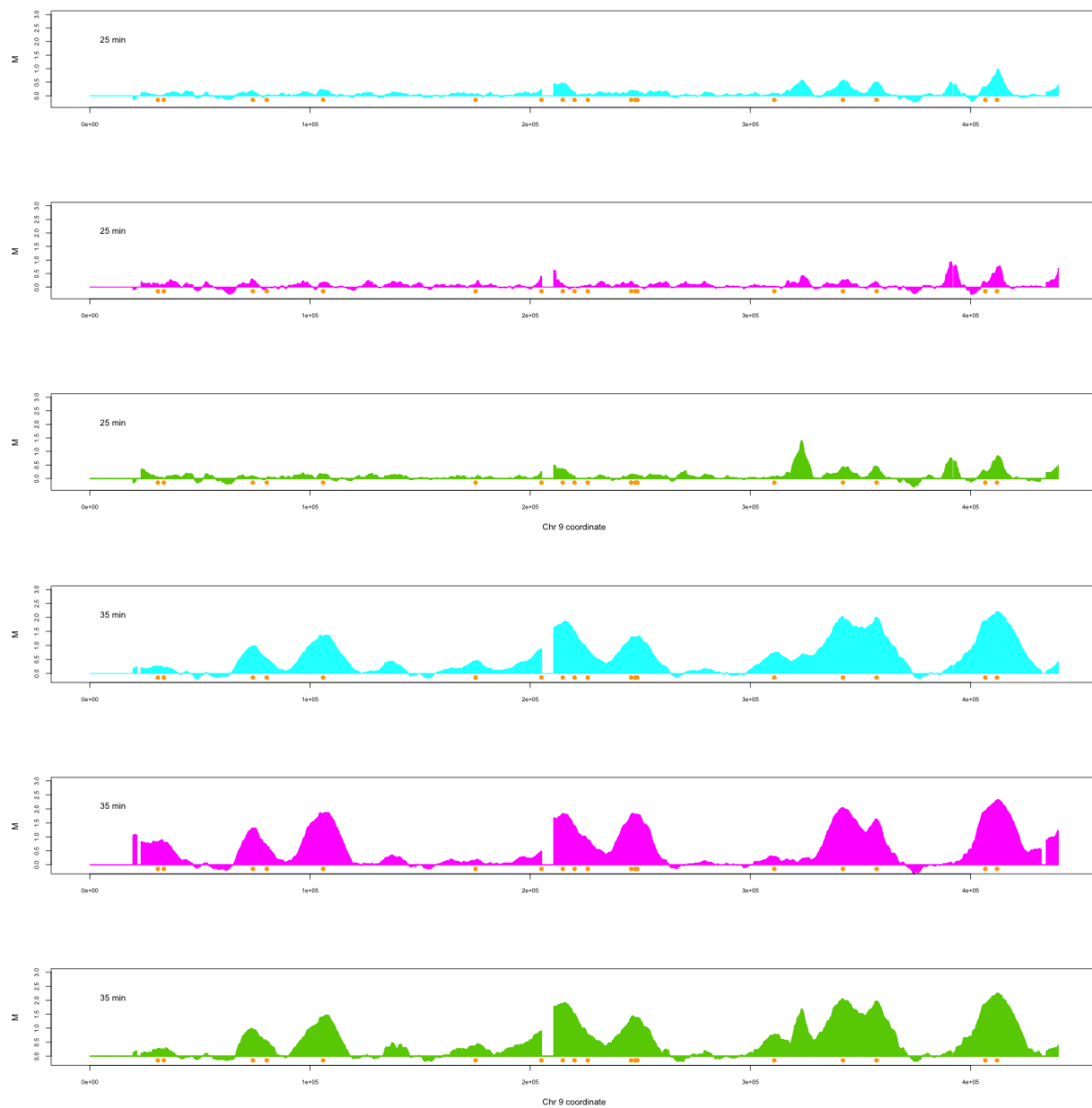

Supplement: Figure S4 — Temporal analysis of replication by BrdU-IP-chip for all chromosomes. Plots show average BrdU incorporation from duplicate experiments. Plot colors are keyed above. (ZIP) [file pone.0098501.s004.zip › FigS4/S4.9.pdf]

■ WT   ■ rif1Δ   ● Activated   ● Repressed   ● Unregulated

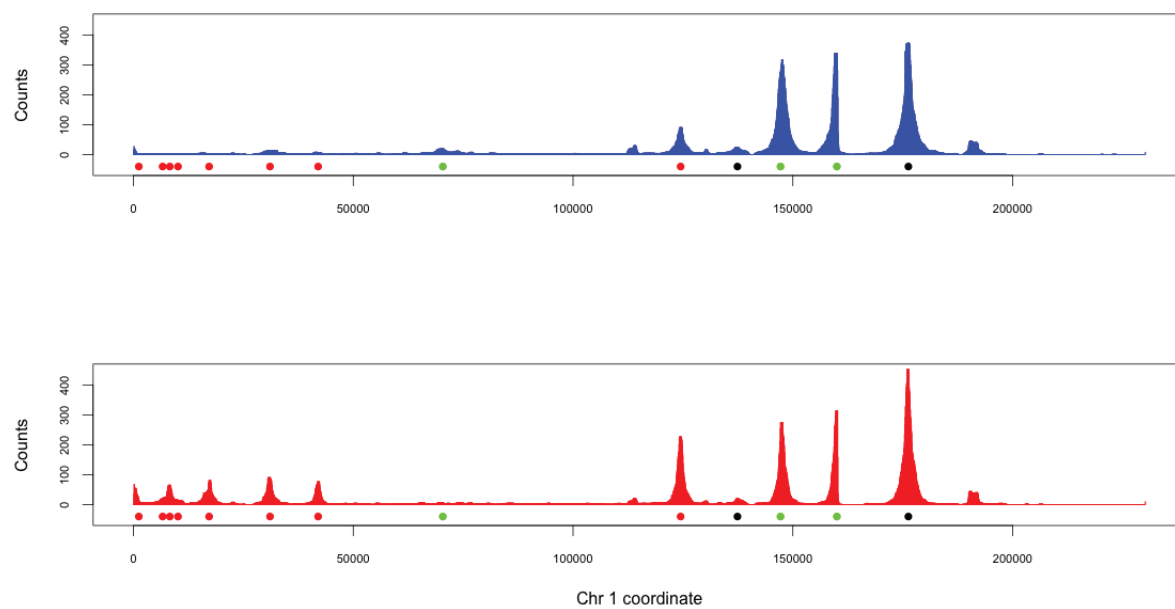

Supplement: Figure S5 — Analysis of early S-phase by BrdU-IP-Seq for all chromosomes. Plots show average BrdU incorporation from duplicate HU experiments. Origin classes are color-coded below each plot. Plot colors are keyed above. (ZIP) [file pone.0098501.s005.zip › FigS5/S5.1.pdf]

■ WT      ■ rif1Δ      ● Activated      ● Repressed      ● Unregulated

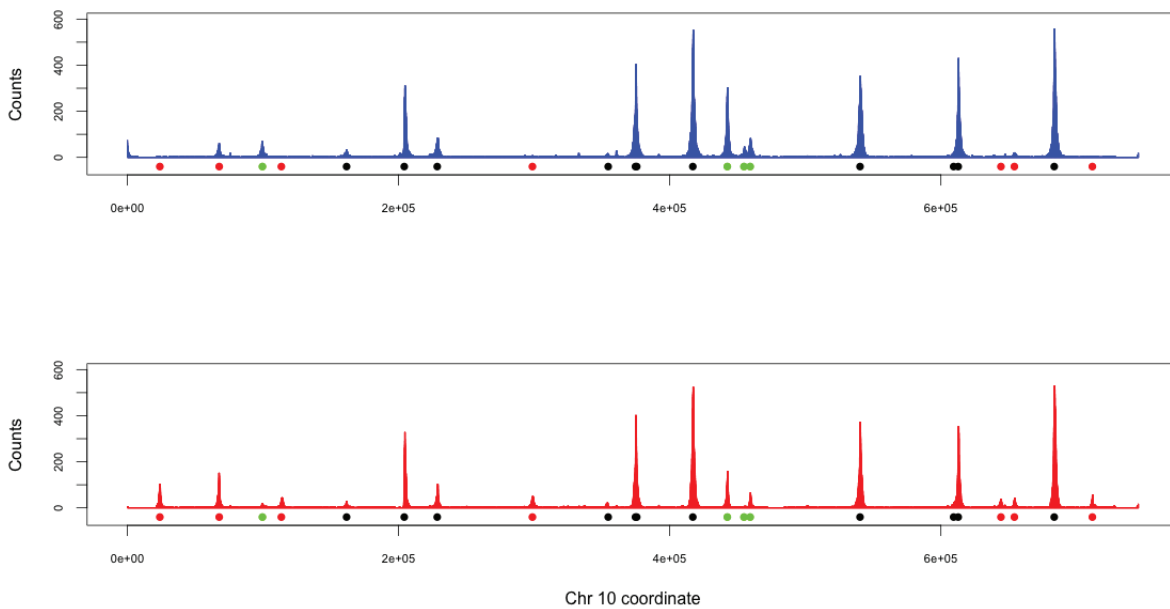

Supplement: Figure S5 — Analysis of early S-phase by BrdU-IP-Seq for all chromosomes. Plots show average BrdU incorporation from duplicate HU experiments. Origin classes are color-coded below each plot. Plot colors are keyed above. (ZIP) [file pone.0098501.s005.zip › FigS5/S5.10.pdf]

■ WT      ■ rif1Δ      ● Activated      ● Repressed      ● Unregulated

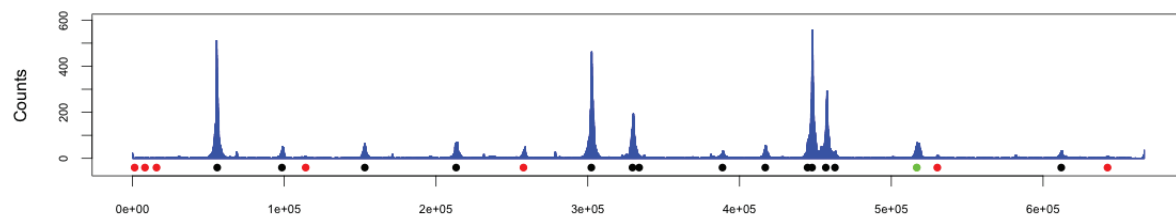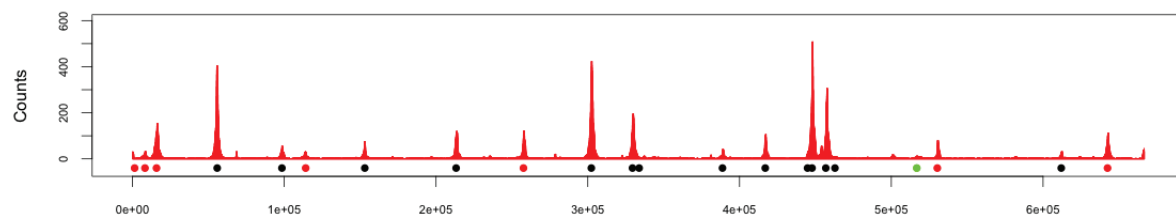

Chr 11 coordinate

Supplement: Figure S5 — Analysis of early S-phase by BrdU-IP-Seq for all chromosomes. Plots show average BrdU incorporation from duplicate HU experiments. Origin classes are color-coded below each plot. Plot colors are keyed above. (ZIP) [file pone.0098501.s005.zip › FigS5/S5.11.pdf]

■ WT   ■ rif1Δ   ● Activated   ● Repressed   ● Unregulated

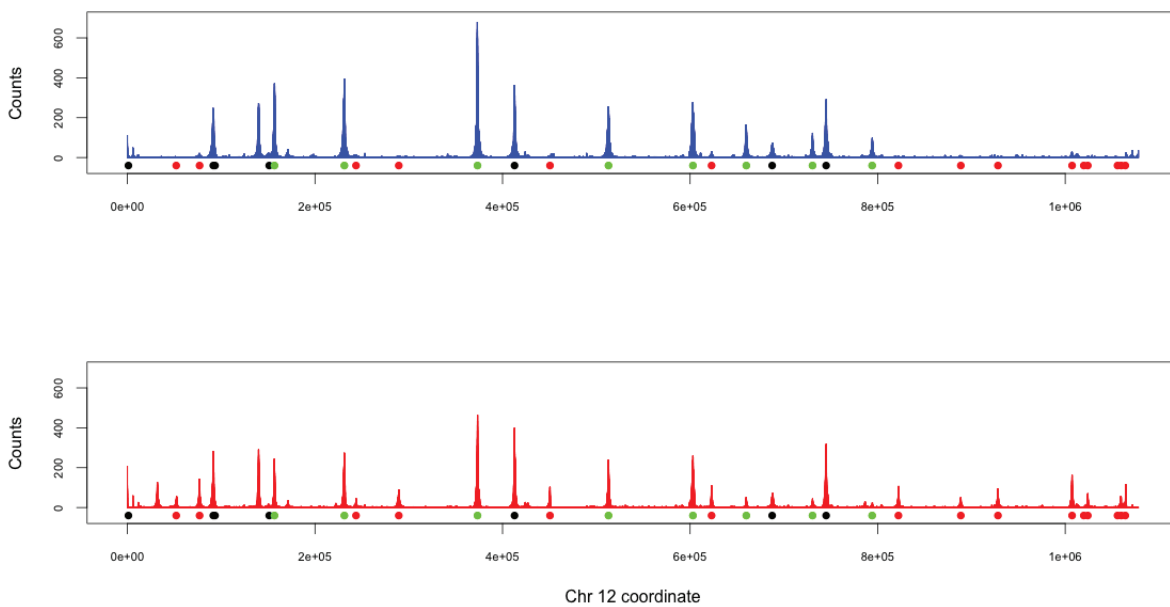

Supplement: Figure S5 — Analysis of early S-phase by BrdU-IP-Seq for all chromosomes. Plots show average BrdU incorporation from duplicate HU experiments. Origin classes are color-coded below each plot. Plot colors are keyed above. (ZIP) [file pone.0098501.s005.zip › FigS5/S5.12.pdf]

■ WT      ■ rif1Δ      ● Activated      ● Repressed      ● Unregulated

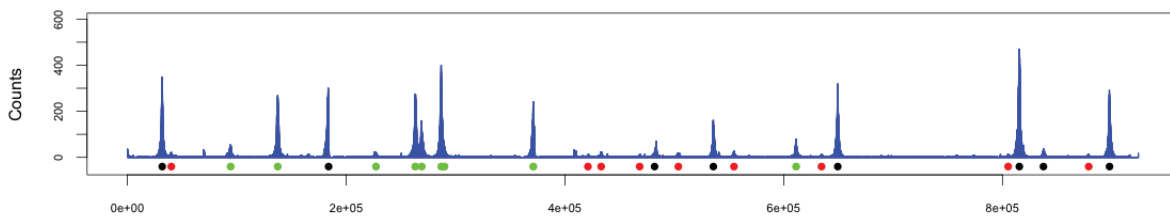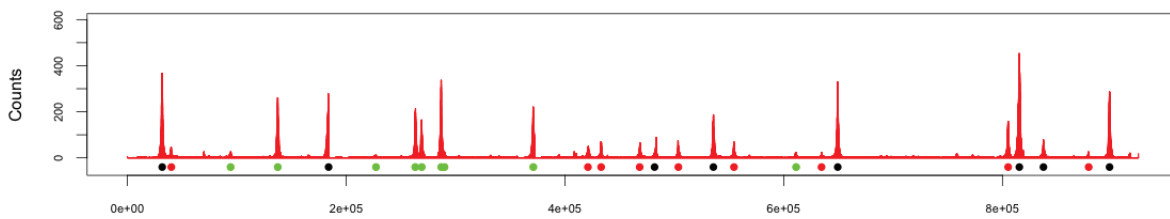

Chr 13 coordinate

Supplement: Figure S5 — Analysis of early S-phase by BrdU-IP-Seq for all chromosomes. Plots show average BrdU incorporation from duplicate HU experiments. Origin classes are color-coded below each plot. Plot colors are keyed above. (ZIP) [file pone.0098501.s005.zip › FigS5/S5.13.pdf]

■ WT      ■ rif1Δ      ● Activated      ● Repressed      ● Unregulated

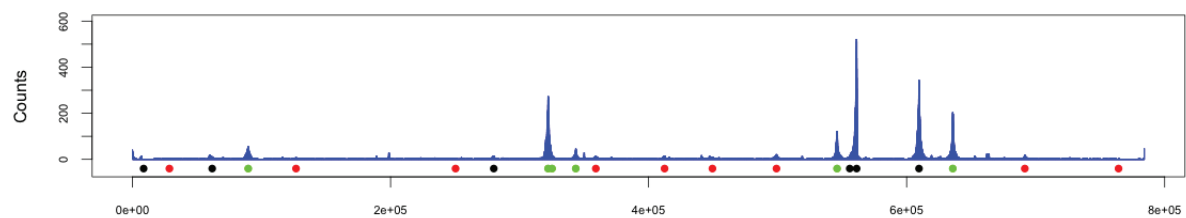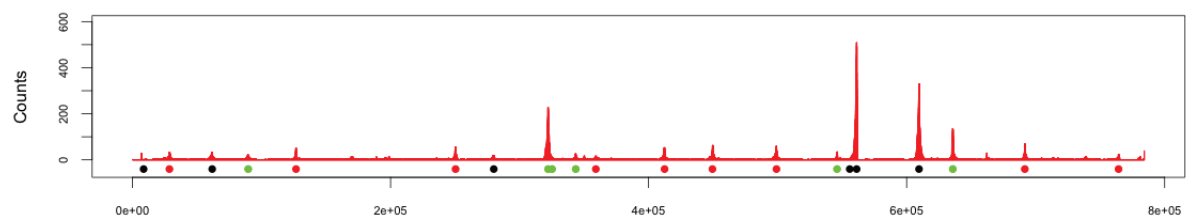

Chr 14 coordinate

Supplement: Figure S5 — Analysis of early S-phase by BrdU-IP-Seq for all chromosomes. Plots show average BrdU incorporation from duplicate HU experiments. Origin classes are color-coded below each plot. Plot colors are keyed above. (ZIP) [file pone.0098501.s005.zip › FigS5/S5.14.pdf]

■ WT      ■ rif1Δ      ● Activated      ● Repressed      ● Unregulated

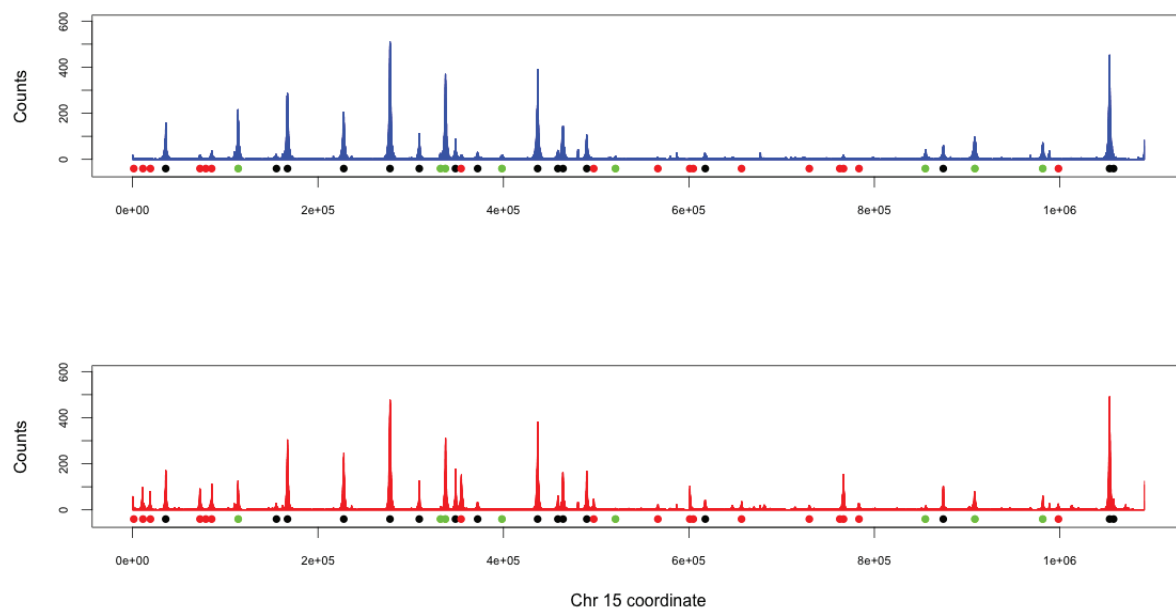

Supplement: Figure S5 — Analysis of early S-phase by BrdU-IP-Seq for all chromosomes. Plots show average BrdU incorporation from duplicate HU experiments. Origin classes are color-coded below each plot. Plot colors are keyed above. (ZIP) [file pone.0098501.s005.zip › FigS5/S5.15.pdf]

■ WT   ■ rif1Δ   ● Activated   ● Repressed   ● Unregulated

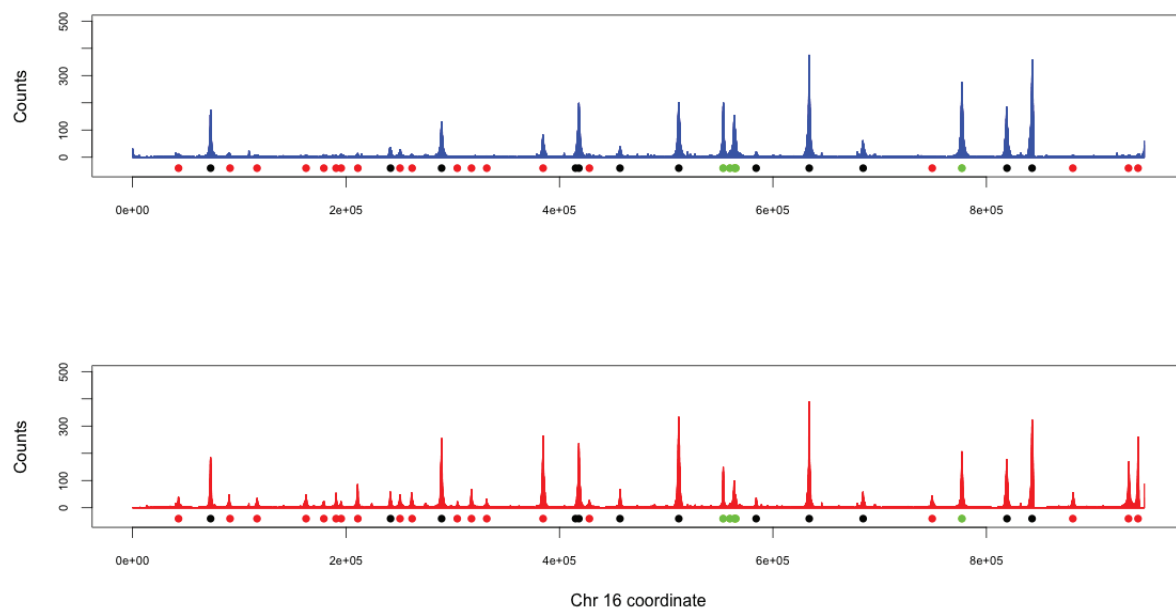

Supplement: Figure S5 — Analysis of early S-phase by BrdU-IP-Seq for all chromosomes. Plots show average BrdU incorporation from duplicate HU experiments. Origin classes are color-coded below each plot. Plot colors are keyed above. (ZIP) [file pone.0098501.s005.zip › FigS5/S5.16.pdf]

■ WT   ■ rif1Δ   ● Activated   ● Repressed   ● Unregulated

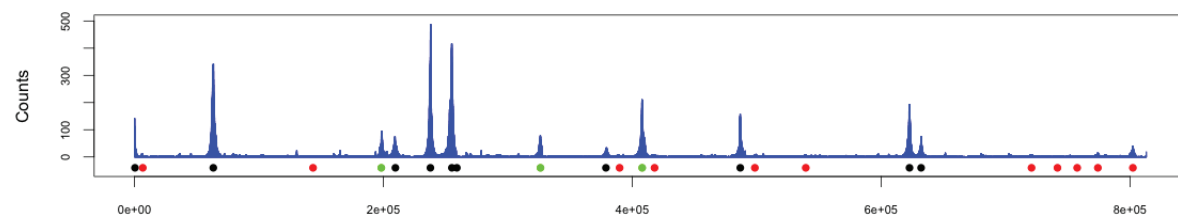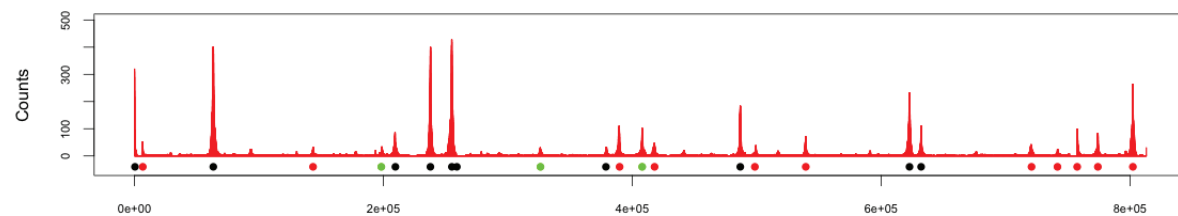

Chr 2 coordinate

Supplement: Figure S5 — Analysis of early S-phase by BrdU-IP-Seq for all chromosomes. Plots show average BrdU incorporation from duplicate HU experiments. Origin classes are color-coded below each plot. Plot colors are keyed above. (ZIP) [file pone.0098501.s005.zip › FigS5/S5.2.pdf]

■ WT   ■ rif1Δ   ● Activated   ● Repressed   ● Unregulated

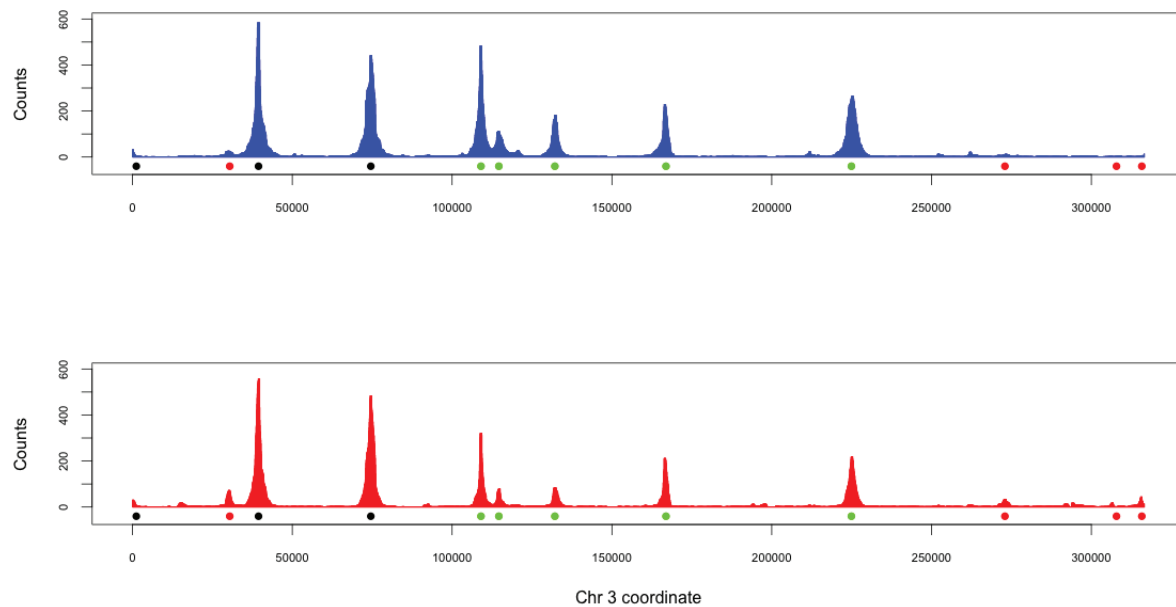

Supplement: Figure S5 — Analysis of early S-phase by BrdU-IP-Seq for all chromosomes. Plots show average BrdU incorporation from duplicate HU experiments. Origin classes are color-coded below each plot. Plot colors are keyed above. (ZIP) [file pone.0098501.s005.zip › FigS5/S5.3.pdf]

■ WT   ■ rif1Δ   ● Activated   ● Repressed   ● Unregulated

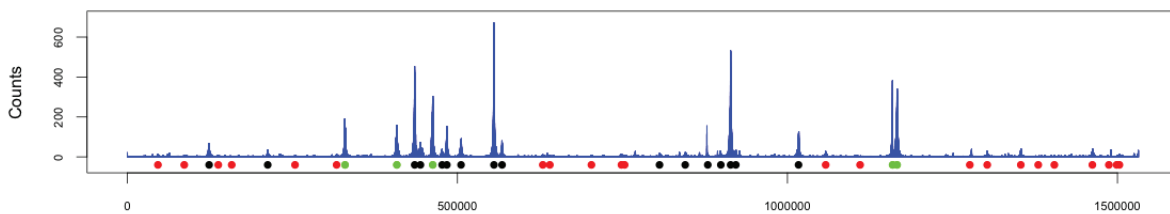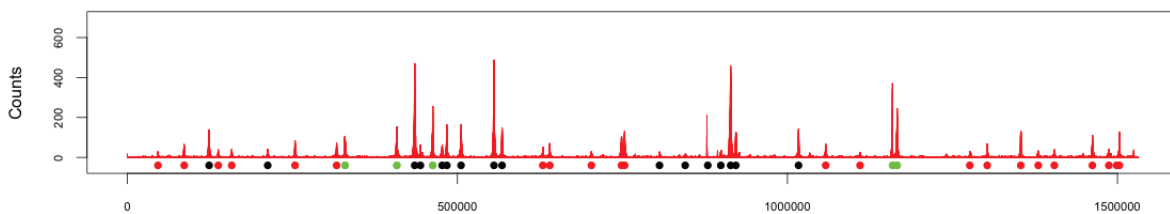

Chr 4 coordinate

Supplement: Figure S5 — Analysis of early S-phase by BrdU-IP-Seq for all chromosomes. Plots show average BrdU incorporation from duplicate HU experiments. Origin classes are color-coded below each plot. Plot colors are keyed above. (ZIP) [file pone.0098501.s005.zip › FigS5/S5.4.pdf]

■ WT      ■ rif1Δ      ● Activated      ● Repressed      ● Unregulated

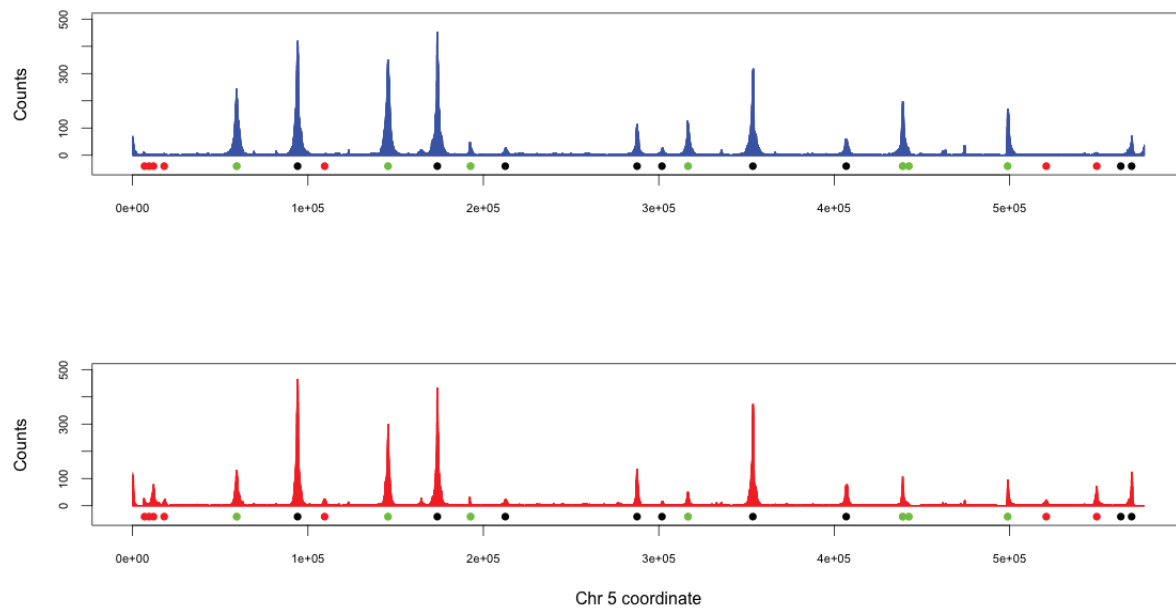

Supplement: Figure S5 — Analysis of early S-phase by BrdU-IP-Seq for all chromosomes. Plots show average BrdU incorporation from duplicate HU experiments. Origin classes are color-coded below each plot. Plot colors are keyed above. (ZIP) [file pone.0098501.s005.zip › FigS5/S5.5.pdf]

■ WT   ■ rif1Δ   ● Activated   ● Repressed   ● Unregulated

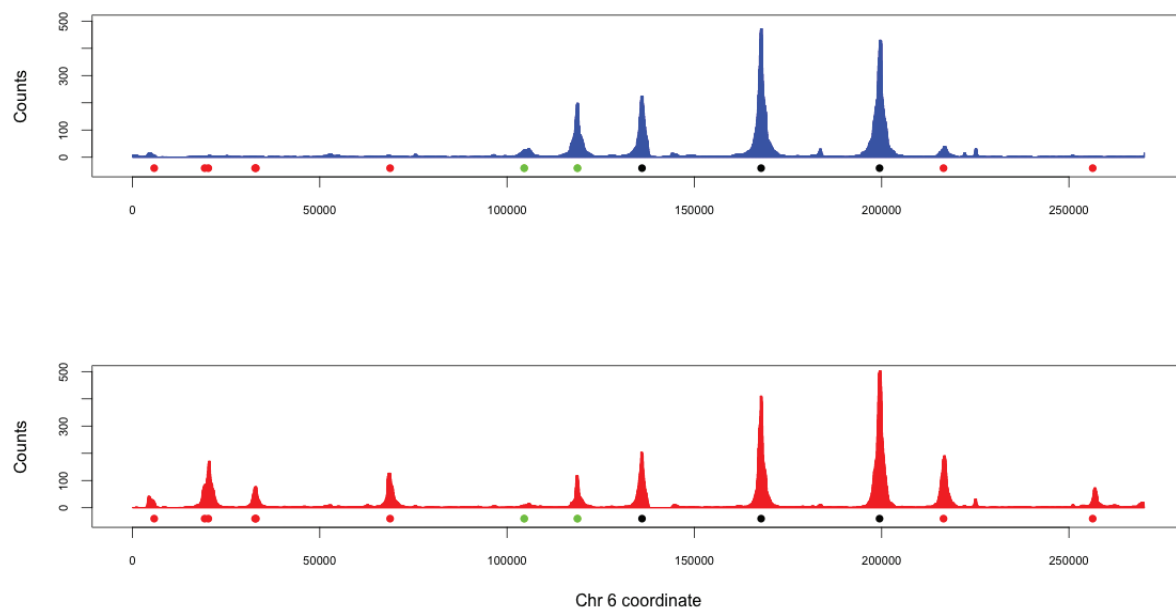

Supplement: Figure S5 — Analysis of early S-phase by BrdU-IP-Seq for all chromosomes. Plots show average BrdU incorporation from duplicate HU experiments. Origin classes are color-coded below each plot. Plot colors are keyed above. (ZIP) [file pone.0098501.s005.zip › FigS5/S5.6.pdf]

■ WT   ■ rif1Δ   ● Activated   ● Repressed   ● Unregulated

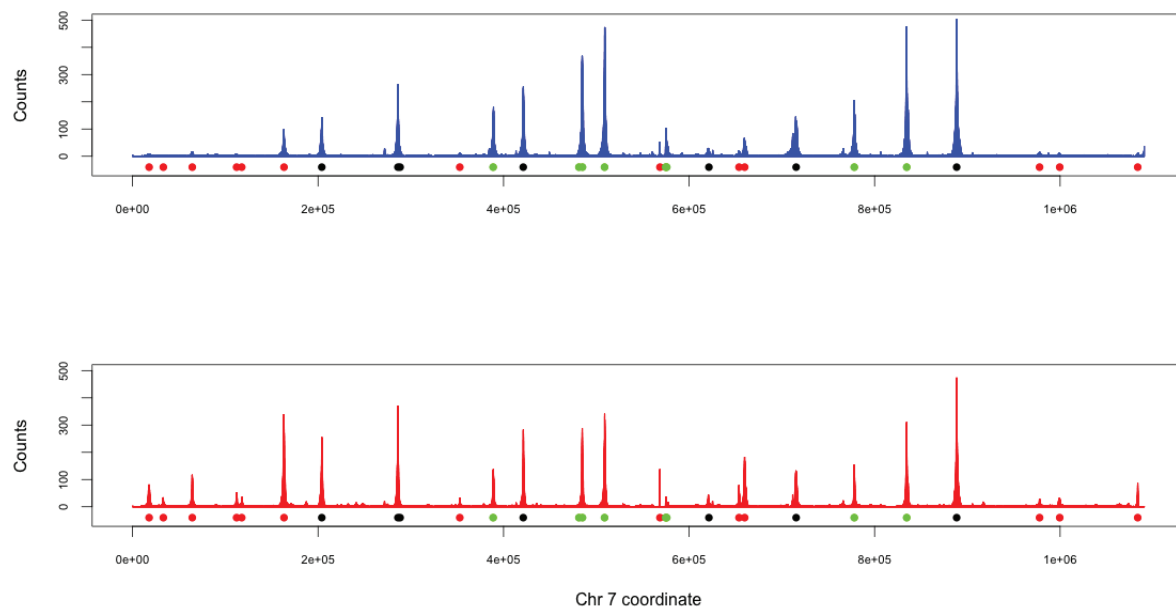

Supplement: Figure S5 — Analysis of early S-phase by BrdU-IP-Seq for all chromosomes. Plots show average BrdU incorporation from duplicate HU experiments. Origin classes are color-coded below each plot. Plot colors are keyed above. (ZIP) [file pone.0098501.s005.zip › FigS5/S5.7.pdf]

■ WT      ■ rif1Δ      ● Activated      ● Repressed      ● Unregulated

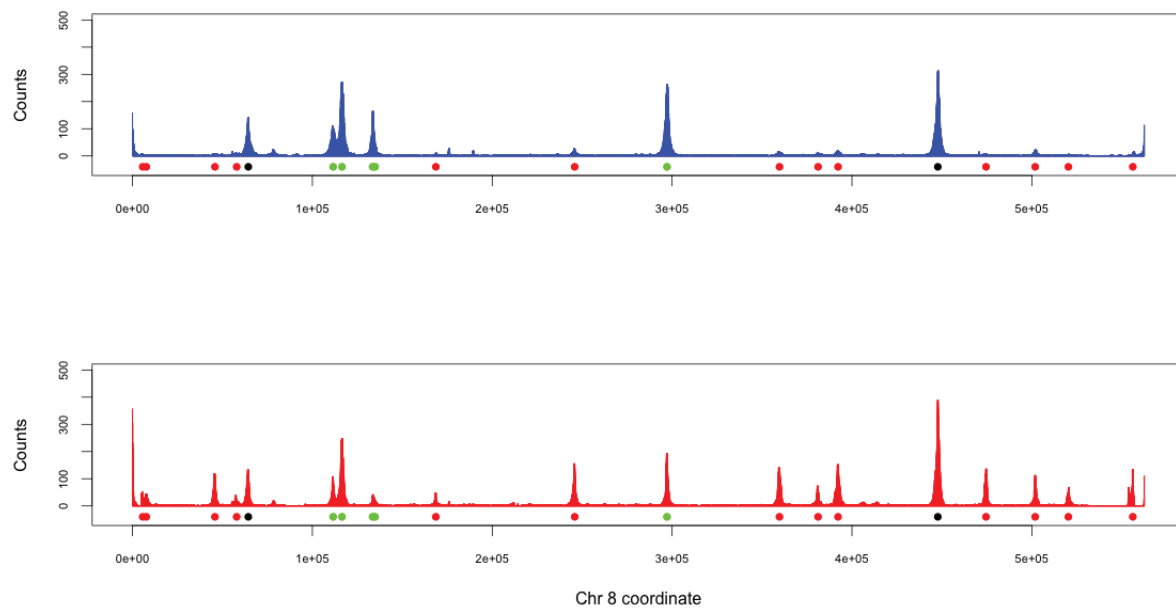

Supplement: Figure S5 — Analysis of early S-phase by BrdU-IP-Seq for all chromosomes. Plots show average BrdU incorporation from duplicate HU experiments. Origin classes are color-coded below each plot. Plot colors are keyed above. (ZIP) [file pone.0098501.s005.zip › FigS5/S5.8.pdf]

■ WT   ■ rif1Δ   ● Activated   ● Repressed   ● Unregulated

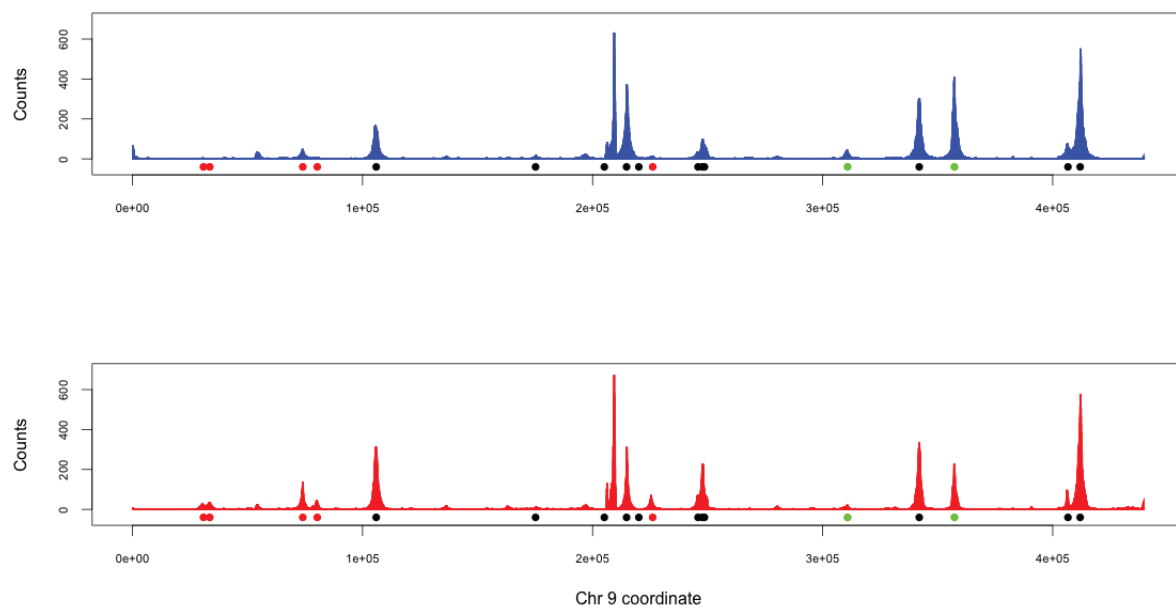

Supplement: Figure S5 — Analysis of early S-phase by BrdU-IP-Seq for all chromosomes. Plots show average BrdU incorporation from duplicate HU experiments. Origin classes are color-coded below each plot. Plot colors are keyed above. (ZIP) [file pone.0098501.s005.zip › FigS5/S5.9.pdf]
